# Benchmarking plant single cell RNA-sequencing sample processing strategies

**DOI:** 10.1038/s44318-026-00800-5

**Published:** 2026-05-09

**Authors:** Thomas Eekhout, Lindsy De Veirman, Jolien De Block, Freya Persyn, Vera Goossens, Dominique Audenaert, Gert Van Isterdael, Bert De Rybel, Carolin Grones

**Affiliations:** 1 Department of Plant Biotechnology and Bioinformatics, Ghent University, Ghent, Belgium; 2 VIB Center for Plant Systems Biology, Ghent, Belgium; 3 VIB Single Cell Core, VIB, Ghent/Leuven, Belgium; 4 VIB Screening Core, Ghent, Belgium; 5 Ghent University Centre for Bioassay Development and Screening (C-BIOS), Ghent, Belgium; 6 VIB Flow Core, VIB Center for Inflammation Research, Ghent, Belgium; 7 Department of Biomedical Molecular Biology, Ghent University, Ghent, Belgium; 8 Laboratory of Cell and Developmental Biology, Cluster of Plant Developmental Biology, Department of Plant Sciences, Wageningen University, Wageningen, The Netherlands

**Keywords:** Chromatin, Transcription & Genomics, Methods & Resources, Plant Biology

## Abstract

The isolation of single plant cells from complex tissues is prone to selective enrichment and sampling biases, which complicates accurate profiling of the large diversity in cell types. Optimizing methodologies for cell enrichment and single-cell transcriptomics is therefore critical for single-cell studies addressing plant cell heterogeneity. Here, we systematically compared protoplast enrichment technologies (including conventional and image-based flow cytometry, as well as magnetic cell sorting) and single-cell RNA sequencing (scRNA-seq) platforms (10X Genomics Chromium, BD Rhapsody) using Arabidopsis roots. Image-based flow cytometry offered increased precision due to customizable gating strategies, while magnetic sorting provided faster processing and enhanced representation of cell size heterogeneity. Both scRNA-seq platforms captured root cell heterogeneity and yielded reproducible gene expression profiles, but showed platform-associated differences in cell type composition. Notably, single-nucleotide polymorphism analysis of a mixed ecotype sample revealed that, among cells identified as doublets by computational algorithms, two-thirds were likely to have been misclassified. These insights identify key biases in plant cell purification and scRNA-seq workflows and provide practical guidance for improving data quality across plant species.

## Synopsis

Isolation of single plant cells for genomic analysis is challenging and prone to selective sampling biases due to the cell wall. This Resource provides extensive benchmarking of plant-specific sample processing strategies for enhanced single-cell RNA sequencing and data quality considerations.

- Magnetic levitation enables rapid enrichment of viable *Arabidopsis* root protoplasts whilst preserving cell-size heterogeneity.
- Customizable gating ensures precision in image-based flow cytometry.
- 10x Chromium and BD Rhapsody show broadly comparable mapping performance and sample quality metrics.
- 10x Chromium and BD Rhapsody differ in captured cell type composition and gene detection sensitivity necessitating cautious dataset integration.
- SNP-based analysis of mixed ecotypes reveals over-classification by standard doublet-detection tools, highlighting the need for plant-tailored doublet assessment and filtering.

## Abstract (web summary)

This Resource offers a data-driven guide to circumvent biases and establish best-practice in plant single-cell transcriptomics experiments.

## Graphical abstract

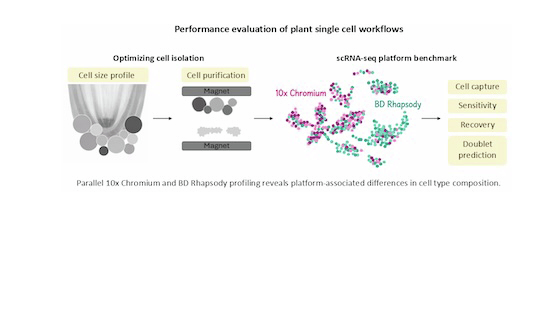

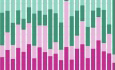

## Introduction

Single-cell RNA sequencing (scRNA-seq) has revolutionized our understanding of plant development and molecular adaptation to changing conditions. Since the first plant scRNA-seq studies in 2016 (Efroni et al, 2016), and the subsequent implementation of high-throughput scRNA-seq methods in 2019 (Denyer et al, 2019; Jean-Baptiste et al, 2019; Ryu et al, 2019; Shulse et al, 2019; Turco et al, 2019; Zhang et al, 2019), Arabidopsis root development has emerged as a primary model system for plant single-cell transcriptomics due to its intricate cellular heterogeneity and well-defined developmental gradients (Wendrich et al, 2020; Gala et al, 2021; Roszak et al, 2021; Yang et al, 2021; Apelt et al, 2022; Otero et al, 2022; Shahan et al, 2022; Han et al, 2024; Lyu et al, 2025). Building upon the success of Arabidopsis root studies, significant advancements in plant cell and nuclei isolation protocols have enabled the application of scRNA-seq to increase the knowledge of root development in a diverse set of biological contexts, including e.g., comparative root development in monocots and dicots (Liu et al, 2021; Guillotin et al, 2023; Ke et al, 2025), root adaptation mechanisms (Omary et al, 2022; Cantó-Pastor et al, 2024; Jo et al, 2025), and root development and biotic interactions (Cervantes-Pérez et al, 2022; Frank et al, 2023; Liu et al, 2023; Pereira et al, 2024).

While the application of scRNA-seq is becoming more standardized in plant research, single-cell technology is rapidly evolving, with significant technological advancements. Although high-throughput scRNA-seq methods appear to offer similar data outputs, inherent differences between platforms can lead to cell type-specific performance variations (Ziegenhain et al, 2017; Gao et al, 2020; Yamawaki et al, 2021; Colino-Sanguino et al, 2024; Salcher et al, 2024). This raises concerns about the interchangeability of platforms and underscores the importance of considering platform choice based on the specific biological question. For instance, 10X Genomics Chromium (droplet-based microfluidics) and BD Rhapsody (microwell-based cell capture), two widely used 3’-scRNA-seq platforms, employ distinct cell capture mechanisms. Both systems utilize cell barcodes and unique molecular identifiers (UMIs) for cell identification and transcript quantification, yet differences in cell compartmentalization strategy, RNA-capture bead chemistry, cell barcode and UMI architecture, and RNA processing workflows lead to significant variations in performance using human/animal cell cultures and heterogeneous tissues (Gao et al, 2020; Salcher et al, 2024). Despite these findings, comparative analyses of scRNA-seq methods in plants are lacking to date. This is particularly concerning given the unique challenges posed by plant protoplasts, including size variability, enzymatic stress responses, and fragility. Moreover, the broad diversity of plant species, tissues, and experimental designs necessitates an evaluation of scRNA-seq platforms within plant contexts.

In this study, we systematically compare sample preparation methods for scRNA-seq workflows, as well as commonly used scRNA-seq platforms. We identify platform-associated limitations in cell type and state capture, as well as gene capture sensitivity. Furthermore, we demonstrate the imprecision of doublet assignment by standard detection tools in our samples. This study thus provides insights into the suitability of single-cell sequencing platforms for plant research, guiding future methodological advancements in plant single-cell transcriptomics.

## Results

### Preserving cell size heterogeneity during sample purification

**Figure 1:**
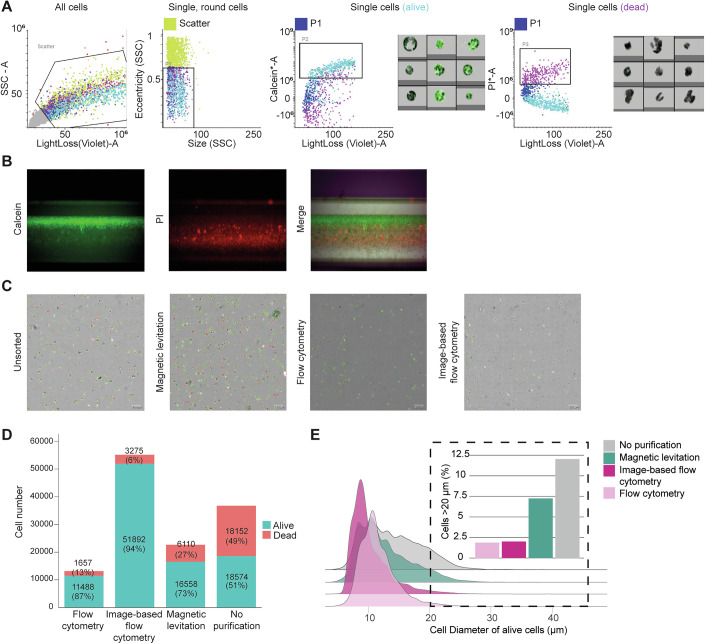
Cell enrichment strategies and their effect on cell size heterogeneity. (**A**) Gating strategy used to enrich alive cells with image-based flow cytometry (BD FACSDiscover^TM ^S8). The left panel (“All cells”) shows all events in a dot plot of side scatter (SSC, *y* axis) versus LightLoss(Violet) (Lightloss(Violet)-A, *x* axis). Lightloss is an image-derived parameter that measures the reduction in incident laser light caused by scattering and absorption as the cell passes the laser beam at the interrogation point (Schraivogel et al, 2022). It serves as a proxy to forward scatter but provides improved definition on the boundary of the cell. The polygon gate selects intact cells based on size and granularity, excluding smaller debris. The middle panel (“Single, round cells”) displays events from this gate plotted by image-derived eccentricity (*y* axis) versus image-derived cell size (*x* axis); the P1 gate enriches round, single cells by excluding doublets and aggregates, which show higher eccentricity values (greater than 0.6). Within the P1 population, alive single cells (light blue) are identified by high calcein AM fluorescence intensity and confirmed by corresponding cell images (panel “Single cells (alive)”), while dead single cells (purple) are identified by high Propidium Iodide (PI) fluorescence intensity and likewise confirmed by images (panel “Single cells (dead)”). (**B**) Representative images for the magnetic levitation-based cell enrichment assay. Alive cells accumulate in the top fraction (stained with calcein AM (left panel)), while dead cells and debris deposit into a lower fraction (stained with propidium iodide (PI, middle panel)). (**C**) Representative images used to quantify cell size heterogeneity in protoplast suspensions without purification, and after live cell enrichment by magnetic levitation and flow cytometry. (**D**) Quantification of the total number of alive (calcein-positive, green bars) and dead (PI-positive, orange bars) cells after magnetic levitation-based cell enrichment, (image-based) flow cytometry, and without further purification. Data represent two independent biological replicates, each performed in technical triplicates. (**E**) Quantification of cell diameters (in μm) after magnetic levitation-based cell enrichment (green), (image-based) flow cytometry (magenta), and without further purification (gray). The bar graph shows the percentage of large cells (defined with a cell diameter above 20 μm) in each sample. Data represent *n* = 2 independent biological replicates, each performed in technical triplicates. Source data are available online for this figure.

The process of extracting protoplasts (living cells without cell walls) from roots involves mechanical and enzymatic digestion that often results in a significant amount of debris. Cell debris (which includes fragments of dead cells, organelles, and degraded RNA) interferes with the cell compartmentalization and contaminates the sample with non-specific (ambient) RNA. This contamination can lead to false positive signals, where RNA fragments from dead or dying cells are mistakenly attributed to viable cells. Furthermore, debris can interfere with the efficiency of cDNA synthesis during library preparation, as fragmented RNA may not be efficiently converted into cDNA. Thus, the removal of debris and dead cells ensures that only viable, healthy cells are collected and analyzed. We therefore investigated the purity and applicability of cell enrichment and sorting strategies to increase sample purity for plant single-cell transcriptomics (Fig. 1).

Flow cytometry is a widely used method for high-throughput sorting and enrichment of protoplasts, as it allows distinguishing between living cells, dead cells, and debris particles (Fig. 1A). Despite its efficiency, flow cytometry prolongs the sample preparation duration and can be stressful to cells due to the high pressure used during sorting, thereby damaging them or compromising cell viability. In addition, flow cytometry has physical limitations regarding the size of cells it can handle. Gentle cell separation technologies, such as those based on magnetic levitation (Durmus et al, 2015), offer a promising alternative to minimize cellular stress and maintain cell viability, integrity, and functionality during the sorting process. This system, though not supporting fluorophore-based enrichment, provides a fast (5-15 min) and low-pressure (<1 psi) method for separating viable protoplasts from dead cells and debris, irrespective of cell size (Fig. 1B). Furthermore, magnetic cell enrichment proved effective in preserving cell type representation across a range of plant tissues, including wheat root (Ke et al, 2025) and Arabidopsis leaves (Tenorio Berrío et al, 2025), and exhibited wide compatibility with various plant species and tissues (Dataset EV1).

**Figure EV1:**
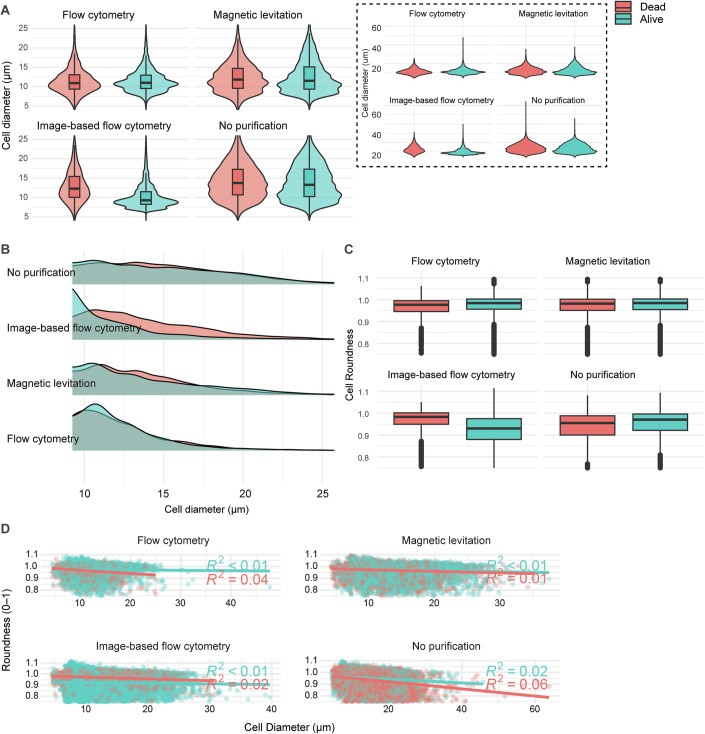
Cell diameter and roundness in viable and non-viable cells after purification. (**A**) Violin plots showing the distribution and mean of cell diameters for viable (calcein-positive, turquois) and non-viable (PI-positive, orange) cells after image-based flow cytometry, magnetic levitation, or without purification. Embedded boxplots indicate the median (central line, 50th percentile), with the box spanning the 25th (Q1) to 75th (Q3) percentiles, representing the interquartile range (IQR) and the middle 50% of the data. Whiskers extend to the most extreme values within 1.5 × IQR from Q1 and Q3. The inset on the right shows the full diameter range. (**B**) Density plots of the same diameter measurements as in A), highlighting differences in the frequency of specific size ranges. (**C**) Box plots with the mean of cell roundness for viable and non-viable cells after purification and in the unsorted sample. The central line represents the median (50th percentile), the box spans the 25th (Q1) to 75th (Q3) percentiles, encompassing the middle 50% of the data. Whiskers extend to the most extreme values within 1.5 × IQR from Q1 and Q3. Individual points beyond the whiskers are plotted as dots to indicate outliers. (**D**) Scatter plots of cell roundness versus diameter for viable and non-viable cells under each condition. The low *r*² values indicate no appreciable correlation between cell size and roundness in any condition. Data represent two independent biological replicates, each performed in technical triplicates.

We next evaluated the applicability and precision of the different cell enrichment strategies for root protoplasts from *A. thaliana* (Fig. 1C,D). Protoplasts from a single input sample were either not processed further or ran through a classical cell sorter, a live image-enabled cell sorter, or a magnetic levitation-based cell separation method (Fig. 1C). We utilized calcein AM and propidium iodide (PI) to clearly differentiate living and dead cells. Cell concentration, viability, and size distributions were quantified either using semi-automated image processing or manual measurements (Fig. 1D,E). After cell sorting, viability increased to 94% and 87% for image-based and regular flow cytometry, respectively, compared to 51% viability in the non-purified sample (Fig. 1D). Viability after magnetic levitation reached 73%. To estimate the effect of flow cytometry versus magnetic levitation-based cell separation on preserving the original cell size heterogeneity among root cells, we quantified protoplast size (defined as cell diameter) distributions after applying different enrichment strategies (Fig. 1E). Magnetic levitation outperformed both flow cytometers in maintaining the original size heterogeneity of viable protoplasts (Fig. 1E; Dataset EV2): viable Arabidopsis root cells purified by magnetic separation displayed a broad and representative range of cell sizes (mean cell diameter = 12.6 µm; cells larger than 20 µm accounted for 7% of all analyzed cells), comparable to the unsorted sample (mean cell diameter = 14 µm; cells larger than 20 µm accounted for 12% of all analyzed cells), whereas both flow cytometers yielded a narrower size range biased toward smaller cells (mean cell diameter = 10.3/11.6; cells larger than 20 µm accounted for only 2% of the population). In unsorted samples and after magnetic levitation, viable and dead cells showed similar cell diameter distribution and roundness (Fig. EV1A–C), with no correlation between these parameters (Fig. EV1D). After image-based flow cytometry sorting, alive cells were smaller than dead cells, and dead cells showed reduced roundness (Fig. EV1A–C). Because no differences were observed in the unpurified samples, and diameter and roundness were uncorrelated in all conditions (Fig. EV1D), these changes likely result from shearing forces during sorting rather than pre-existing large or irregular aggregates.

Together, these findings thus suggest that magnetic levitation-based cell separation preserves cell size heterogeneity better than flow cytometry-based methods. In turn, this can lead to more reliable insights into the diverse phenotypes and functions present in root cell types, specifically for the rare large and more differentiated cells in the protoplast mixture.

### Benchmark comparison of scRNA-seq platforms: data preprocessing

**Figure 2:**
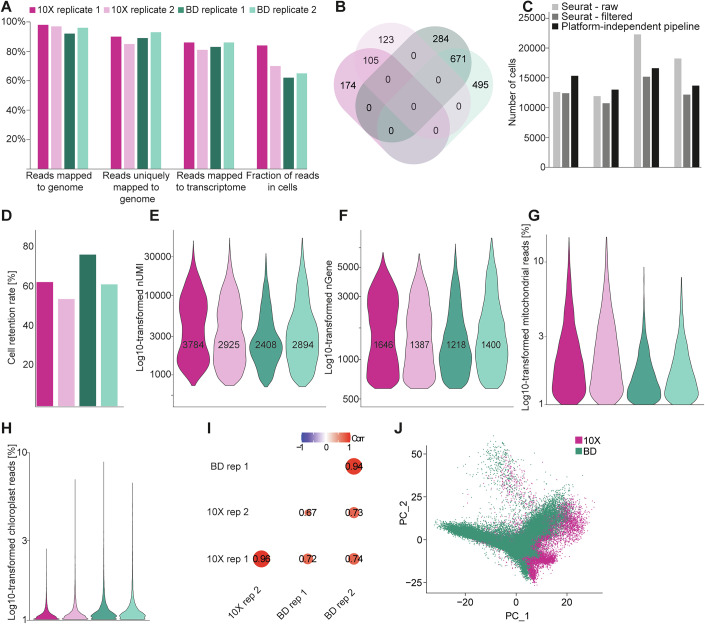
Platform performance evaluation of data quality, sensitivity, and reproducibility. (**A**) Summary of mapping rates of 10X Chromium replicates (magenta) and BD Rhapsody replicates (green). The data shown were derived from two independent biological replicates. (**B**) Venn diagram depicting uniquely detected genes in each replicate. (**C**) High-quality cells after filtering with Seurat and a technology-independent pipeline. (**D**) Cell retention rate (in %) for both 10X Chromium and BD Rhapsody replicates. Cell retention is defined as the percent of cells that remained after quality filtering from all captured cells. (**E**–**H**) Violin plots showing the number of expressed genes and UMI detected per cell, and the percentage of mitochondrial and chloroplast read content per cell. Data shown are from *n* = 2 biological replicates. (**I**) Pearson correlation coefficient analysis showing the variability in expression of the top 3000 variable genes between both platforms and among the technical replicates. The top 3000 variable genes were defined based on their expression variance across all cells from the four samples. (**J**) Principal component analysis (PCA) plot, where each point represents a single cell, colored by the technology used (10X Chromium vs. BD Rhapsody). Source data are available online for this figure.

In order to best compare technology platforms for scRNA-seq on plant samples, we opted for the well-studied and characterized Arabidopsis root apical meristem sample (6-day-old root meristems, cut 0.5 cm from tip). We reduced biological variation between samples by loading the identical sample as input simultaneously on both scRNA-seq platforms under study. Based on the results described above, we utilized magnetic levitation to enrich living cells from dead cells and debris. We prioritized sample purity over absolute cell viability, as reduced debris lowers the risk of chip clogging and improves signal-to-noise ratio, while low-quality cells can subsequently be removed during bioinformatic quality control filtering. In total, 20,000 cells from the same protoplast sample were simultaneously loaded as input on both the 10X Chromium (10X) and the BD Rhapsody (BD) platform and processed according to the standard sample processing protocols for each. To ensure the best possible comparison between results from both platforms, each library was sequenced to 150 M reads. Although the 10X pipeline (*Cell Ranger*) and BD pipeline (*Seven Bridges*) both use *STAR* to map reads to the genome (Salomon et al, 2019), they differ in how they generate their reference genome. Both start with the same gene annotation file, but BD applies additional filtering steps. Specifically, BD removes genes that lack an official gene symbol, genes classified as pseudogenes or ribosomal genes, and certain noncoding RNA genes that do not meet its inclusion criteria. In contrast, the 10X *Cell Ranger* pipeline retains a broader set of genes, including pseudogenes and various noncoding RNAs. As a result, the BD reference genome contains 31,109 genes- 3153 fewer than the 10X reference genome, which includes 34,262 genes. To avoid that these differences in reference genome would affect downstream analysis, the platform-independent algorithm *STARsolo* (Kaminow et al, 2021) was used to generate count matrices for all 10X and BD samples, using the Araport11 gene annotation containing 27,655 coding and 5178 noncoding genes (Dataset EV3). Both platforms showed comparable mapping efficiency (BD: 84.4%; 10X: 83.8%) (Fig. 2A), confirming robust alignment. Core transcriptome analysis revealed 23,410 shared genes (72% of Araport11), demonstrating platform consistency for the majority of Arabidopsis transcripts. For 10X Chromium, the two replicates shared 105 uniquely detected genes, with an additional 174 and 123 uniquely detected genes in replicates 1 and 2, respectively (Fig. 2B; Dataset EV4). For BD Rhapsody, the replicates shared 671 uniquely detected genes, with an additional 284 and 495 genes uniquely detected in replicates 1 and 2, respectively. In all, 30% of 10X-specific and 37% of BD-specific genes were expressed in more than 25 cells, indicating that many platform-associated genes are not restricted to a few rare cells but show relatively broad expression. No GO category reached statistical significance for the 10X-specific genes, whereas BD-specific genes showed significant enrichment for “RNA modification” (*P* = 0.03), including several Pentatricopeptide repeat (PPR) proteins, and for “protein binding” (*P* = 1 × 10^−^⁴), including, for example, *SCARECROW-LIKE 28*, *MYB101*, and *WUSCHEL*. The overall number of platform-associated genes was, however, compatible with random sampling variation rather than a strong, systematic platform bias in gene detection. These platform-associated gene sets are therefore best interpreted as reflecting subtle technical distinctions between BD Rhapsody and 10X Chromium (e.g., differences in cell/RNA capture, library preparation, and sensitivity to low-expression genes), as well as inherent variability in transcript recovery across cell types. Given the limited number of biological replicates (two per platform), we did not attempt formal between-replicate statistical testing.

Protoplast fragility and size variability pose significant challenges for scRNA-seq platforms. We next evaluated key parameters, such as cell number and gene content, to determine overall sample quality. We compared 10X Chromium and BD Rhapsody data after filtering for high-quality cells, obtaining 23,158 and 27,362 cells over both replicates, respectively (Fig. 2C), indicating a higher cell retention with BD Rhapsody (61-76%) versus 10X Chromium (53-62%) (Fig. 2D). 10X Chromium yielded higher median UMI (2925-3784) and gene (1387-1646) counts per cell compared to BD Rhapsody (2408-2894 UMIs, 1218-1400 genes) (Fig. 2E,F). These differences might suggest greater transcript capture sensitivity for 10X Chromium, but can also be partly explained by the higher sequencing depth per cell, as fewer cells were captured while both samples were sequenced at 150 M reads. To assess robustness to sequencing depth, we subsampled each dataset to 50 M, 100 M, and 150 M reads per sample and repeated the correlation analyses (Appendix Fig. S1). Genes per cell and UMIs per cell remained consistent across depths for both platforms (Appendix Fig. S1A,B), supporting comparable capture efficiency at matched sequencing depth. The fraction of genes detected per technology remained highly correlated across depths (Appendix Fig. S1C; 50 M: Pearson 0.877, Spearman 0.941; 100 M: Pearson 0.892, Spearman 0.944; 150 M: Pearson 0.899, Spearman 0.946), indicating that the increased transcript capture sensitivity for 10X is not solely driven by unequal sequencing depth or cell numbers. Both platforms exhibited low mitochondrial and chloroplast read percentages (Fig. 2G,H), confirming that the protoplast separation and processing were effective in minimizing cell death and contamination from chloroplasts for both platforms. Notably, 10X Chromium showed consistently higher mitochondrial read fractions, even after re-processing with an independent pipeline (Fig. 2G; Appendix Fig. S2; Dataset EV3).

To assess reproducibility, we evaluated biological replicates for each platform. We found high reproducibility for both platforms (*r*² = 0.94-0.96), whereas correlations between platforms were notably lower (*r*² = 0.72-0.73), indicating greater inter-platform variability (Fig. 2I). Principal component analysis (PCA) further confirmed distinct clustering of cells by technology, highlighting substantial differences in captured gene expression profiles (Fig. 2J). Thus, while both platforms exhibit robust technical reproducibility, 10X Chromium shows potentially higher transcript capture sensitivity and differences in mitochondrial read quantification, which may underlie the observed inter-platform variation in gene expression profiles. These results suggest that although each platform shows high reproducibility, caution is needed when combining datasets from different platforms.

### Benchmark comparison of scRNA-seq platforms: clustering and DEG analysis

**Figure 3:**
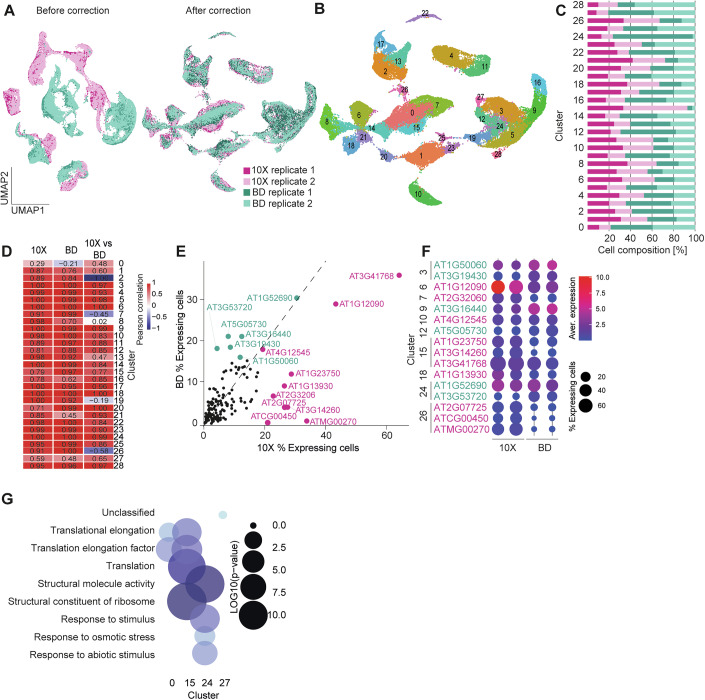
Inter-technology variability in cell cluster composition despite reproducible clustering. (**A**) Uniform manifold approximation and projection (UMAP) of the merged dataset prior to batch effect correction, with 10X Chromium replicates in magenta, and BD Rhapsody replicates in green. (**B**) UMAP of the merged dataset after batch effect correction, representing 28 clusters. (**C**) Contribution of cells (in percent) from the 10X Chromium replicates (magenta) and BD Rhapsody replicates (green) per cluster. Clusters 0, 15, 24, and 27 showed a technology bias in composition. Clusters with a technology bias were defined as those where one technology contributes less than 25% of cells. Clusters with roughly equal contributions from both technologies (40-50% per technology; e.g., Clusters 1, 2, 4, 7, 16, 20, 23) were considered unbiased. (**D**) Pearson correlation analysis of average expression of the top 5 marker genes of each cluster between 10X Chromium replicates, BD Rhapsody replicates, and 10X Chromium and BD Rhapsody. The normalized but non-batch-corrected count matrix was used to aggregate counts across cells to obtain a pseudo-bulk profile per sample, which was then correlated across replicates and platforms. (**E**) Correlation of the percentage of cells that express the top 5 marker genes of each cluster between 10X Chromium and BD Rhapsody. Highlighted genes are expressed in a higher number of cells derived from either 10X Chromium (magenta) or BD Rhapsody (green). (**F**) Average expression of outliers highlighted in (**E**). Circle size indicates percent of expressing cells. (**G**) GO-terms that are significantly enriched (*P* < 0.05; indicated by circle size) in the top 100 marker genes of clusters that showed a technology-biased composition. GO term enrichment was assessed using PANTHER (Fisher’s exact test, FDR-corrected). Source data are available online for this figure.

In single-cell transcriptomics, it is crucial to assess the potential batch effects that may influence the clustering of cells, as these effects can introduce both technical and biological biases (Yu et al, 2024). Batch effects refer to systematic differences that arise from non-biological factors, such as variations in experimental protocols, sample handling, or sequencing platforms, which can confound the identification of true biological signals. By incorporating biological replicates into our analysis, we aimed to distinguish clusters arising from technical variation from those consistently observed across replicates, reflecting true biological or platform-associated differences. As already indicated by the inter-platform variability in transcriptome capture (Fig. 2E), integration of all datasets indicated apparent platform-associated cell clustering (Fig. 3A). Batch correction with the *Harmony* algorithm was able to eliminate the technology bias, resulting in a total of 28 clusters that contained cells from each replicate and technology (Fig. 3B). In order to identify any potential platform-dependent clustering artifacts, we assessed the consistency of each cluster between the four samples. Clusters with roughly equal contributions from both platforms (40-50% per platform; e.g., clusters 1, 2, 4, 7, 16, 20, and 23) were considered unbiased (Fig. 3C). Most clusters showed a slight imbalance, with one platform contributing only 25-40% of the cells. By contrast, clusters 0, 15, 24, and 27 exhibited a marked bias, with one platform contributing less than 25% of the cells. We observed that cluster 15 was predominantly composed of cells from 10X Chromium (93% of all cells are from 10X Chromium, 7% of cells are from BD Rhapsody), while cluster 0 (79% of cells are from BD Rhapsody), cluster 24 (76% of cells are from BD Rhapsody), and cluster 27 (81% of cells are from BD Rhapsody) were predominantly composed of cells from BD Rhapsody. However, cluster 24 was predominantly enriched with cells from BD Rhapsody replicate 2, indicating that a technical artifact could not be excluded. These results indicate that the technology platform influences the final clustering of the data; and thus that the interpretation of clustering towards biological function needs to be made with caution. This result also suggests that experimental validation of clustering is important before deriving biological conclusions.

**Figure EV2:**
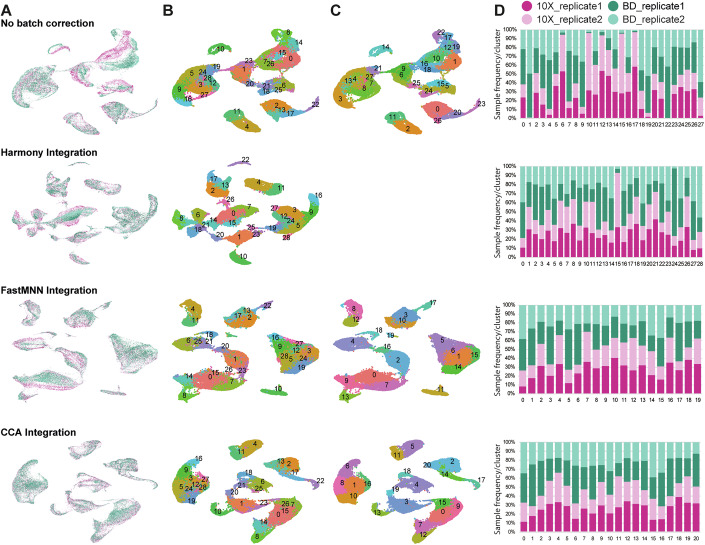
Cluster composition after batch correction. (**A**) UMAPs colored by platform of origin (10X, magenta; BD, green) for data processed without batch correction and after *Harmony*, *FastMNN*, or *CCA* integration. (**B**) The same UMAPs as in (**A**), colored by clusters defined within each respective integration workflow. (**C**) The same UMAPs as in (**A**), but with cluster labels transferred from the *Harmony*-derived clustering. (**D**) Bar plots showing, for each clustering scheme, the proportion of cells in each cluster originating from 10X (magenta) or BD (green).

To further investigate the impact of platform-associated biases on gene expression and biological interpretation, we correlated gene expression patterns of the top 5 cluster-specific marker genes across replicates and platforms (Fig. 3D; Dataset EV5). Gene expression of top marker genes remained highly correlated within replicates of both 10X Chromium and BD Rhapsody (with the exception of clusters 0, 21 for BD, and 27), whereas substantially lower correlations were observed when comparing the two platforms, particularly for clusters 2, 8, 19, and 26. As none of these clusters displayed a pronounced platform-biased cell composition, these platform-associated differences at the gene expression level cannot be attributed solely to differences in cell number or platform-biased composition and likely reflect residual platform-associated technical effects in cell and RNA capture. In line with this, we identified clusters in which the percentage of cells expressing the top 5 marker genes differed substantially between platforms (Fig. 3E), suggesting potential biases in gene detection or cell type representation. Notably, clusters with an even representation of cells from both platforms (e.g., clusters 6, 7, 9) also exhibited gene expression outliers (Fig. 3F), indicating platform-associated gene or process biases. These observations were robust to the choice of batch correction (no batch correction, *Harmony*, *CCA*, *MNN*; Fig. EV2), which yielded similar cluster structures and preserved the observed platform-associated differences in cluster-specific marker genes (Appendix Fig. S3). To further investigate the biological implications of these biases, we performed Gene Ontology (GO) enrichment analysis on the top 100 marker genes of clusters demonstrating technology-specific compositional biases (Fig. 3G). This analysis revealed significant enrichment (*P* < 0.05) of terms related to translation elongation (clusters 0 and 15) and osmotic stress response (cluster 24). These patterns could reflect differences in cellular stress or viability during sample processing and/or a comparatively better performance of BD Rhapsody in capturing and lysing cells in specific biological states. However, because neither platform permits selective processing and unambiguous tracing of individual cells, we cannot definitively discriminate between these possibilities.

**Figure EV3:**
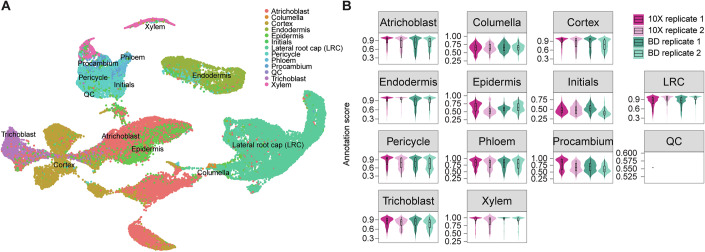
Cell type annotation. (**A**) UMAP visualization of the integrated dataset following batch effect correction, colored by annotated cell types. (**B**) Violin plots displaying prediction scores grouped by cell type. Cell origin is indicated by color: 10X Chromium replicates (magenta) and BD Rhapsody replicates (green). The central line in each boxplot represents the median (50th percentile), the box spans the interquartile range (25th-75th percentile), and whiskers extend to the most extreme values within 1.5 × the interquartile range (IQR). Data are from *n* = 2 biological replicates, with distributions showing individual cells.

**Figure 4:**
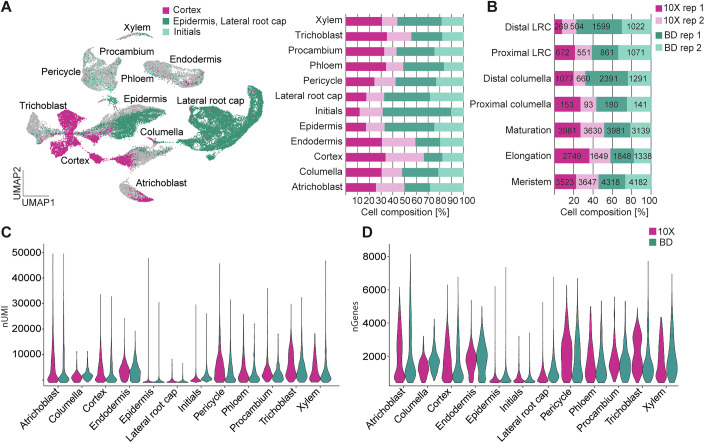
Technology-specific biases in cell type composition and gene detection. (**A**) UMAP showing different cell identities. Cell types demonstrating a notable bias in representation between the 10X Chromium and BD Rhapsody platforms are highlighted: cortex cells (magenta) are over-represented in 10X Chromium, whereas Epidermis and Lateral Root Cap cells (dark green) and cells classified as “Initials” (light green) are over-represented in BD Rhapsody replicates. The adjacent bar graph quantifies the relative contribution of each replicate (10X Chromium vs. BD Rhapsody) to the final cell type composition. (**B**) Bar graph illustrating the distribution of transcriptional cell states across the 10X Chromium (magenta) and BD Rhapsody (green) replicates. (**C**, **D**) Violin plots illustrate the distribution of unique molecular identifiers per cell (nUMI) and the number of genes detected per cell (nGenes) across each annotated cell type between 10X Chromium (magenta) and BD Rhapsody (green). Data shown are from *n* = 2 biological replicates. Source data are available online for this figure.

**Figure EV4:**
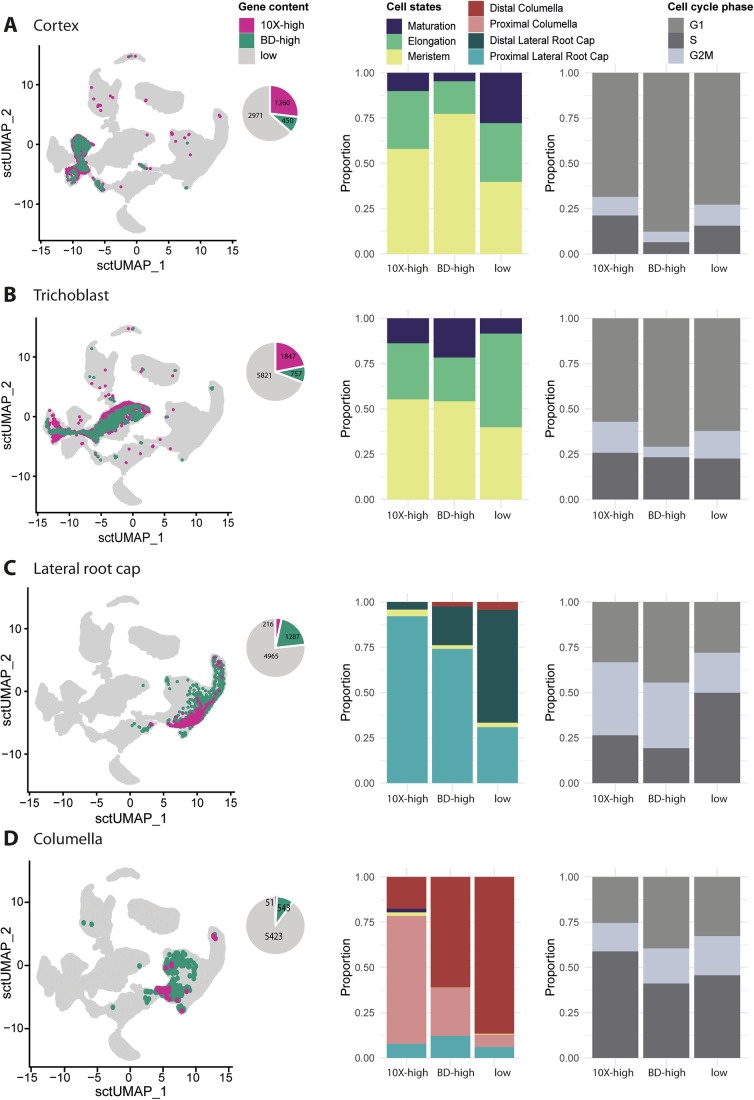
Platform-associated gene detection, cell state, and cell cycle phase distributions across major Arabidopsis root cell types. (**A**–**D**) Analysis of four representative root cell types: (**A**) Cortex, (**B**) Trichoblast, (**C**) Lateral root cap, and (**D**) Columella. For each cell type UMAP projections of single-cell transcriptomes are shown (left panel), with cells colored by gene detection category: high-gene-content in 10X Chromium (magenta), high-gene-content in BD Rhapsody (green), and low-gene-content (gray). Pie charts indicate the number of cells in each category for each cell type. Bar plots (middle panel) represent the proportion of major cell states within each gene content category (10X-high, BD-high, low), as defined by established marker genes from Shahan et al, 2022. Distinct colors represent different cell states relevant to each cell type. Bar plots in the right panel show the distribution of cell cycle phases (G1, S, G2M) within each gene content category.

In order to understand if these clustering differences result in changes in cluster annotation, we utilized established Arabidopsis cell identity marker genes (Wendrich et al, 2020; Shahan et al, 2022) to annotate the clusters. This analysis revealed a successful annotation of the major root cell types (excluding quiescent center cells) by both 10X Chromium and BD Rhapsody (Fig. EV3). However, we observed platform-associated biases in cell type representation (Fig. 4A). Specifically, BD Rhapsody yielded a higher proportion of epidermis, initials, and lateral root cap cells, while 10X Chromium enriched for cortex and endodermis cells. This platform-associated cell composition bias extended to developmental states, with BD Rhapsody capturing more proximal and distal lateral root cap cells, as well as distal columella cells (Fig. 4B). Morphology analysis of cells from epidermis, endodermis, cortex, and lateral root cap reporter lines showed that both lateral root cap and cortex cells exhibit a broader size range than endodermis and epidermis (Appendix Fig. S4; Dataset EV6), indicating that cell size limitations cannot be the sole explanation for the apparent platform-associated differences in capture efficiency. We next evaluated whether gene detection varied between platforms across different cell types (Fig. 4C,D). We noted that epidermis and initial cells consistently exhibited lower gene content compared to other cell types (Fig. 4D). Furthermore, notable platform-associated variations were observed: 10X Chromium captured more genes and higher UMI content in cortex, trichoblast, and pericycle cells, while BD Rhapsody excelled in columella, lateral root cap, and xylem cells. To explore potential biological sources of these variations, we focused on cortex, trichoblast, lateral root cap, and columella cells (Fig. EV4). Within each cell type, high gene content cells were selected and separately analyzed based on which platform they originated. We found that the high-gene-content cells captured by each platform seemed to be at different stages of development. This suggests that the reason we see different amounts of genes detected within the same cell type might be due to platform-associated enrichment of specific cell states, in which cells already adopted their transcriptome to perform its biological function. For example, 10X Chromium captured more high-gene-content cells from the cortex and trichoblast (Fig. EV4A), and when comparing developmental stage representations between high-gene-content cells from 10X to BD for these clusters, we saw that there were more cells associated with the elongation state in 10X (Fig. EV4A,B). This result indicates that more elongated cortex and trichoblast cells were captured with a high-gene-content in 10X than in BD. Conversely, BD Rhapsody captured more high-gene-content cells from lateral root cap and columella, which also represent more mature cell states such as distal lateral root cap and distal columella (Fig. EV4C,D), which both require an adaptation of their transcriptome to fulfill their biological function.

Taken together, our results demonstrate that while both platforms effectively capture major cell types, they exhibit distinct biases in cell type representation, developmental state capture, and transcript detection. These biases have significant implications for downstream analyses and should be carefully considered during experimental design and data interpretation.

### Computational algorithms underestimate true doublets formation

A persistent challenge in scRNA-seq analysis is the occurrence of doublets (instances where two or more cells are captured together in a single droplet or microwell). While homotypic doublets (from similar cell types) may have a limited impact, heterotypic doublets (from distinct cell types) can introduce misleading transcriptional profiles, distorting downstream analyses such as clustering and differential expression (Bernstein et al, 2020). Most doublet identification relies on computational transcriptome-based tools, which simulate doublets under the assumption that the majority of cells are singlets (Bernstein et al, 2020). Experimental approaches leveraging natural genetic variation, such as SNPs, have been applied in human studies (Kang et al, 2018), but neither strategy is optimized for plant scRNA-seq, where features like endoreduplication add to the overall complexity.

**Figure 5:**
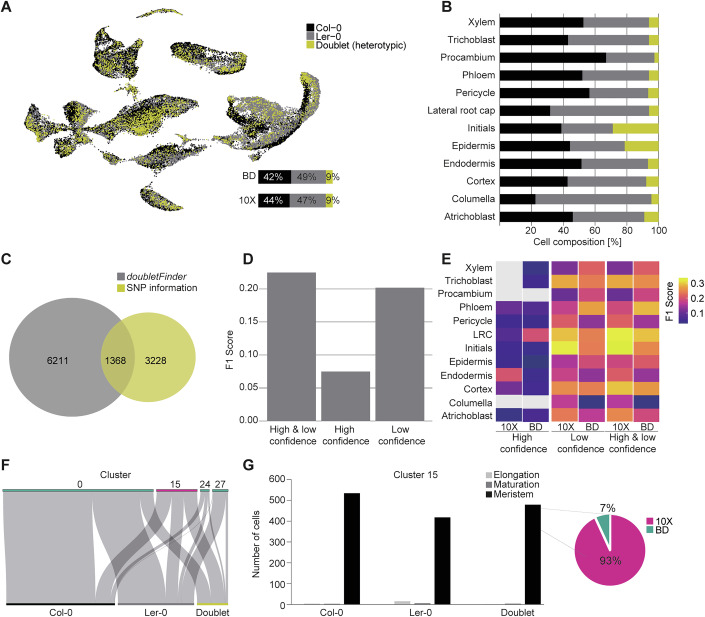
10X Chromium-specific heterotypic doublet cluster with meristematic cells. (**A**) UMAP colored by ecotype (Col-0: black, Ler-0: gray) and heterotypic doublet (yellow) prediction, with relative contributions of each between technologies on the bottom. (**B**) Bar graph quantifies the relative contribution of ecotype and heterotypic doublets to the final cell type composition. (**C**) Venn diagram representing the total number of doublets identified computationally (*DoubletFinder*; gray) and SNP-based (yellow). (**D**) Precision calling of doublets (F1 score) for doublets categorized with high-confidence or low-confidence by *DoubletFinder*, and doublets from both categories combined. (**E**) Heatmap representing F1 score for each cell type, confidence level, and technology. (**F**) Sankey diagram depicting the relationship between ecotype and heterotypic doublets and clusters that exhibit technology-driven cell capture biases. Clusters enriched in cells from BD Rhapsody replicates (0, 24, 27) are indicated in green, while the cluster enriched in 10X Chromium replicates (15) is highlighted in magenta. (**G**) Bar graph showing the number of cells assigned to each developmental stage within cluster 15 (elongation (light gray), maturation (dark gray), and meristem (black)). The pie chart indicates the technology of origin of all meristem-annotated cells in cluster 15. Source data are available online for this figure.

**Figure EV5:**
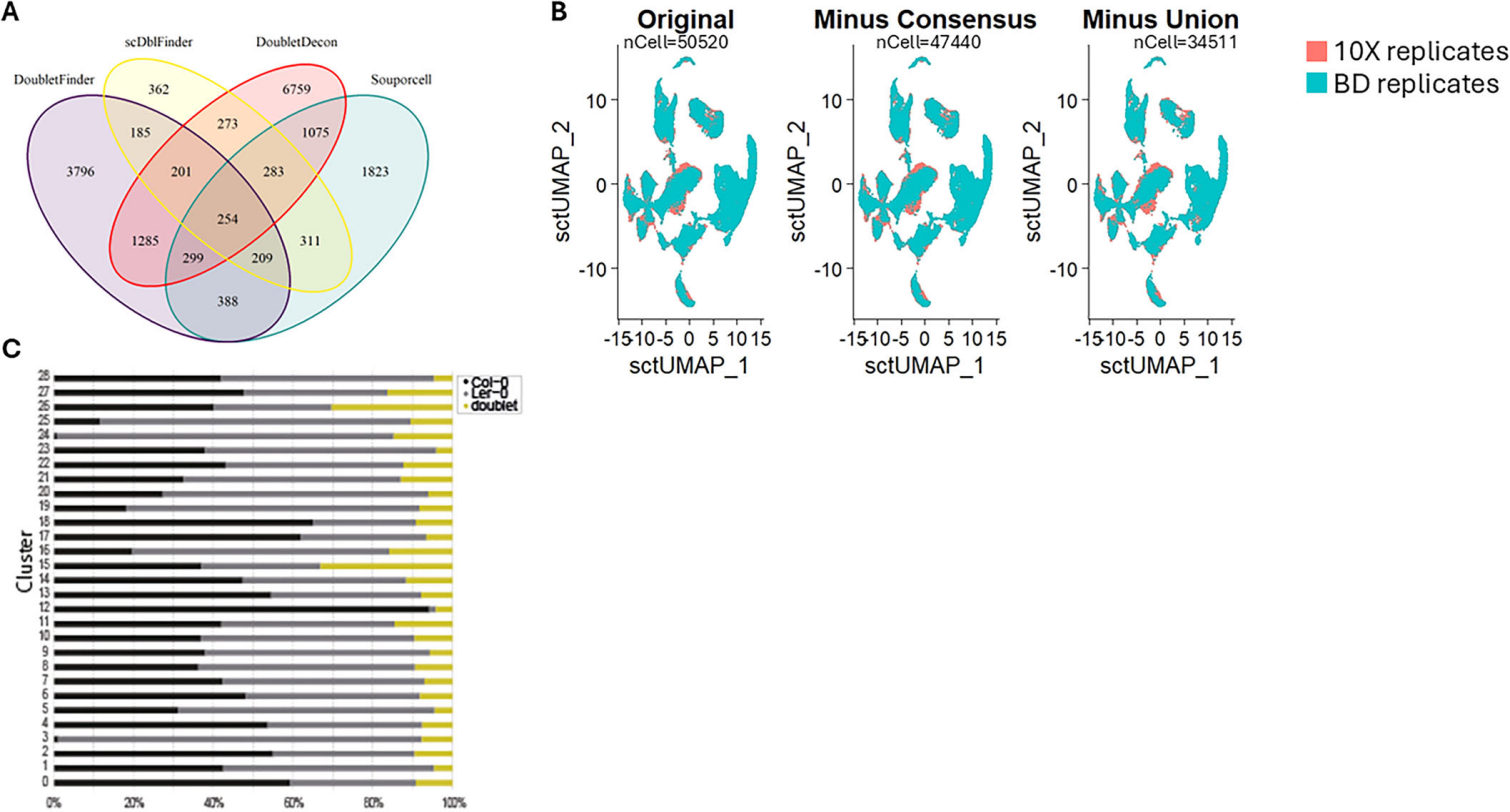
Doublet detection across cell clusters. (**A**) Venn diagram showing overlap and uniquely labeled doublets among *DoubletFinder, scDblFinder, DoubletDecon*, and *Souporcell*. (**B**) UMAP embeddings of the original dataset (*n* = 50,520 cells), after removal of “consensus” doublets (cells called by at least two tools; *n* = 47,440), and after removal of “union” doublets (cells called by at least two tools and confirmed as heterodimeric by SNPs; *n* = 34,511). Cells are colored by platform of origin (10X, orange; BD, blue). (**C**) Stacked bar plots showing, for each cluster (0-27), the proportions of Col-0 (black), Ler-0 (gray), and heterotypic doublets (yellow), with the *x* axis indicating the percentage of cells assigned to each category.

To address this matter, we adopted a SNP-based experimental approach by mixing Arabidopsis Col-0 and Ler-0 ecotypes in equal proportions and processing them on both platforms. We first applied *DoubletFinder* (McGinnis et al, 2019), which simulates synthetic doublets by averaging the gene expression profiles of randomly paired cells, then integrates these artificial doublets into the dataset. A nearest neighbor algorithm assigns doublet scores based on similarity to artificial doublets, with higher scores indicating higher confidence. *DoubletFinder* identified 7579 potential doublets at a simulated 10% doublet rate (Fig. 5A). Incorporation of SNP-based doublet identification revealed an overlap of 1150 cells that were predicted as doublets by *DoubletFinder* and independently confirmed as heterotypic doublets by SNP calling. SNP-identified doublets exhibited a balanced ecotype representation, with 44% Col-0 and 47% Ler-0 in 10x Chromium, and 42% Col-0 and 49% Ler-0 in BD Rhapsody, corresponding to an overall heterotypic doublet rate of 9% across both platforms. All cell types were represented by both ecotypes, confirming robust capture. Doublet rates varied by cell type, with most between 3 and 9%, but epidermis (21%) and initials (29%) showed much higher rates (Fig. 5B). These two cell types also tend to have lower gene and transcript content (Fig. 4D), which may limit the information available for accurate genotype assignment by SNP-based algorithms. Furthermore, the overlap between computationally identified and SNP-confirmed doublets was minimal (Fig. 5C), with only 1150 doublets identified by *DoubletFinder* also detected by the SNP analysis. Using SNP detection in our mixed ecotype experiment as an experimental ground-truth, *DoubletFinder* showed low precision (F1 score = 0.07 for high-confidence doublets), which improved slightly for low-confidence (F1 = 0.20) and combined predictions (F1 = 0.22) (Fig. 5D). At the cell type level, high-confidence doublets alone were insufficient for robust classification, while including low-confidence predictions improved F1 scores, particularly for epidermis, lateral root cap, and procambium (Fig. 5E). The best performance was achieved by combining both confidence levels, with slightly higher precision in BD Rhapsody data. To put these results in context, we also applied three additional doublet-identification tools with distinct underlying strategies: *scDblFinder* (Germain et al, 2021), *DoubletDecon* (DePasquale et al, 2019), and *Souporcell* (Heaton et al, 2020). Across all four methods, 254 droplets were consistently flagged as doublets, while each tool produced substantial numbers of method-specific calls (Fig. EV5; Dataset EV7). When benchmarked against the same SNP-based ground-truth, *scDblFinder* and *DoubletDecon* achieved F1 scores of 0.25, reflecting different trade-offs between precision and recall but similarly modest overall accuracy. We then assessed the impact of doublet removal on downstream analyses by removing either (i) only “consensus” doublets (cells flagged by at least two methods) or (ii) the union of predicted doublets from all tools together with all genotype-confirmed doublets. The consensus strategy removed 3080 cells, whereas the union strategy removed 16,009 cells (32% of all cells; Fig. EV5A,B), yet neither approach substantially altered the overall cluster structure or the platform-associated cluster representation (Dataset EV7). Given the uniformly low F1 scores across all methods and the limited impact of even aggressive doublet removal on downstream results, these analyses indicate that computational doublet identification for plant scRNA-seq data lacks precision and remains challenging.

Finally, we assessed the impact of doublets on cell cluster analysis. Cluster 15, previously noted for platform-associated bias (Fig. 3C), contained 33% heterotypic doublets (Fig. 5F) and was predominantly composed of meristematic cells (93% from 10X Chromium, 7% from BD Rhapsody) (Fig. 5G), indicating a 10X Chromium-specific enrichment of heterotypic doublets among smaller cell types. Other clusters showed 5-16% doublets, except cluster 26 (30%) (Fig. EV5C). This technology-specific bias in doublet capture, particularly for small cells, should be carefully considered in downstream analyses.

## Discussion

This study aimed to assess the strengths and weaknesses of sample preparation protocols, processing platforms, and analysis tools, providing guidance for informed scRNA-seq experimental design in plants. To this end, we compared cell enrichment techniques and evaluated the performance of two widely used high-throughput scRNA-seq platforms, using *Arabidopsis thaliana* root protoplasts (summarized in Table 1). We limited the technology benchmark to two cell sorters, a magnetic levitation assay, and two scRNA-seq platforms: droplet-based (10X Chromium) and microwell-based (BD Rhapsody). While other methods, such as emulsion-based approaches (e.g., PIP-Seq), combinatorial indexing (e.g., SPLiT-seq), or alternative protoplast enrichment strategies (e.g., density gradients; Jo et al, 2025) exist, our goal was to systematically compare platforms that are most commonly employed in plant single-cell studies under standard processing conditions. Our experimental design included three key elements. First, we used a single sample as the input for both technologies, allowing us to clearly attribute any differences in results to the technology itself. Second, the sample came from a heterogeneous but well-characterized plant tissue. This allowed us to compare how each technology performs across diverse cell types with varying cell sizes, cell metabolic and developmental stages, and other individual features that aid its specialized biological function. Third, to accurately detect heterotypic doublets, we included two Arabidopsis ecotypes in the sample. Table 1 summarizes key technical aspects of each platform to guide experimental planning; however, researchers must also consider sample composition, such as cell size heterogeneity, and the specific biological question when making final platform choices.

**Table 1:** Summary of performance evaluation of both platforms.

	10X Chromium	BD Rhapsody
Preconditions for input quality	To prevent chip clogging, samples must have a high purity and should not contain cells larger than 40 µm.	Samples should ideally be free of debris, though contamination will not result in cell or sample loss. There is no need to exclude cells larger than 40 µm, as their presence does not affect the capture or processing of other cells in the sample.
Loading volume	Below 35 µl	320 µl or 600 µl
Sample quality assessment before library prep	Not possible.	Quantification of captured cells and doublet rate.
Gene expression accuracy and potential biases	Reproducible gene capture sensitivity, but an apparent higher mitochondrial read fraction/cell.	Reproducible gene capture sensitivity.
Reproducibility of transcriptional profiles	High reproducibility. Apparent increased gene capture sensitivity for cortex, trichoblast, and pericycle cells.	High reproducibility. Apparent increased gene capture sensitivity for columella, lateral root cap, and xylem cells.
Cell property compatibility	Compatible across all cell types, with apparent increased gene capture sensitivity in trichoblast, pericycle, and cortex cells.	Compatible across all cell types, with an apparent increased capture performance for cell types with lower gene content per cell (e.g., epidermis), larger cells (e.g., lateral root cap cells), and increased plasticity in cell states (e.g., translation activity, stress response).
Doublet formation and detection efficacy	Higher potential of doublet formation from (smaller) meristematic cells.	Doublet identification could, in theory, be enhanced by precise cell counting during sample preparation. However, cell detection by the Rhapsody scanner is not always reliable, which can limit the accuracy of this approach.

Cell sorting and enrichment methods are crucial for obtaining viable, intact cells and removing debris (Box et al, 2020). Although these steps extend the isolation process, they are beneficial because they eliminate debris generated during enzymatic cell wall digestion, which can otherwise negatively impact cell capture and increase ambient noise. Furthermore, the quality of scRNA-seq data is directly related to the number of viable, unstressed cells (Box et al, 2020). While flow cytometry helped in achieving a higher sample purity, magnetic levitation increased the proportion of viable cells and outperformed flow cytometry in maintaining the original size heterogeneity of viable protoplasts (Fig. 1). Given its faster processing time, smaller output volume (ideal for 10X Chromium), and broader tissue compatibility, magnetic enrichment is the more practical choice in case cell type enrichment via FACS is not needed. Based on these results, we recommend incorporating a cell purification step (e.g., magnetic enrichment) into standard protocols. To minimize the detrimental effects of debris and dead cells on data quality, we recommend keeping on evaluating the compatibility of protoplasts with emerging cell enrichment workflows and technologies.

Our comparative analysis of 10X Chromium and BD Rhapsody for plant single-cell transcriptomics revealed apparent platform-associated biases that can implicate data interpretation. Both platforms demonstrated similar efficiency in cell capture and processing, with approximately 60% cell retention (Fig. 2C), which is within the reported cell retention rate for non-plant samples (Gao et al, 2020; Colino-Sanguino et al, 2024; Salcher et al, 2024). However, 10X Chromium had more cells with a higher fraction of mitochondrial reads than BD Rhapsody (Appendix Fig. S2). This contrasts with tumor cell studies, where BD Rhapsody exhibited higher mitochondrial reads, potentially due to enhanced cell lysis (Colino-Sanguino et al, 2024; Salcher et al, 2024). Therefore, we advise testing sample compatibility with the technology’s lysis buffers, potentially with a bulk RNA-seq pilot run to assess cell lysis efficiency, RNA recovery, and representation of known cell types. While both platforms exhibited robust technical reproducibility (Fig. 2I), platform-associated biases in cell cluster composition and cell type representation persisted following batch correction (Figs. 3 and 4). BD Rhapsody-enriched clusters exhibited increased expression of genes associated with translation and abiotic stress responses (Fig. 3G), suggesting a potentially broader capture of cellular heterogeneity and functionally distinct cell states. Consistent with this, BD Rhapsody showed enhanced capture of mature lateral root cap and columella cell states (Figs. 4B and EV4C,D). While multiple factors could contribute to this apparent higher capture rate of mature cells, a key explanation lies in the microwell-based gravity cell compartmentalization employed by Rhapsody, which may favor the capture of larger cells in a mature state compared to 10X’s droplet-based compartmentalization. Both platforms captured all major cell types described in *Arabidopsis* roots, except for quiescent center (QC) cells, which are often underrepresented in scRNA-seq studies due to several factors, including their natural rarity, suboptimal enzymatic digestion conditions, and difficulties in unambiguous identification based on marker genes alone. Furthermore, BD Rhapsody more effectively captured cell types with lower gene content, such as epidermis and initial cells (Fig. 4C). Similar results were reported in benchmark studies with tumor tissues (Salcher et al, 2024; Colino-Sanguino et al, 2024; Hao et al, 2021). The improved capture of low-mRNA content cells by BD Rhapsody, potentially due to its gentler cell separation method, suggests it may be advantageous for studying fragile cells and/or cell types. These findings highlight the importance of considering platform-associated biases when interpreting plant single-cell transcriptomic data, particularly concerning cellular heterogeneity and the representation of specific cell populations.

The observation of a cluster almost exclusively composed of 10X Chromium-derived cells exhibiting a higher proportion of heterotypic doublets, particularly in smaller meristematic cells (Fig. 5F,G), highlights platform-associated bias in doublet formation. Meristematic cells are considered smaller in size, making them more prone to doublet formation with droplet-based platforms like 10X Chromium (Zheng et al, 2017), which emphasizes the importance of considering cell size in experimental design. Importantly, this method also detects heterotypic doublets only. This means that homotypic doublets remain unaccounted for, resulting in a potential underestimation of the true total doublet rate per sample. Furthermore, the detection of heterotypic doublets exposed a significant limitation in current computational doublet identification tools and underscores the potential for misinterpreting gene expression patterns and cell type representation if doublet rates are not accurately accounted for. This is particularly critical, as doublets can generate artificial cell states or mask genuine biological variation, potentially leading to inaccurate conclusions about cell populations and their functions. While the development of plant-specific doublet detection algorithms could help address this issue, the extensive diversity of plant tissues and species analyzed by single-cell transcriptomics makes a universal solution challenging to achieve. Given these technical limitations and the potential for artificial signals generated by undetected doublets, biological validation of the identified gene expression patterns, cell clusters and/or cell states is crucial to avoid misinterpretation of results.

Our benchmark results reveal apparent platform-associated limitations at the cell type level. This information is crucial for optimizing scRNA-seq data resolution and accuracy, tailored to specific experimental designs. However, it also underscores the necessity of avoiding direct comparisons of sample composition and cell type responses across biological conditions when using different platforms. Furthermore, the discrepancy between our SNP-based findings and algorithmic predictions emphasizes the importance of preventing platform overloading, which leads to increased doublet formation and reduced dataset clarity and resolution.

Together, these results provide critical insights for cross-study integration of plant single-cell datasets. While integration methods aim to harmonize data from diverse sources for comparative analysis (Luecken et al, 2022; Zhong et al, 2025), our results highlight that challenges extend beyond bioinformatic correction and originate already at the level of plant-specific sample generation and processing. First, precise documentation of cell isolation and enrichment protocols (e.g., Grones et al, 2024) is essential to evaluate the impact of cell size heterogeneity and cell type capture efficiency across studies. Second, apparent platform-associated biases and technical variation persist even after batch correction, affecting both cell type representation and doublet composition. These biases must be explicitly considered when comparing cell type proportions, biological pathways, or stress-related signatures across studies. To support future efforts, we summarize key considerations for protocol design, data interpretation, and large-scale integration projects in plant single-cell biology in Table 1. While our study focused on root tissue, our primary interest was to assess how cell size heterogeneity and diverse cell-specificities within a complex tissue impact platform performance. We reasoned that these key features (cell size variation, distinct cell states, and functions) are common across plant tissues. Therefore, although we acknowledge that other tissues with unique cell compositions and sensitivities might exhibit different platform compatibility and biases, we believe some of our observations are likely transferable. Future studies using complementary approaches like transcriptome fixation, full-length cDNA protocols, and sample multiplexing would be valuable for a more comprehensive understanding of true cellular heterogeneity across different tissues.

## Methods

**Table:** Reagents and tools table

Reagent/resource	Reference or source	Identifier or catalog number
**Experimental models**		
*Arabidopsis thaliana* Col-0 and Ler-0	Source: De Rybel lab	
*Arabidopsis thaliana* pWER::WER-GFP	Yang et al, 2024	
*Arabidopsis thaliana* pSCR::GFP	Brady et al, 2007	
*Arabidopsis thaliana* pSMB::NLS-GFP	Xuan et al, 2016	
*Arabidopsis thaliana* pCO2::CO2-GFP	Yang et al, 2024	
**Recombinant DNA**		
**Antibodies**		
**Oligonucleotides and other sequence-based reagents**		
**Chemicals, enzymes, and other reagents**		
Mannitol	VWR	25314.264
MES	Duchefa	M1503.0100
CaCl2	Sigma	10035-04-8
KCl	Sigma	7447-40-7
Cellulase	Yakult	L0012
Pectolyase	Kyowa	PECTOLYASE Y23
MS medium	Duchefa	M0221.0050
Propidium iodide	Sigma	P4170
Calcein FM	Invitrogen	C1430
**Software**		
Cellranger (v6.0.0)	10X Genomics	
BD Rhapsody Sequence Analysis Pipeline ‘*Seven bridges’* (v2.0)	BD Biosciences	
STARsolo (v2.7.11a)	https://github.com/alexdobin/STAR	
R software (v4.16)	https://www.r-project.org	
Seurat (v5.0.1)	https://github.com/satijalab/seurat	
Harmony (v0.1.0)	https://github.com/immunogenomics/harmony	
Souporcell (v3)	https://github.com/wheaton5/souporcell	
DoubletFinder (v2.0.6)	https://github.com/chris-mcginnis-ucsf/DoubletFinder	
scDblFinder (v1.20.2)	https://github.com/plger/scDblFinder	
DoubletDecon (v1.1.6)	https://github.com/EDePasquale/DoubletDecon	
LeviCell 1.0	Levitas Bio	PN 1000003
Columbus 9.2.1 /Harmony 5.2	Revvity	
FlowJo v10	BD Biosciences	
BD CellView™ Lens plugin	BD Biosciences	
BD CellView™ Image Extractor	BD Biosciences	
BD DiVa Software 6.1 (BD FACSAria™ II Cell Sorter)	BD Biosciences	
BD FACSChorus 6.1 (BD FACSDiscover™ S8 Cell Sorter)	BD Biosciences	
**Other**		
Chromium Next GEM Single Cell 3ʹ Kit v3.1	10X Genomics	PN-1000268
BD Rhapsody Enhanced Cartridge Reagent Kit	BD Biosciences	664887
BD Rhapsody cDNA Kit	BD Biosciences	633773
BD Rhapsody 8-lane Cartridge	BD Biosciences	666262
BD Rhapsody WTA Amplification Kit	BD Biosciences	633801
BD FACSDiscover™ S8 Cell Sorter	BD Biosciences	
BD FACSAria™ II Cell Sorter	BD Biosciences	
BD FACS Flow Cytometry Sample Tubes	BD Biosciences	352054
BD FACSFlow™ Sheath Fluid	BD Biosciences	342003
BD FACSClean™ Solution	BD Biosciences	340345
BD FACSRinse Solution	BD Biosciences	340346
Dulbecco’s Phosphate Buffered Saline Modified without calcium chloride & magnesium chloride	Sigma-Aldrich	D5652-50L
Illumina NovaSeq 6000	Illumina	
Illumina NextSeq 2000	Illumina	
Illumina NovaSeq S2 flow cell	Illumina	
Illumina NovaSeq S4 flow cell	Illumina	
Illumina NextSeq P3 flow cell	Illumina	
LeviCell S2.3 Cartridges (10 pack)	Levitas Bio	PN 1002010
Levitation Agent (10 rxn)	Levitas Bio	PN 1003001
Opera Phenix	Revvity	
Phenoplate 96	Revvity	6055302
6-well plates	VWR	734-2777
24-well plates	VWR	734-2779

### Plant growth

*Arabidopsis thaliana* ecotypes Columbia-0 (Col-0) and Landsberg erecta-0 (Ler-0) were used for protoplast isolation and single-cell transcriptomics. Quantification of cell size heterogeneity in protoplast suspensions after cell enrichment was performed with Col-0, and p*SCR*::GFP (*AT3G54220*; Brady et al, 2007), p*WER*::WER-GFP (*AT5G14750*; Yang et al, 2024), p*SMB*::NLS-GFP (*AT1G79580*; Xuan et al, 2016), and p*CO2*::CO2-GFP (*AT1G62500*; Yang et al, 2024). For all experiments, seeds were surface-sterilized and germinated on ½MS (Murashige and Skoog, Duchefa) medium after 2 d stratification at 4 °C. Seedlings were grown at 22 °C in continuous light conditions.

### Tissue digestion and single-cell isolation

Root tips (0.5 cm) from 6-day-old Arabidopsis seedlings were used for protoplast isolation, adapted from Wendrich et al (2020). Briefly, root tips were incubated in digestion buffer (0.4 M mannitol, 20 mM KCl, 20 mM MES, 10 mM CaCl_2_, 1.5% Cellulase YC, 0.1% Pectolyase, pH 5.6) for 60 min at room temperature with gentle agitation (30 rpm). The resulting digestion mixture was filtered (70-μm cell strainer), and protoplasts were collected by centrifugation (250 ×* g*, 6 min, room temperature). Protoplasts were then resuspended in 350 μL wash buffer (0.4 M mannitol, 20 mM KCl, 20 mM MES, 10 mM CaCl_2_, pH 5.6).

For single-cell RNA sequencing (scRNA-seq), cell viability was assessed by adding propidium iodide (PI, 14 μM) and calcein AM (15 μM, Thermo Fisher Scientific). Protoplasts were isolated separately for each Arabidopsis ecotype (Col-0 and Ler-0). Viable cells from each ecotype were enriched using magnetic levitation (LeviCell, Levitas Bio; 255 μL cell suspension, 45 μL levitation buffer, 5-min settling time, split line: 15). The enriched cell suspensions (~60 μL per ecotype) were counted, and a master sample containing 40,000 cells (50:50 Col-0:Ler-0 mix) was prepared.

This master sample was divided and processed using two scRNA-seq platforms: 10X Chromium (Single Cell 3′ GEM, Library & Gel Bead Kit v3.1) and BD Rhapsody (HT cartridge, enhanced cartridge reagents v2, cDNA and WTA amplification kit), following the manufacturers’ protocols for reverse transcription, cDNA amplification, and library construction. Libraries were sequenced on an Illumina NovaSeq 6000 or NextSeq 2000 instrument at the VIB Nucleomics Core (Leuven). The single-cell experiment was independently replicated (*n* = 2). No blinding was performed during data collection or analysis.

### Quantification of cell size heterogeneity after cell enrichment

Protoplasts from *Arabidopsis thaliana* Col-0 roots were isolated as described above. After the addition of 14 μM PI and 15 μM calcein AM, the protoplast mixture was divided into four aliquots for downstream analysis: (1) an unsorted control, (2) flow cytometry sorting using a BD Aria II, (3) image-based flow cytometry using a BD FACSDiscover^TM^ S8, and (4) magnetic levitation-based enrichment using the LevitasBio LeviCell platform. For morphology analysis of cell type marker lines, only PI was added, as calcein AM fluorescence overlaps with GFP signals.

For each sorting or enrichment method, 100,000 events (protoplasts) were collected. Following magnetic levitation, protoplasts from both the enriched and unsorted samples were counted using a hemocytometer to determine cell concentration. Each sample was then diluted to achieve an equivalent concentration of 100,000 protoplasts per 100 μL, and three technical replicates were prepared at this concentration. For flow cytometry, protoplasts were sorted directly into 12 wells, each containing 100 μL. The data shown in Fig. 1 summarize two biologically independent experiments.

For all samples, cell concentration, viability (pp_pi_pp_2; 1 = viable, 0 = non-viable), and size distribution were subsequently analyzed based on images acquired using an Opera Phenix High Content Imaging instrument (Revvity) (Dataset EV2; Dataset EV6). No blinding was performed during data collection or analysis. Brightfield, Digital Phase Contrast, calcein AM, and PI images were acquired as an 8-plane Z-stack with a 10X objective in confocal mode. To cover ~80% of the well, 9 FOV per well were required. Images were transferred to Columbus (Revvity) for analysis. Using texture analysis on the BF channel and cell detection on the DPC channel, protoplasts were identified and segmented based on roundness and size feature thresholds. Calcein AM and PI intensity features were used to identify alive (=PI-negative and calcein-positive) and the total number of protoplasts.

Cell diameter (µm) was calculated as the square root of (cell length ×  cell width). Mean cell diameter and roundness were calculated separately for viable and non-viable cells. Global relationships between size and shape were visualized by plotting cell diameter versus roundness, and linear regression coefficients were determined. Diameter distributions were visualized using violin, box, and ridge plots (*ggplot2*). To assess enrichment of large cells, viable cells with a diameter >20 µm were quantified, and their proportion per purification method was displayed as bar plots.

### Raw data processing, cell quality filtering, and scRNA-seq analysis

#### Mapping

Raw scRNA-seq data were initially processed using the technology-specific pipelines: *Cell Ranger* 6.0.0 (10X Genomics) and *Seven Bridges* (BD, v.2.0). These pipelines performed demultiplexing and alignment of reads to the TAIR10 Arabidopsis genome. Sequencing reads were randomly subsetted to 150,304,927 reads for each library.

The reference genome was compiled with *STAR* v2.7.11a using the TAIR10 (ENSEMBL release 57) genome fasta file and Araport11 GTF annotation file. *STARsolo* (*STAR* v2.7.11a) was used to generate count matrices for all samples. For the 10X Genomics samples, the files were processed using a whitelist of cell barcodes provided by 10X Genomics, with additional specification of a 12 bp UMI length and 16 bp cell barcode length. For the BD Rhapsody samples, files were processed using whitelists of the three cell barcode sections provided by BD, with specification of a 8 bp UMI length and three 9 bp cell barcode sections separated by two linkers of 4 bp. For all samples, reads mapping to either exons or introns were counted.

A detailed overview of sample origin, preparation, and analysis for the generated scRNA-Seq samples is provided in Dataset EV8.

#### Quality control and cell filtering

Subsequent processing and quality control were conducted using *Seurat* (v.5.0.1) in R (v.4.16) (Hao et al, 2021, 2024). Genes expressed in fewer than three cells were removed. Cells with fewer than 300 detected genes or 800 unique molecular identifiers (UMIs) were excluded from further analysis. Further cell filtering included the exclusion of cells with more than 10% mitochondrial reads and 5% of chloroplast reads. These filtering criteria are in line with commonly used QC thresholds for plant single-cell transcriptomics datasets (e.g., Grones et al, 2024).

#### Normalization, clustering, and differential expression analysis

Normalization, identification of highly variable genes (HVGs), and scaling were done through the SCTransform function of *Seurat*, followed by clustering and dimensionality reduction. Principal component analysis (PCA) was performed using the top 3000 highly variable genes. After processing each sample separately, the four objects were merged, and SCTransform was run again on the merged object, leading to a list of 3000 HVGs across all cells from the four samples. Expression values were averaged across all cells within each sample. Pearson correlation coefficients were then calculated between replicates using these averaged HVG expression profiles. Only genes with non-zero expression in at least one replicate were included, and cells included in the averages had passed standard QC thresholds (minimum counts, feature counts, and mitochondrial content filters). Differential gene expression was calculated with the FindMarkers (test.use = ”wilcox”, logfc.threshold = 1, only pos = T, min.pct = 0.1) function in *Seurat*. Top 5 and top 100 marker gene lists were created for each cluster and used for gene expression consistency testing between replicates and platforms, and GO term analysis, respectively.

#### Cluster expression analysis

For each cluster, average expression and the number of expressing cells were calculated for each of the top 5 marker genes across the four samples (Dataset EV5). Within each cluster, Pearson correlation coefficients were calculated from averaged expression values for (i) within-platform correlation between replicates of the same technology, and (ii) a cross-platform correlation, comparing the mean of the two 10X replicates to the mean of the two BD replicates. Missing values were handled using pairwise complete observations so that only genes quantified in at least one of the paired profiles contributed to the correlation estimates. Percentages of expressing cells were calculated as the fraction of cells with non-zero expression per gene.

#### Sequencing-depth subsampling and drop-out analysis

For the sequencing-depth subsampling analysis, raw sequencing reads were randomly downsampled to 50, 100, and 150 M reads per sample, and *Seurat* objects were regenerated using the same preprocessing. At each subsampled depth, captured genes and UMIs per cell were quantified. Gene counts and fraction of cells with non-zero gene count were calculated and presented as per gene detection fractions, providing for each gene the percentage of cells where it is detected, categorized by sequencing depth and platform. Pearson and Spearman correlation coefficients were calculated between platforms at each depth to quantify concordance in detection rates.

#### Analysis of platform-associated genes

Platform-associated candidate marker genes (Dataset EV4) were compiled based on differential gene count detection across platforms. First, filtering was done by selecting for genes with detected counts in one replicate and complete absence in the other platform in the corresponding replicate (e.g., 10X_rep1 markers had >0 detected cells in 10X replicate 1 and 0 detected cells in BD replicate 1; BD_rep2 markers had >0 detected cells in BD replicate 2 and 0 detected cells in 10X replicate 2).

Second, gene lists were then required to be consistently detected in both biological replicates of one platform and completely undetected in both replicates of the other platform (10X_specific: >0 detected cells in 10X_rep1 and 10X_rep2, 0 detected cells in BD_rep1 and BD_rep2; BD_specific: >0 detected cells in BD_rep1 and BD_rep2, 0 detected cells in 10X_rep1 and 10X_rep2).

Third, to analyze the occurrence of marker expression across the replicates, raw RNA counts were extracted for both marker lists and genes retained if they were detected (non-zero counts) in at least 25 cells, providing a cell count that expresses 10X-specific or BD-specific marker subsets.

#### Gene ontology (GO) enrichment analysis

GO enrichment analysis was performed using the PANTHER overrepresentation test with Fisher’s exact test, and *P* values were corrected for multiple testing using the Benjamini–Hochberg false discovery rate (FDR). GO terms were ranked by adjusted *P* value and visualized as −log₁₀(*P*.adjust).

#### Data integration

Data integration across replicates was performed using *Harmony* (v0.1.0; Korsunsky et al, 2019). The integrated dataset was then subjected to dimensionality reduction with UMAP (RunUMAP, reduction = ‘harmony’, dims = 1:30), neighbor graph construction (FindNeighbors, reduction = ‘harmony’, dims = 1:30), and clustering (FindClusters, resolution = 0.8) to identify distinct cell populations. Next to *Harmony*, also *FastMNN* (Haghverdi et al, 2018 Nat Biotech) and *CCA* (Butler et al, 2018 Nat Biotech) were used to integrate the datasets through the *Seurat* IntegrateLayers function. As a reference, the samples were also merged in *Seurat* without any batch correction method. UMAP plots were generated, and clusters were labeled either according to the cluster annotation provided by each integration method or using the previously obtained cluster annotation from *Harmony*. The origin information of each cell (from which platform and replicate they were obtained) was extracted, and the cluster composition (according to *Harmony*-assigned cluster ID) was plotted as a stacked bar graph for each integration method.

#### Cell type annotation

Cell types, cell states, and cell cycle phases were assigned by label transfer using published root meristem atlases (Wendrich et al, 2020; Shahan et al, 2022). For confidence visualization, cells were grouped by predicted annotation (“annotation.predicted”) and replicate, and for each group, the number of cells, median and mean annotation score, and the interquartile range (IQR) of the “annotation.predicted.score” were calculated and visualized.

#### Gene content classification

To assess gene content variation across major Arabidopsis root cell types, we inspected the distribution of detected genes (nFeature_RNA). For cortex, trichoblast, lateral root cap, and columella, we observed a bimodal pattern. For these cell types, we defined a cell type-specific threshold at the local minimum between modes, yielding cut-offs of >2000 detected genes for cortex, >3000 for trichoblast, >1500 for lateral root cap, and >2000 for columella. Cells above the threshold were classified as “High” gene content, and cells below as “Low”. To evaluate the influence of sequencing technology on gene detection, cells classified as “High” were further assigned to “10X_High” or “BD_High” based on their technology of origin; all remaining cells were labeled “Low”. The distribution of these categories was visualized using UMAP dimensionality reduction, with cells colored according to both gene content and technology group.

#### Cell genotyping and computational doublet identification

*Souporcell* (Heaton et al, 2020) was used to assign cells to genetic backgrounds, specifying two genotype clusters and using a VCF file containing Col-0 and Ler-0 SNPs (1001 Genomes). The output of *Souporcell* is a classification whether the cell has SNPs corresponding to the Col-0 genotype, the Ler-0 genotype of a mixture of the two (“doublet”).

*DoubletFinder* (McGinnis et al, 2019) was used to label doublets from each Seurat object obtained after cell filtering, with the parameters varying from an estimated 1 to 10% doublets. In addition, also *scDblFinder* (Germain et al, 2021) and *DoubletDecon* (DePasquale et al, 2019) were used for benchmarking purposes. *DoubletFinder* was run on the separate samples, where each cell was assigned a doublet prediction status: “High Confidence”, “High Confidence adjusted”, “Low Confidence”, “Low Confidence adjusted”, or “Singlet”. For downstream evaluation, all “High Confidence,” “High Confidence adjusted,” “Low Confidence,” and “Low Confidence adjusted” predictions were grouped as “doublet”, while “Singlet” predictions were classified as “singlet.” *scDblFinder* was run on the merged object with the “samples” option to predict doublets for each sample separately, using the annotated cell types as clusters. *DoubletDecon* was run on the separate samples based on the protocol published in *STAR* Protocols (DePasquale et al, 2019). A rho value of 1.4 yielded the best results in merging highly similar clusters. The output of both *scDblFinder* and *DoubletDecon* was the classification of each cell as either “singlet” or “doublet”.

#### Doublet prediction performance evaluation

For *DoubletFinder* three prediction strategies were evaluated, “high only” (only cells with “High Confidence” or “High Confidence adjusted” predictions were considered doublets), “high + low” (cells with either high or low confidence predictions were considered doublets), and “low only” (only cells with “Low Confidence” or “Low Confidence adjusted” predictions were considered doublets). For each strategy, precision, recall, and F1 score were calculated. For this analysis, we defined (i) True Positives (TP) = cells predicted as doublets by *DoubletFinder* and confirmed as true doublets by the SNP-based ground-truth; (ii) False Positives (FP) = cells predicted as doublets by *DoubletFinder* but identified as singlets in the SNP-based approach; (iii) False Negatives (FN) = cells predicted as singlets by *DoubletFinder*, but identified as doublets in the SNP-based approach. Precision, representing the proportion of predicted doublets that were true doublets, was calculated by using TP/(TP + FP). Recall, defined as the proportion of true doublets that were correctly predicted, was calculated using TP/(TP + FN). The F1 score, defined as the harmonic mean of precision and recall, was calculated as the product of precision and recall, divided by the sum of precision and recall. The numerator is multiplied by two to obtain the final F1 score.

To benchmark computational doublet identifiers, SNP-based calls were converted into a binary ground-truth label (true_doublet; 1 = doublet, 0 = singlet), and predictions from *DoubletFinder* (using the “high + low” strategy), *scDblFinder*, and *DoubletDecon* were harmonized into binary form (1 = predicted doublet, 0 = predicted singlet). Precision, recall, and F1 scores were computed for each method, and contingency tables were generated for selected clusters to inspect method-specific patterns of false positives and false negatives relative to the SNP ground-truth (Dataset EV7). To define robust doublet sets for downstream analyses, cells flagged as doublets by at least two of the three computational approaches were classified as “consensus” doublets, while cells flagged by at least one method (SNP-based or computational) were classified as “union” doublets. Two filtered Seurat objects were created by removing either consensus doublets or all union doublets, and UMAPs colored by platform (10X vs BD) were used to compare overall cluster structure and platform mixing in the original versus filtered datasets.

## Supplementary information

Appendix

Peer Review File

Dataset EV1

Dataset EV2

Dataset EV3

Dataset EV4

Dataset EV5

Dataset EV6

Dataset EV7

Dataset EV8

Source data Fig. 1

Source data Fig. 2

Source data Fig. 3

Source data Fig. 4

Source data Fig. 5

Expanded View Figures

## Data Availability

The datasets and computer code produced in this study are available in the following databases: scRNA-seq data: NCBI under GEO number GSE300264, merged scRNA-seq data: http://www.single-cell.be/plants. The source data of this paper are collected in the following database record: biostudies:S-SCDT-10_1038-S44318-026-00800-5.
