# Supplementary material for: Benchmarking plant single cell RNA-sequencing sample processing strategies
Source: EMBO J. 2026 May 9;45(12):4337–59. doi: 10.1038/s44318-026-00800-5 (PMC13270049; doi:10.1038/s44318-026-00800-5)
Supplement: Supplementary file 1 — Appendix [file 44318_2026_800_MOESM1_ESM.pdf]

Appendix for

Benchmarking plant single cell RNA-sequencing sample processing strategies

Table of content **Page**

Appendix Figure	Page
Appendix Figure S1: Effect of sequencing subsampling on cell content metrics.	2
Appendix Figure S2: Optimization of cell filter pipelines for plant scRNA-seq data.	3
Appendix Figure S3: Unique detection of apparent cluster-specific marker genes in 10X and BD samples persists across batch-correction methods.	4
Appendix Figure S4: Cell diameter distribution in cortex, endodermis, epidermis and lateral root cap (LRC) cells.	5

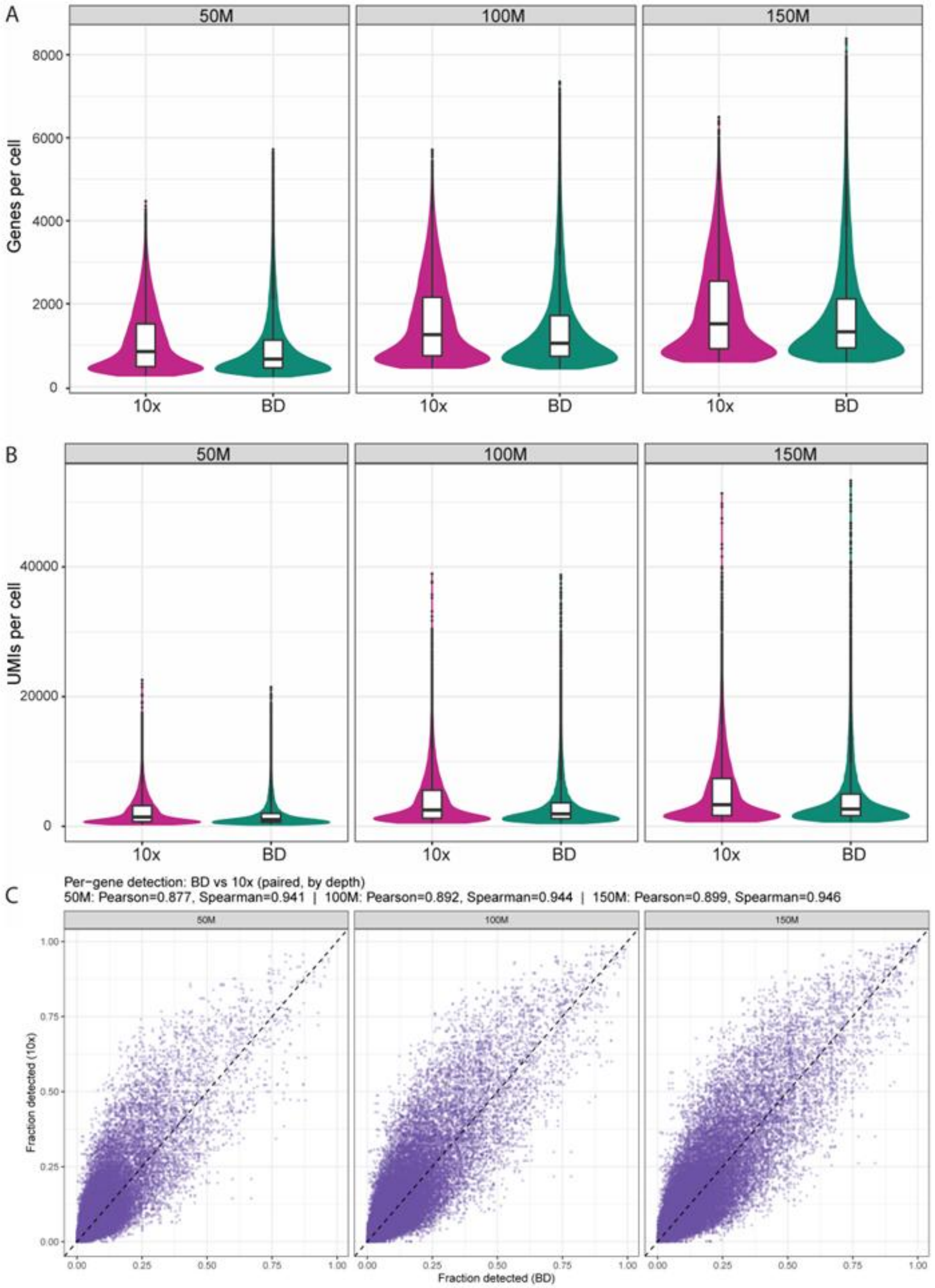

Appendix Figure S1: Effect of sequencing subsampling on cell content metrics.

A) Violin plots showing the distribution and mean of genes per cell in 10X10X (magenta) and BD (green) samples after subsampling each library to 50M, 100M, and 150M reads. **B)** Violin plots showing the distribution and mean of UMIs per cell at the same subsampled depths. **C)** Scatter plots of per-gene detection frequencies in 10X (Y-axis) versus BD (X-axis) for each subsampled depth, with Pearson and Spearman correlation coefficients reported above each panel.

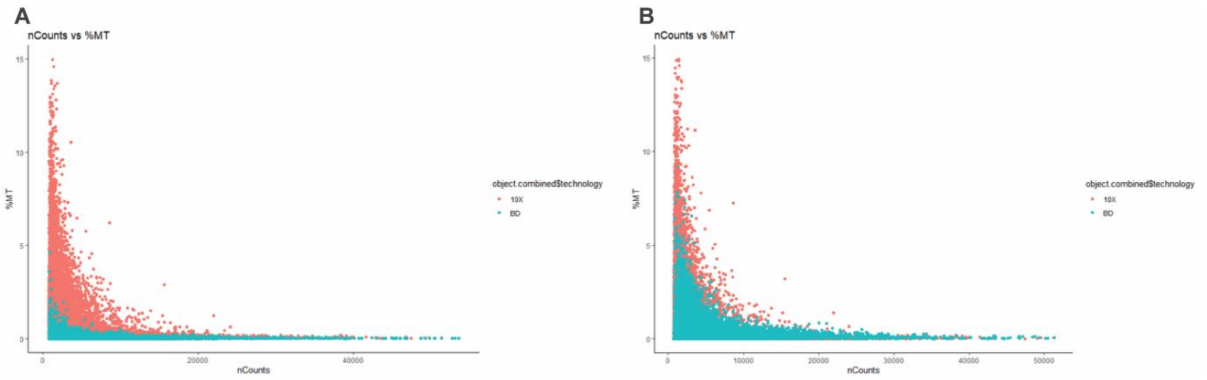

Appendix Figure S2: Optimization of cell filter pipelines for plant scRNA-seq data. Correlation between number of genes ("nCounts") to percentage of mitochondrial reads. **A)** Data was analyzed using the processing pipelines provided by the technology provider (*Cell Ranger* for 10X Chromium samples (red), and *Seven Bridges* for BD Rhapsody samples (blue). **B)** Data was analyzed with a customized pipeline.

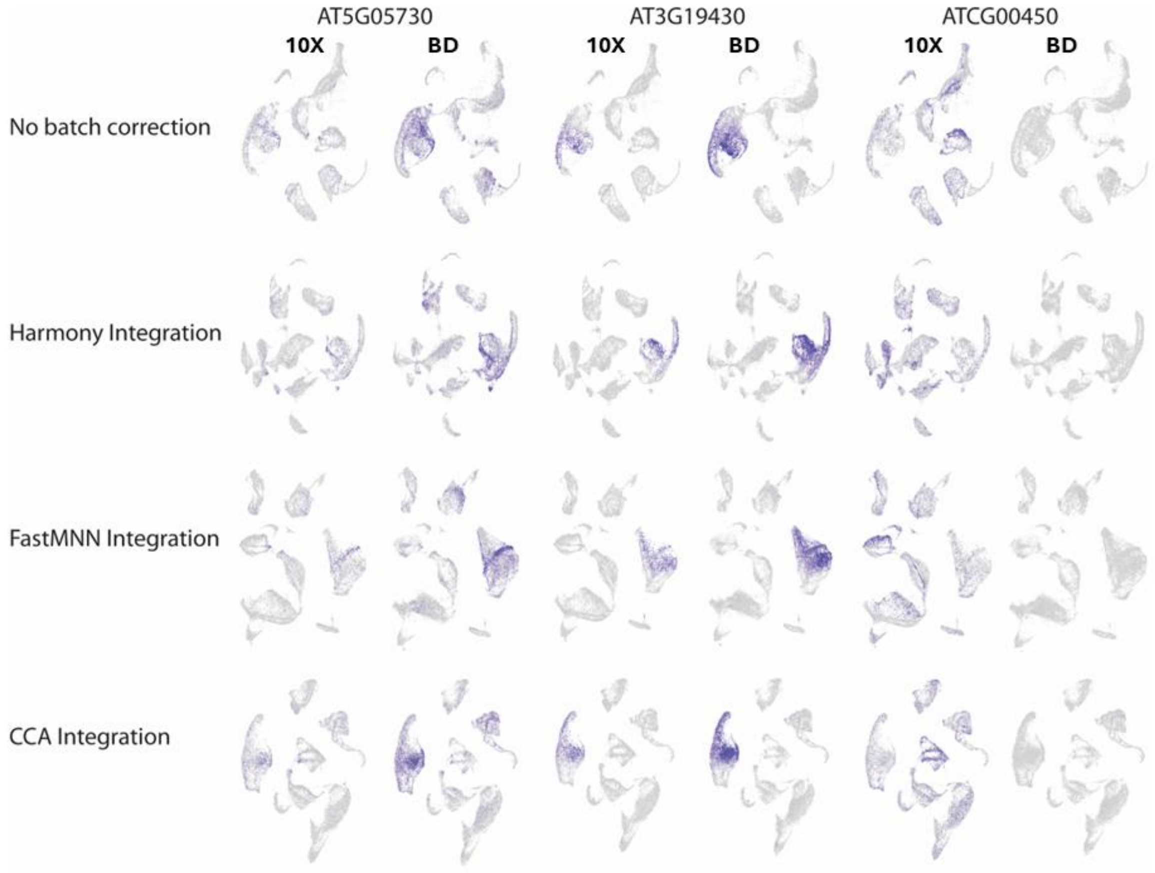

Appendix Figure S3: Unique detection of apparent cluster-specific marker genes in 10X and BD samples persists across batch-correction methods.

Feature plots on UMAP embeddings for 10X (left panels) and BD (right panels) samples generated without batch correction, after *Harmony* integration, after *FastMNN*, and after *CCA* integration, illustrating that platform-specific detection of selected cluster-specific marker genes is maintained under all four processing workflows**.**

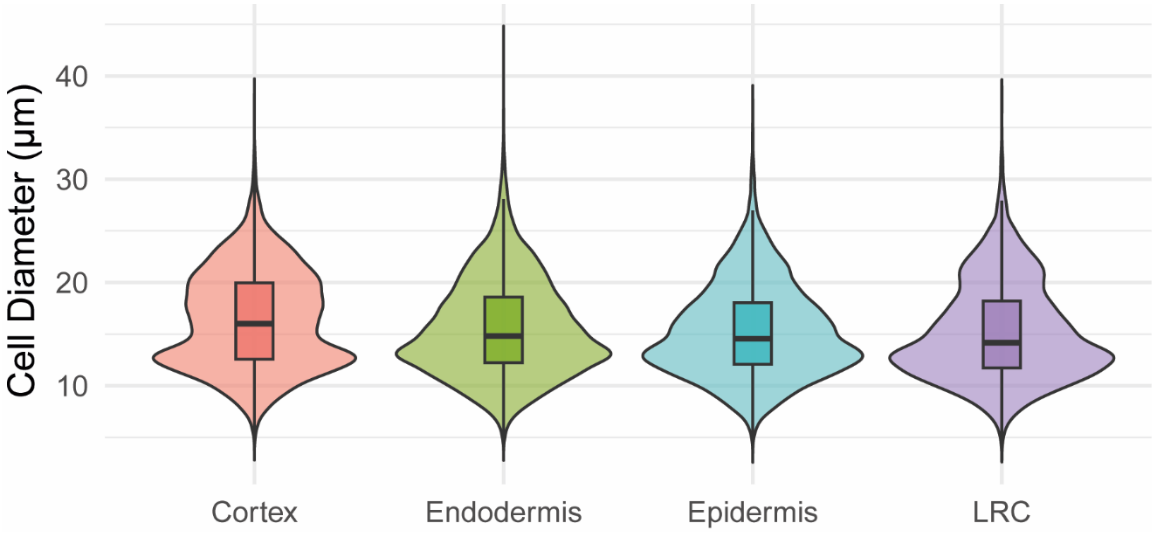

Appendix Figure S4: Cell diameter distribution in cortex, endodermis, epidermis and lateral root cap (LRC) cells.

Violin plots showing the distribution and mean of cell diameters for cells from cortex (p*CO2*::CO2-GFP), endodermis (p*SCR*::GFP), epidermis (p*WER*::WER-GFP), and lateral root cap (p*SMB*::NLS-GFP) marker lines. Data represents one biological replicate, each performed in technical triplicates.
