# Supplementary figures and images for: Benchmarking plant single cell RNA-sequencing sample processing strategies
Source: EMBO J. 2026 May 9;45(12):4337–59. doi: 10.1038/s44318-026-00800-5 (PMC13270049; doi:10.1038/s44318-026-00800-5)

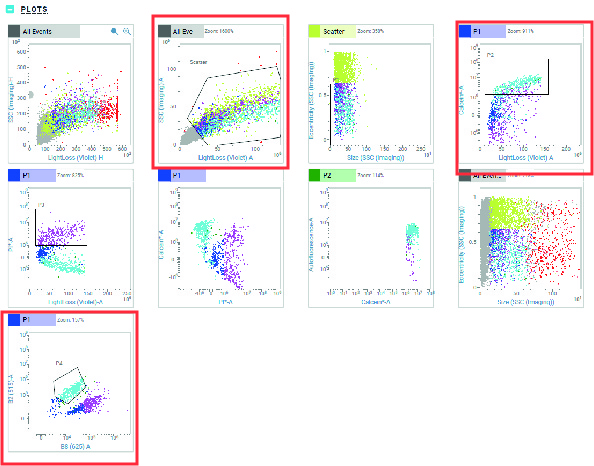

Supplement: Supplementary file 11 — Source data Fig. 1 [file 44318_2026_800_MOESM11_ESM.zip › Figure 1/Panel A/S8_output.jpg]

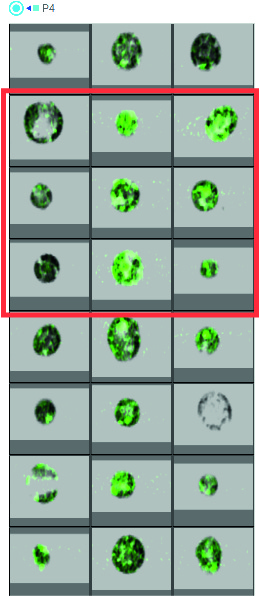

Supplement: Supplementary file 11 — Source data Fig. 1 [file 44318_2026_800_MOESM11_ESM.zip › Figure 1/Panel A/S8_output_AliveCells.jpg]

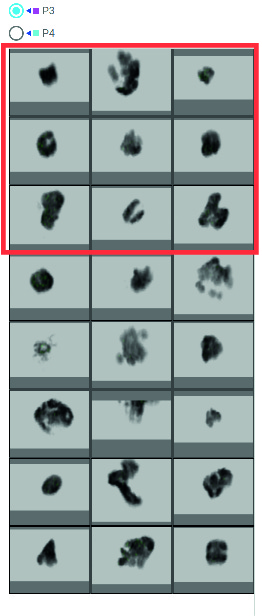

Supplement: Supplementary file 11 — Source data Fig. 1 [file 44318_2026_800_MOESM11_ESM.zip › Figure 1/Panel A/S8_output_DeadCells.jpg]

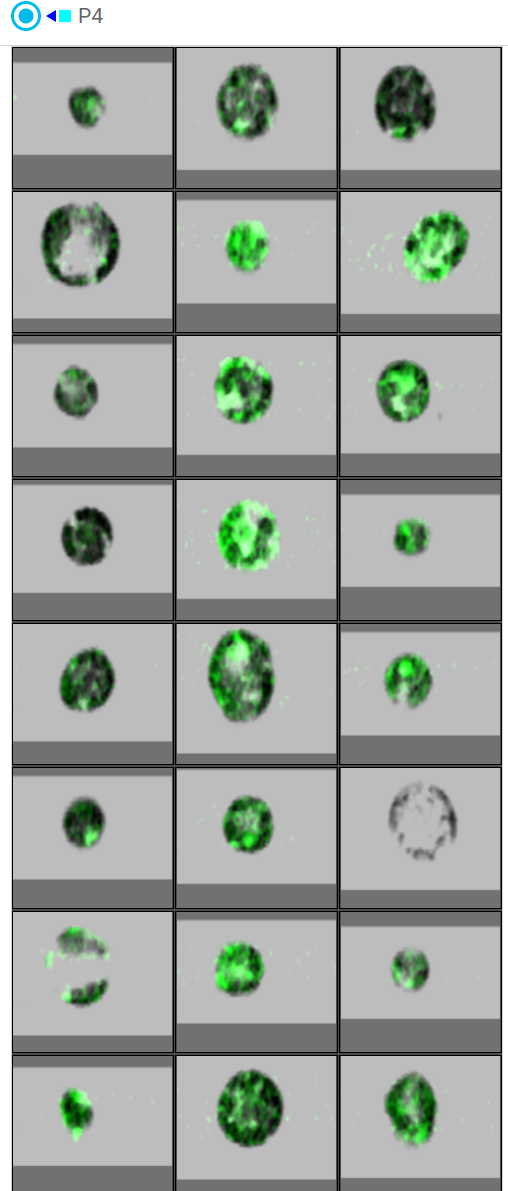

Supplement: Supplementary file 11 — Source data Fig. 1 [file 44318_2026_800_MOESM11_ESM.zip › Figure 1/Panel A/Snapshot LeafProtoplasts_PI_Calcein_Calceinpos2.PNG]

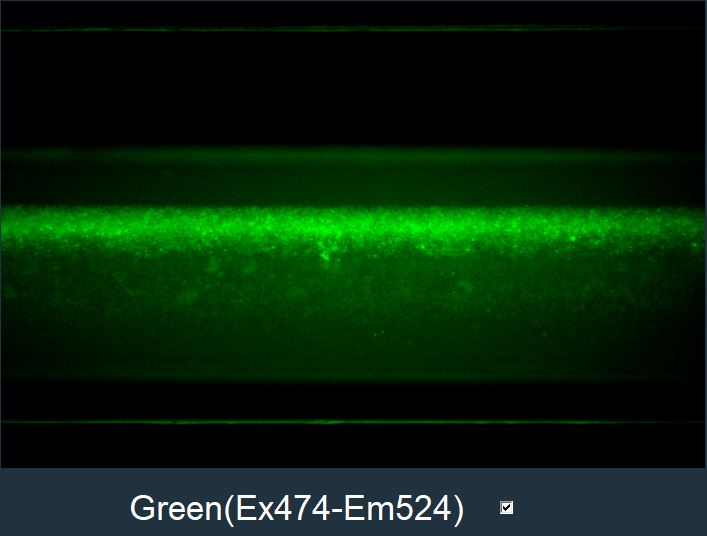

Supplement: Supplementary file 11 — Source data Fig. 1 [file 44318_2026_800_MOESM11_ESM.zip › Figure 1/Panel B/Levitas_Green.JPG]

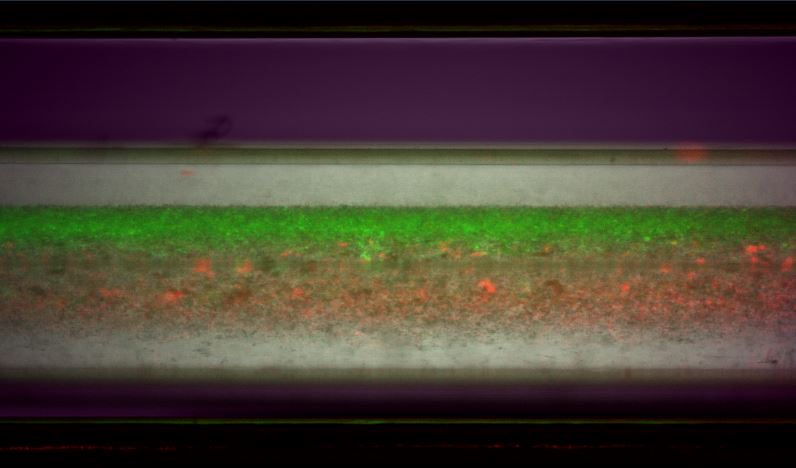

Supplement: Supplementary file 11 — Source data Fig. 1 [file 44318_2026_800_MOESM11_ESM.zip › Figure 1/Panel B/Levitas_greenred.JPG]

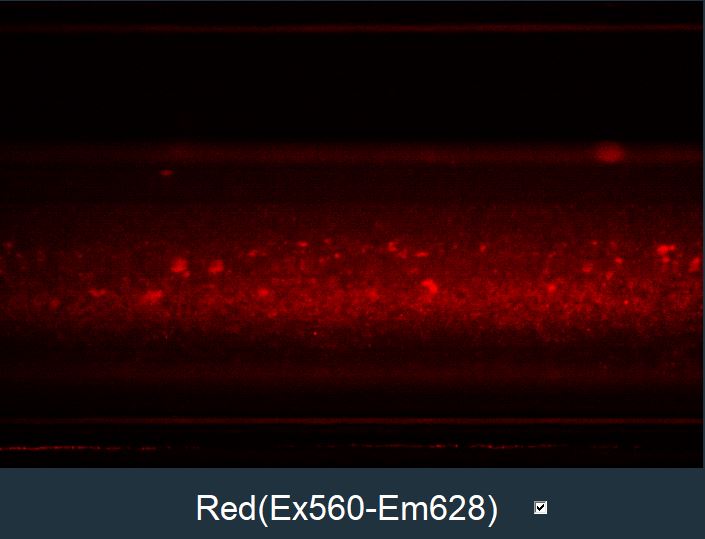

Supplement: Supplementary file 11 — Source data Fig. 1 [file 44318_2026_800_MOESM11_ESM.zip › Figure 1/Panel B/Levitas_Red.JPG]

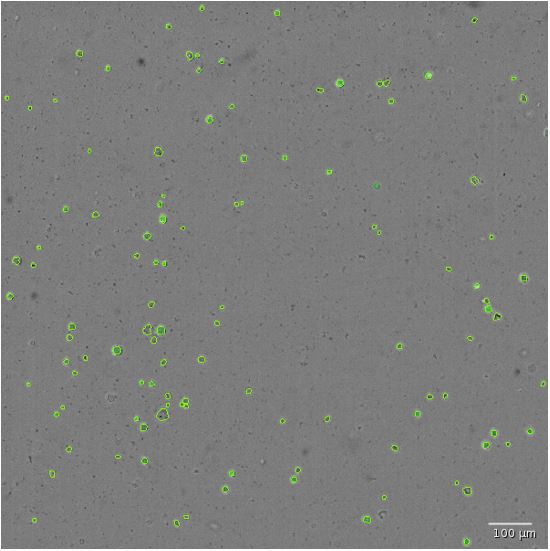

Supplement: Supplementary file 11 — Source data Fig. 1 [file 44318_2026_800_MOESM11_ESM.zip › Figure 1/Panel C/Aria_BF_Calcein_PI_opera.JPG]

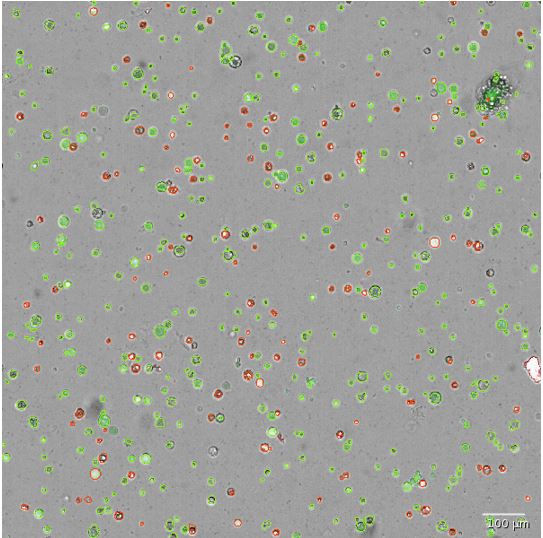

Supplement: Supplementary file 11 — Source data Fig. 1 [file 44318_2026_800_MOESM11_ESM.zip › Figure 1/Panel C/Levitas_BF_Calcein_PI_opera.JPG]

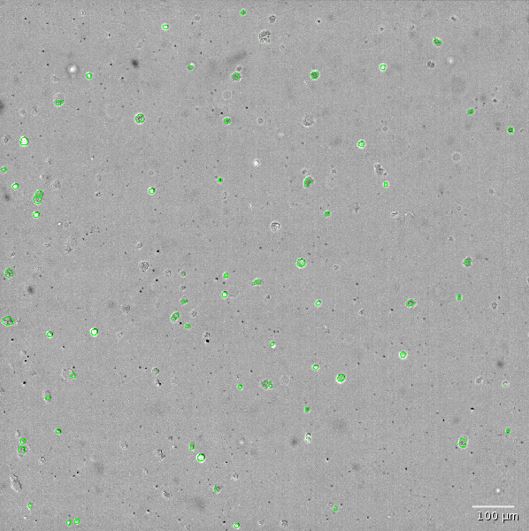

Supplement: Supplementary file 11 — Source data Fig. 1 [file 44318_2026_800_MOESM11_ESM.zip › Figure 1/Panel C/S8_BF_Calcein_PI_opera.JPG]

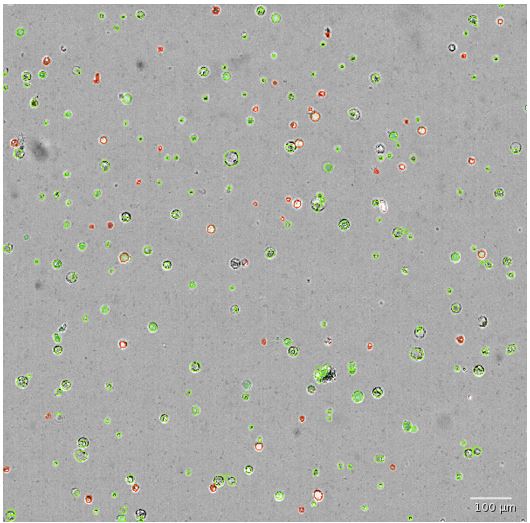

Supplement: Supplementary file 11 — Source data Fig. 1 [file 44318_2026_800_MOESM11_ESM.zip › Figure 1/Panel C/Unsorted_BF_Calcein_PI_opera.JPG]
