# Supplementary material for: Benchmarking plant single cell RNA-sequencing sample processing strategies
Source: EMBO J. 2026 May 9;45(12):4337–59. doi: 10.1038/s44318-026-00800-5 (PMC13270049; doi:10.1038/s44318-026-00800-5)
Supplement: Supplementary file 16 — Expanded View Figures [file 44318_2026_800_MOESM16_ESM.pdf]

Expanded View Figures

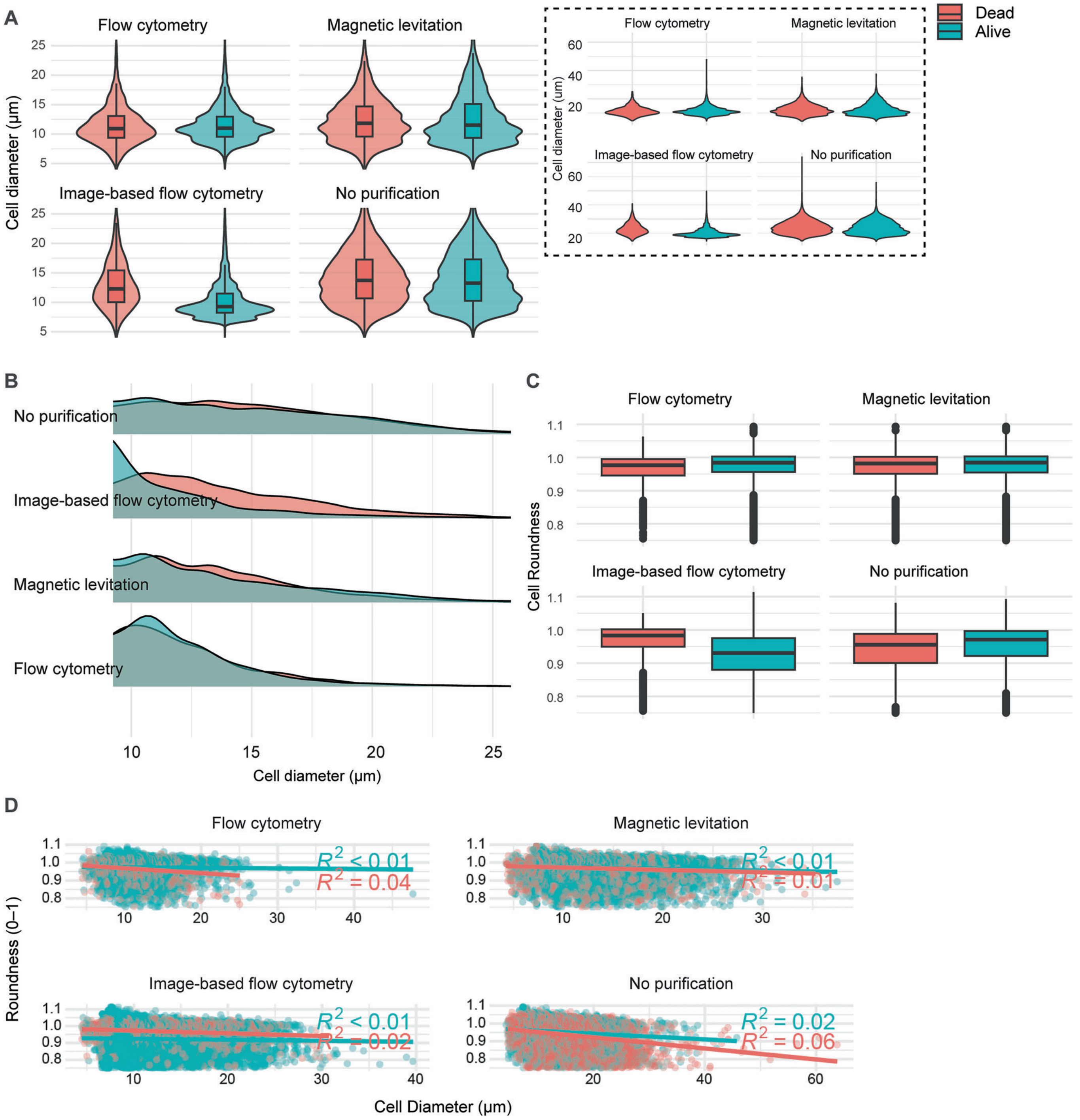

Figure EV1. Cell diameter and roundness in viable and non-viable cells after purification.

(**A**) Violin plots showing the distribution and mean of cell diameters for viable (calcein-positive, turquois) and non-viable (PI-positive, orange) cells after image-based flow cytometry, magnetic levitation, or without purification. Embedded boxplots indicate the median (central line, 50th percentile), with the box spanning the 25th (Q1) to 75th (Q3) percentiles, representing the interquartile range (IQR) and the middle 50% of the data. Whiskers extend to the most extreme values within 1.5 × IQR from Q1 and Q3. The inset on the right shows the full diameter range. (**B**) Density plots of the same diameter measurements as in A), highlighting differences in the frequency of specific size ranges. (**C**) Box plots with the mean of cell roundness for viable and non-viable cells after purification and in the unsorted sample. The central line represents the median (50th percentile), the box spans the 25th (Q1) to 75th (Q3) percentiles, encompassing the middle 50% of the data. Whiskers extend to the most extreme values within 1.5 × IQR from Q1 and Q3. Individual points beyond the whiskers are plotted as dots to indicate outliers. (**D**) Scatter plots of cell roundness versus diameter for viable and non-viable cells under each condition. The low r^2 values indicate no appreciable correlation between cell size and roundness in any condition. Data represent two independent biological replicates, each performed in technical triplicates.

 © The Author(s)

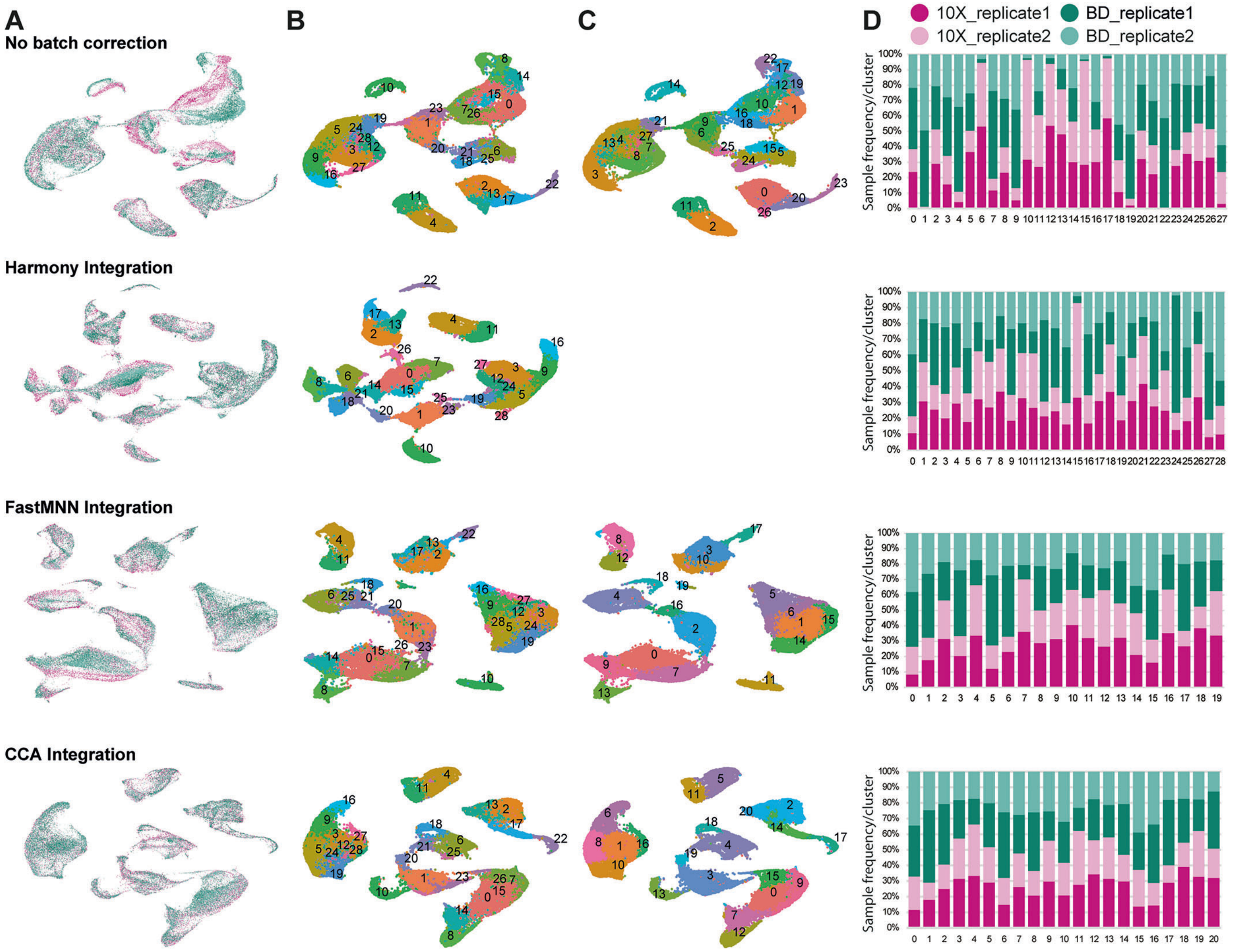

Figure EV2. Cluster composition after batch correction.

(**A**) UMAPs colored by platform of origin (10X, magenta; BD, green) for data processed without batch correction and after *Harmony*, *FastMNN*, or *CCA* integration. (**B**) The same UMAPs as in (**A**), colored by clusters defined within each respective integration workflow. (**C**) The same UMAPs as in (**A**), but with cluster labels transferred from the *Harmony*-derived clustering. (**D**) Bar plots showing, for each clustering scheme, the proportion of cells in each cluster originating from 10X (magenta) or BD (green).

© The Author(s)

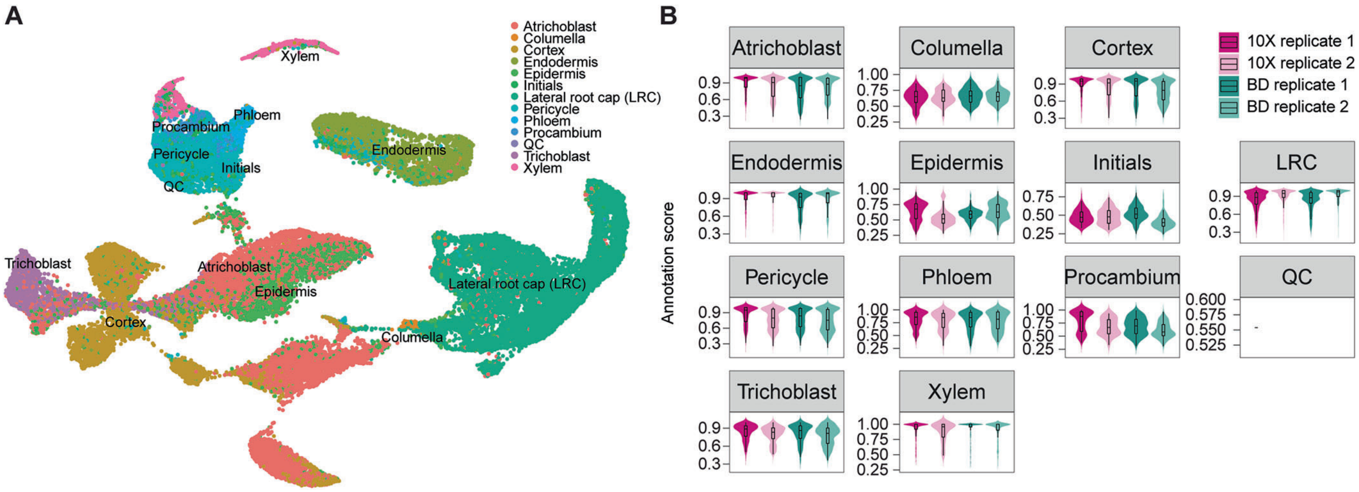

Figure EV3. Cell type annotation.

(**A**) UMAP visualization of the integrated dataset following batch effect correction, colored by annotated cell types. (**B**) Violin plots displaying prediction scores grouped by cell type. Cell origin is indicated by color: 10X Chromium replicates (magenta) and BD Rhapsody replicates (green). The central line in each boxplot represents the median (50th percentile), the box spans the interquartile range (25th-75th percentile), and whiskers extend to the most extreme values within 1.5 × the interquartile range (IQR). Data are from $n = 2$ biological replicates, with distributions showing individual cells.

 © The Author(s)

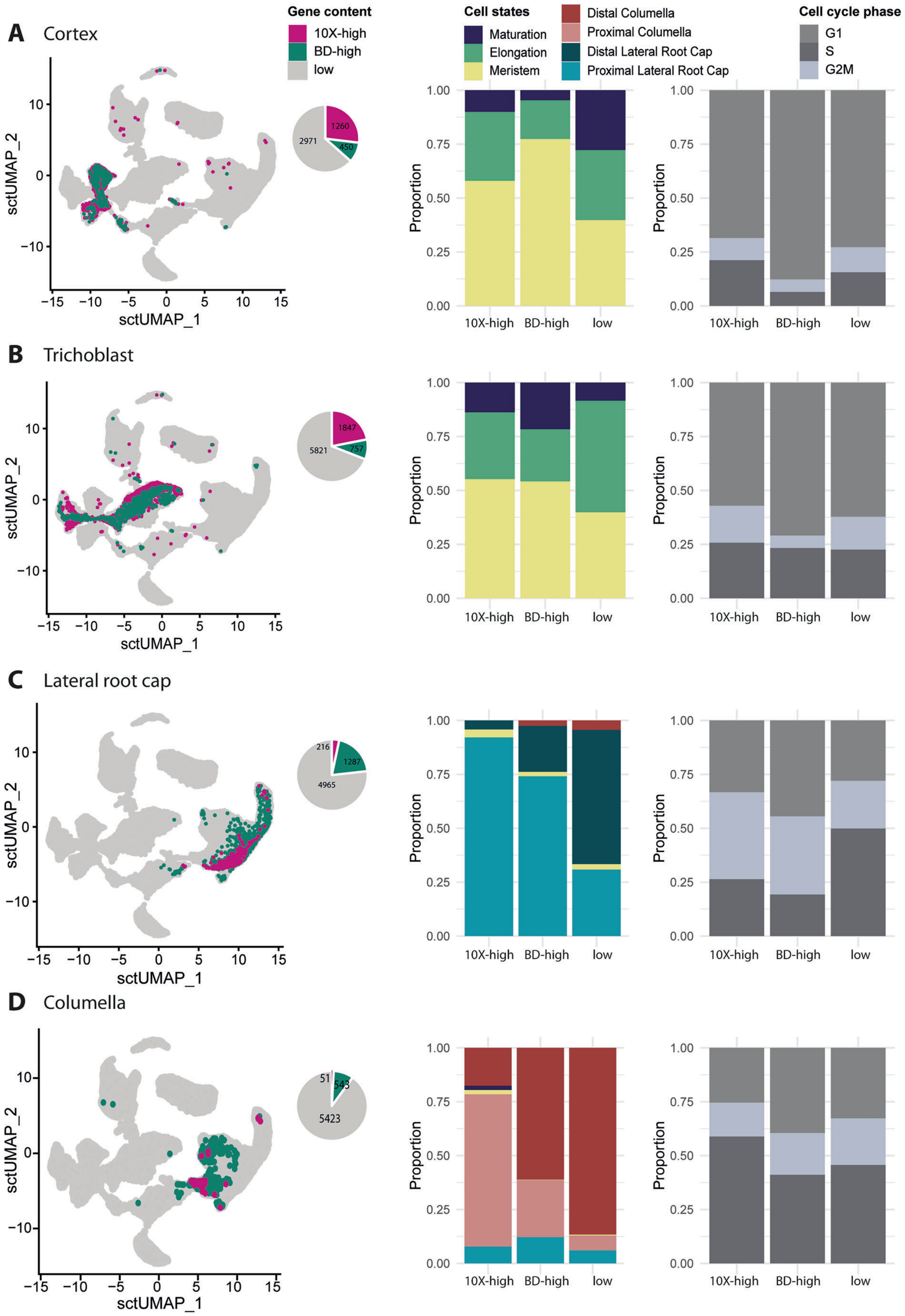
A Cortex
B Trichoblast
C Lateral root cap
D Columella
Gene content
10X-high
BD-high
low
Cell states
Maturation
Elongation
Meristem
Distal Columella
Proximal Columella
Distal Lateral Root Cap
Proximal Lateral Root Cap
Cell cycle phase
G1
S
G2M
sctUMAP_1
sctUMAP_2
Proportion
10X-high
BD-high
low
1260
450
2971
1847
757
5821
216
1287
4965
51
543
5423

Figure EV4. Platform-associated gene detection, cell state, and cell cycle phase distributions across major Arabidopsis root cell types.

(**A–D**) Analysis of four representative root cell types: (**A**) Cortex, (**B**) Trichoblast, (**C**) Lateral root cap, and (**D**) Columella. For each cell type UMAP projections of single-cell transcriptomes are shown (left panel), with cells colored by gene detection category: high-gene-content in 10X Chromium (magenta), high-gene-content in BD Rhapsody (green), and low-gene-content (gray). Pie charts indicate the number of cells in each category for each cell type. Bar plots (middle panel) represent the proportion of major cell states within each gene content category (10X-high, BD-high, low), as defined by established marker genes from Shahan et al, 2022. Distinct colors represent different cell states relevant to each cell type. Bar plots in the right panel show the distribution of cell cycle phases (G1, S, G2M) within each gene content category.

 © The Author(s)

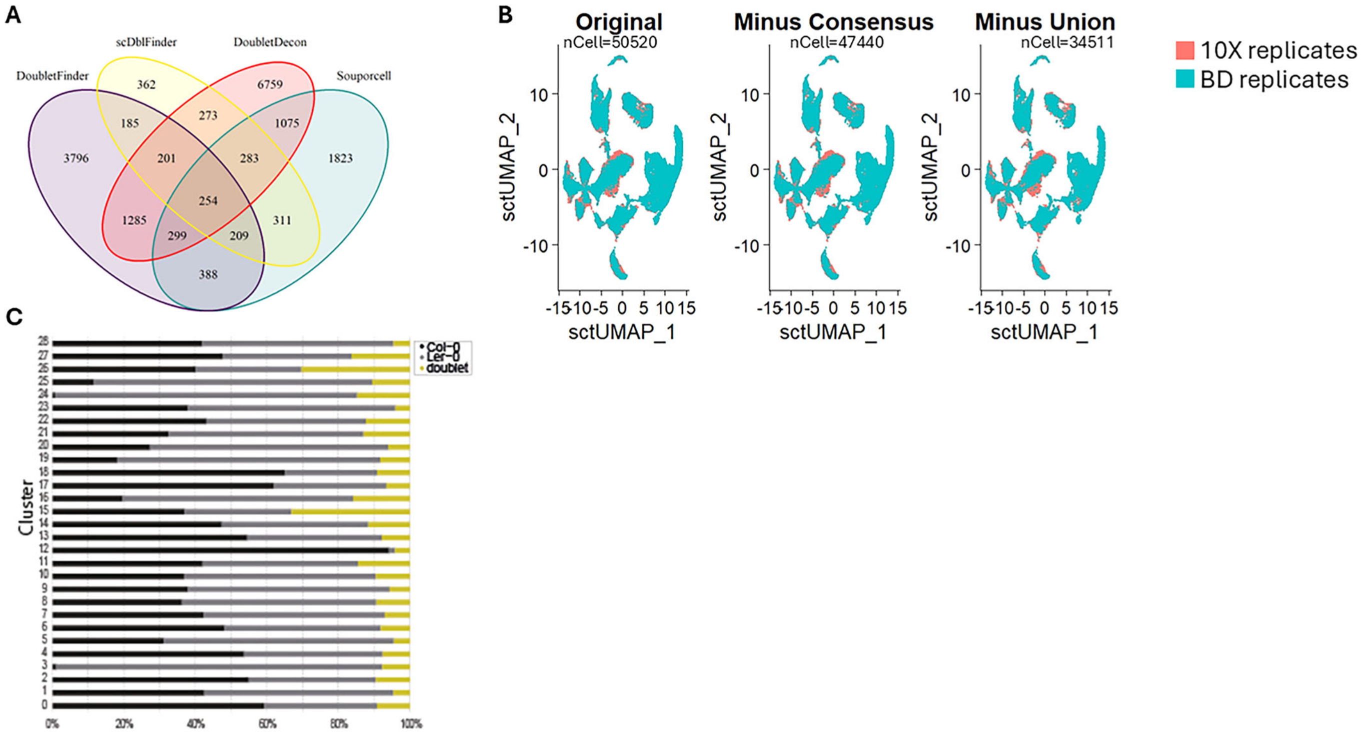

Figure EV5. Doublet detection across cell clusters.

(**A**) Venn diagram showing overlap and uniquely labeled doublets among *DoubletFinder, scDblFinder, DoubletDecon*, and *Souporcell*. (**B**) UMAP embeddings of the original dataset ($n = 50,520$ cells), after removal of "consensus" doublets (cells called by at least two tools; $n = 47,440$), and after removal of "union" doublets (cells called by at least two tools and confirmed as heterodimeric by SNPs; $n = 34,511$). Cells are colored by platform of origin (10X, orange; BD, blue). (**C**) Stacked bar plots showing, for each cluster (0-27), the proportions of Col-0 (black), Ler-0 (gray), and heterotypic doublets (yellow), with the *x* axis indicating the percentage of cells assigned to each category.
