# Supplementary material for: Benchmarking plant single cell RNA-sequencing sample processing strategies
Source: EMBO J. 2026 May 9;45(12):4337–59. doi: 10.1038/s44318-026-00800-5 (PMC13270049; doi:10.1038/s44318-026-00800-5)
Supplement: Supplementary file 2 — Peer Review File [file 44318_2026_800_MOESM2_ESM.pdf]

THE EMBO JOURNAL

Benchmarking plant single cell RNA-sequencing sample processing strategies

Thomas Eekhout, Lindsy Veirman, Jolien Block, Freya Persyn, Vera Goossens, Dominique Audenaert, Gert Isterdael, Bert Rybel, and Carolin Grones

Corresponding author(s): Carolin Grones (Carolin.Grones@wur.nl) , Bert Rybel (beryb@psb.vib-ugent.be)

Review Timeline:

Submission Date:	9th Jul 25
Editorial Decision:	8th Sep 25
Revision Received:	22nd Jan 26
Editorial Decision:	19th Mar 26
Revision Received:	20th Apr 26
Accepted:	22nd Apr 26

Editor: William Teale

Transaction Report:

(Note: With the exception of the correction of typographical or spelling errors that could be a source of ambiguity, letters and reports are not edited. Depending on transfer agreements, referee reports obtained elsewhere may or may not be included in this compilation. Referee reports are anonymous unless the Referee chooses to sign their reports.)

Dear Carolin,

Thank you again for the submission of your manuscript entitled "Performance evaluation of Arabidopsis scRNA-seq sample processing strategies" and for your patience during the review process. We have now received reports from three referees, which I copy below.

As you can see from their comments, all thought that your manuscript could represent a useful and timely resource for the community. That said, all of them point out some issues that will require your attention before your manuscript can be published in The EMBO Journal. I am most concerned about comments that refer to a lack of either statistical power, or validation of fractions containing larger cells after sorting.

Based on the overall interest expressed in the reports, however, I would like to invite you to address the comments of all referees in a revised version of the manuscript. I should add that it is The EMBO Journal policy to allow only a single major round of revision and that it is therefore important to resolve the main concerns at this stage. I believe the concerns of the referees are reasonable and addressable, but please contact me if you have any questions, need further input on the referee comments or if you anticipate any problems in addressing any of their points. Please, follow the instructions below when preparing your manuscript for resubmission.

I would also like to point out that as a matter of policy, competing manuscripts published during this period will not be taken into consideration in our assessment of the novelty presented by your study ("scooping" protection). We have extended this 'scooping protection policy' beyond the usual 3 month revision timeline to cover the period required for a full revision to address the essential experimental issues. Please contact me if you see a paper with related content published elsewhere to discuss the appropriate course of action.

When preparing your letter of response to the referees' comments, please bear in mind that this will form part of the Review Process File, and will therefore be available online to the community. For more details on our Transparent Editorial Process, please visit our website: https://www.embopress.org/page/journal/14602075/authorguide#transparentprocess

Again, please contact me at any time during revision if you need any help or have further questions.

Thank you very much again for the opportunity to consider your work for publication. I look forward to your revision.

Best regards,

William

William Teale, Ph.D.
Editor
The EMBO Journal

When submitting your revised manuscript, please carefully review the instructions below and include the following items:

1) a .docx formatted version of the manuscript text (including legends for main figures, EV figures and tables). Please make sure that the changes are highlighted to be clearly visible.

2) individual production quality figure files as .eps, .tif, .jpg (one file per figure).

3) a .docx formatted letter INCLUDING the reviewers' reports and your detailed point-by-point response to their comments. As part of the EMBO Press transparent editorial process, the point-by-point response is part of the Review Process File (RPF), which will be published alongside your paper.

4) a complete author checklist, which you can download from our author guidelines (https://wol-prod-cdn.literatumonline.com/pb-assets/embo-site/Author Checklist%20-%20EMBO%20J-1561436015657.xlsx). Please insert information in the checklist that is also reflected in the manuscript. The completed author checklist will also be part of the RPF.

5) Please note that all corresponding authors are required to supply an ORCID ID for their name upon submission of a revised manuscript.

6) We require a 'Data Availability' section after the Materials and Methods. Before submitting your revision, primary datasets produced in this study need to be deposited in an appropriate public database, and the accession numbers and database listed

under 'Data Availability'. Please remember to provide a reviewer password if the datasets are not yet public (see https://www.embopress.org/page/journal/14602075/authorguide#datadeposition). If no data deposition in external databases is needed for this paper, please then state in this section: This study includes no data deposited in external repositories. Note that the Data Availability Section is restricted to new primary data that are part of this study.

Note - All links should resolve to a page where the data can be accessed.

7) When assembling figures, please refer to our figure preparation guideline in order to ensure proper formatting and readability in print as well as on screen:
http://bit.ly/EMBOPressFigurePreparationGuideline

Please remember: Digital image enhancement is acceptable practice, as long as it accurately represents the original data and conforms to community standards. If a figure has been subjected to significant electronic manipulation, this must be noted in the figure legend or in the 'Materials and Methods' section. The editors reserve the right to request original versions of figures and the original images that were used to assemble the figure.

8) For data quantification: please specify the name of the statistical test used to generate error bars and P values, the number (n) of independent experiments (specify technical or biological replicates) underlying each data point and the test used to calculate p-values in each figure legend. The figure legends should contain a basic description of n, P and the test applied. Graphs must include a description of the bars and the error bars (s.d., s.e.m.).

9) We would also encourage you to include the source data for figure panels that show essential data. Numerical data can be provided as individual .xls or .csv files (including a tab describing the data). For 'blots' or microscopy, uncropped images should be submitted (using a zip archive or a single pdf per main figure if multiple images need to be supplied for one panel). Additional information on source data and instruction on how to label the files are available at .

10) We replaced Supplementary Information with Expanded View (EV) Figures and Tables that are collapsible/expandable online (see examples in https://www.embopress.org/doi/10.15252/embj.201695874). A maximum of 5 EV Figures can be typeset. EV Figures should be cited as 'Figure EV1, Figure EV2" etc. in the text and their respective legends should be included in the main text after the legends of regular figures.

- For the figures that you do NOT wish to display as Expanded View figures, they should be bundled together with their legends in a single PDF file called *Appendix*, which should start with a short Table of Content. Appendix figures should be referred to in the main text as: "Appendix Figure S1, Appendix Figure S2" etc. See detailed instructions regarding expanded view here: .

- Additional Tables/Datasets should be labelled and referred to as Table EV1, Dataset EV1, etc. Legends have to be provided in a separate tab in case of .xls files. Alternatively, the legend can be supplied as a separate text file (README) and zipped together with the Table/Dataset file.

11) At EMBO Press we ask authors to provide source data for the main manuscript figures. Our source data coordinator will contact you to discuss which figure panels we would need source data for and will also provide you with helpful tips on how to upload and organize the files.

12) Our journal encourages inclusion of *data citations in the reference list* to directly cite datasets that were re-used and obtained from public databases. Data citations in the article text are distinct from normal bibliographical citations and should directly link to the database records from which the data can be accessed. In the main text, data citations are formatted as follows: "Data ref: Smith et al, 2001" or "Data ref: NCBI Sequence Read Archive PRJNA342805, 2017". In the Reference list, data citations must be labeled with "[DATASET]". A data reference must provide the database name, accession number/identifiers and a resolvable link to the landing page from which the data can be accessed at the end of the reference. Further instructions are available at .

13) In order to increase the reproducibility and reach of your work, The EMBO Journal includes a table of reagents that were used in the study. Please provide this along with your revisions.

Further instructions for preparing your revised manuscript:

Please make sure you upload a letter of response to the referees' comments together with the revised manuscript.

Please also check that the title and abstract of the manuscript are brief, yet explicit, even to non-specialists.

When assembling figures, please refer to our figure preparation guideline in order to ensure proper formatting and readability in print as well as on screen:
https://bit.ly/EMBOPressFigurePreparationGuideline

See also guidelines for figure legends: https://www.embopress.org/page/journal/14602075/authorguide#figureformat

At EMBO Press we ask authors to provide source data for the main manuscript figures. You will receive a separate email with instructions for providing source data with your revised manuscript, including how to upload and organize the files.

IMPORTANT: When you send the revision we will require
- a point-by-point response to the referees' comments, with a detailed description of the changes made (as a word file).
- a word file of the manuscript text.
- individual production quality figure files (one file per figure)
- a complete author checklist, which you can download from our author guidelines (https://www.embopress.org/page/journal/14602075/authorguide).
- Expanded View files (replacing Supplementary Information)
Please see out instructions to authors
https://www.embopress.org/page/journal/14602075/authorguide#expandedview
- a Reagents and Tools Table as part of the Methods section, which can be downloaded from our author guidelines (https://www.embopress.org/page/journal/14602075/authorguide#structuredmethods)

Please remember: Digital image enhancement is acceptable practice, as long as it accurately represents the original data and conforms to community standards. If a figure has been subjected to significant electronic manipulation, this must be noted in the figure legend or in the 'Materials and Methods' section. The editors reserve the right to request original versions of figures and the original images that were used to assemble the figure.

Further information is available in our Guide For Authors: https://www.embopress.org/page/journal/14602075/authorguide

We realize that it is difficult to revise to a specific deadline. In the interest of protecting the conceptual advance provided by the work, we recommend a revision within 3 months (7th Dec 2025). Please discuss the revision progress ahead of this time with the editor if you require more time to complete the revisions. Use the link below to submit your revision:

https://emboj.msubmit.net/cgi-bin/main.plex

Referee #1:

This manuscript presents a comparative analysis of single-cell transcriptomic approaches and technologies in plants. The study is both novel and timely, addressing a critical gap in the benchmarking of single-cell -omics methods in plant biology. Unlike human and animal systems, plant biology presents unique technical and biological challenges. This includes spatially constrained tissue structures, broad cell size variability, genome ploidy due to endoreduplication, and the presence of rigid cell walls that complicate cell isolation.

The authors compare three protoplast isolation methods and two scRNAseq platforms, as well as evaluated the occurrence of doublets by computational algorithms using the Arabidopsis root system, the best studied system for scRNAseq. The approach is well structured and analyzed. However, there are aspects of the methodology, reasoning, and interpretation that require further clarification or development. In particular, some conclusions may be sensitive to methodological parameters, which are not fully considered. With these refinements, the manuscript would offer substantial value to researchers aiming to implement or benchmark single-cell transcriptomics in plants.

Major comments that must be addressed for the manuscript to become acceptable:
1. The observed differences in cell size for the various protoplast enrichment methods are very interesting. The manuscript suggests that large cells are underrepresented in flow cytometry samples due to its limitations, whereas magnetic levitation better preserves size heterogeneity (line 122). However, it is equally plausible that these larger cells are filtered out during flow cytometry because they are non-viable, clumped, or incompletely digested. We recommend that the authors investigate the size and morphology of viable versus non-viable cells in the magnetic levitation-enriched samples, ideally using their existing Revvity raw images. If cells that are not viable are consistently larger than non-viable ones, it would explain why these are not rescued during flow cytometry. If "larger cells" are also not round, they could also represent poorly digested cell clumps. Ideally, the comparison should be followed by identifying cell populations in scRNAseq that are missing from flow cytometry samples vs magnetic levitation. Additionally, these additional single cell datasets would allow the authors to truly show that cell viability as assessed by staining is directly correlated in the sequencing data. But we also understand that this latter demand might be beyond scope of the work.
2. In line 151 the authors state "BD Rhapsody exclusively detecting 791 genes that were not captured by 10X Chromium, while 10X Chromium uniquely detected 823 genes not found in BD Rhapsody samples [..] likely reflect fundamental technical

distinctions between BD Rhapsody's and 10X Chromium's approach and protocol". However, there appear to be some inconsistencies/errors in the numbers provided between the text and figures. In figure 2B, these numbers are different (845 and 98, respectively). In any case, the figure shows that there are as many genes uniquely detected in individual samples as between platforms. It is as likely that this is an artifact of random sampling as of a platform specific bias. Importantly, do these correspond primarily to lowly detected genes? The analysis would also benefit from an actual statistical analysis as well (some sort of Hypergeometric Test might be appropriate).
3. Correlation analyses are used throughout the manuscript, yet critical methodological details are missing:
a. Figure 2I: The manuscript states that Pearson correlation was used to assess variability in the top 3,000 variable genes, but it is unclear how variability was defined (across cells? across samples? between platforms?). Additionally, the correlation procedure, treatment of low-expression genes, and cell-level data handling are not described. This information should be included in the Methods section.
b. Figures 3D-E: The authors report lower correlation of marker gene expression between platforms than between replicates. However, this may reflect differences in cluster composition rather than true platform-specific bias. Many clusters appear dominated by one platform. Moreover, the analysis does not account for cluster size. We suggest performing the correlation analyses within individual clusters, particularly those evenly represented across platforms, to robustly assess "platform-specific biases on gene expression and biological interpretation". Also, Figure 3D lacks axis units and should be amended.
c. A final point that we feel that the authors should consider but for which we feel uneducated to give them valuable feedback is the validity of doing correlation analyses in batch-corrected samples. Batch correction algorithms, particularly those involving latent variable models or non-linear transformations, may distort the covariance structure of the data, potentially compromising the validity of downstream correlation metrics. As such, we caution that correlation-based interpretations derived from batch-corrected data may be confounded by artifacts introduced during the normalization and integration process, and recommend that the authors clarify how this issue was addressed or justified in their analysis.
4. Related to the above, the authors should test the robustness of their findings across different processing workflows. In particular, we encourage the authors to test and compare the outcome of utilizing different batch correction algorithms (e.g., Harmony vs Combat, Seurat's CCA, DESC, LIGER or MNN), and doublet finding algorithms (e.g. DoubletFinder vs scDblFinder, Solo, DoubletDecon or Scrublet).
5. In Figure 4A, cell types are defined as positions and labels. This does not correspond to the text in the legend. Please revise and amend.
6. The Data Analysis section lacks sufficient detail. Key steps such as normalization methods, gene filtering strategies, dimensionality reduction parameters, and statistical thresholds are under-described. For example, the use of FindMarkers (line 457), souporcell (line 470), and normalization (line 447) are not explained in sufficient detail to ensure reproducibility. Arbitrary cutoffs are presented without justification (e.g., lines 444-445, 479). The criteria for defining "low" and "high" confidence calls (e.g., line 490) are also unclear. We recommend expanding the Methods section or, ideally, making the analysis code available via a public repository (e.g., GitHub).

Minor comments that we encourage the authors to consider addressing:
1. The authors should make a clearer explanation on why they have focused specifically on those specific platforms and methods, while other methods are not considered (E.g., emulsion methods like PIP-Seq or combinatorial indexing like SPLiT-seq for scRNAseq; and density gradient for protoplast enrichment).
2. Figure 3C is presented but most elements in it are not explained.
3. A logarithmic scale for Figures 2E, F and G might be more informative to visualize actual differences.
4. Some gene names in figure 3E are truncated and need to me amended.
5. Because Seurat overlays dots, which makes it difficult to tell sample overlay at high densities. It would be great if either transparency or size of the dots were adjusted for the PCA (fig 2J) and UMAPs (3A&B, 4A, 5A), so all the dots are able to visualized when overlapping.
6. A UMAP of platform representation (like Figure 3A) on the batch corrected UMAP (figure 3B) would help visualize how well batch correction has worked on integrating cells across platforms.
7. Consider changing composition plots to bar plots (e.g., Figure 2A and C, Figure 5G). The current ones are not as helpful for visualization and comparison across different platforms/types or across different lines.
8. Lines 222-224: The method used for the analysis described in these lines is not specified in the Methods section. Please provide this information.
9. The discussion could be strengthened by exploring how these findings might inform meta-analyses of existing plant scRNA-seq datasets, particularly in relation to cross-study integration and benchmarking.

Referee #2:

In this paper, Eekhout et al., systematically compare protoplast enrichment methods (conventional and image-based flow cytometry, magnetic cell sorting) together with two scRNA-seq platforms (10x Genomics Chromium and BD Rhapsody) using Arabidopsis roots. They report that image-based flow cytometry enables precise cell selection, while magnetic sorting better preserves cell size heterogeneity. Both scRNA-seq platforms captured root cell diversity with reproducible gene expression, but platform-specific biases in cell type composition were observed. Furthermore, SNP analysis revealed that a substantial fraction

of doublets may be misclassified, providing important guidance for optimizing scRNA-seq workflows in plants. This manuscript is technically interesting and important for guiding plant single-cell research, and it has the potential to attract a broad readership. However, I have several concerns and comments listed below.

Major concerns and comments

One key limitation of the comparison between the 10x Genomics and BD Rhapsody platforms (Figure 2A-H) is the absence of statistical analysis across the biological replicates. Without such analysis, it is difficult to fully support the conclusions such as that BD achieves higher cell retention. Technical variability-for example, differences in protoplast handling during pipetting-can introduce substantial variation even within the same platform. This issue is evident in the authors' own data, where BD replicate-2 disproportionately contributes to cluster 24 in Figure 3C compared to BD replicate-1. To strengthen the conclusions, appropriate statistical tests should be performed on the biological replicates.
In addition, when comparing different cell cleaning methods, the authors should clarify how the "three technical replicates" described in the Methods (line 427) were defined.

Second, it would be helpful to check the GO enrichment or determine what kinds of genes are represented among the unique genes captured by either the BD or 10x platform. For example, do the 823 unique genes found by 10x Chromium belong to categories such as pseudogenes, ribosomal genes, or certain non-coding RNA genes that are excluded by BD when generating the reference genome?

Third, in Line 215-221, the authors propose that the BD Rhapsody system may preferentially capture cells in specific biological states-such as those exhibiting high translational activity or stress responses-thereby contributing to increased cellular heterogeneity within clusters. However, this interpretation is not supported by the design or functionality of the BD Rhapsody platform. During cell capture, the system does not assess or select cells based on transcriptional or translational activity; rather, it captures intact, viable cells without regard to their underlying molecular profiles. Therefore, the suggestion that BD Rhapsody selectively enriches for certain biological states is mechanistically implausible. This portion of the discussion should be revised to avoid overstating the implications of the observed gene set enrichment.

Fourth, the observation that BD Rhapsody yielded a higher proportion of epidermis, initials, and lateral root cap cells, while 10x Chromium enriched for cortex and endodermis cells, is very interesting and suggests a platform-dependent bias in cell type recovery. One possible explanation is a difference in cell size that affects capture efficiency between the microwell-based BD Rhapsody system and the droplet-based 10x Chromium platform. To test this hypothesis, the authors could use cell type-specific promoter reporter lines to visualize and quantify cell sizes across these populations. This would provide direct evidence as to whether size differences contribute to the observed capture bias and help clarify whether physical properties of cells influence platform-specific representation.

Minor comments:
Line 319. The authors state that flow cytometry substantially enhanced cell viability. I disagree with this interpretation, as the main function of flow cytometry is to remove debris and dead cells rather than directly improving viability. Please rephrase this statement accordingly.

In Figure 3B, it would be helpful to include a UMAP of cells grouped by different replicates of 10x and BD after batch correction to demonstrate that the Harmony algorithm effectively removed the batch effect.

An estimated 20,000 cells from the same protoplast sample were simultaneously loaded as input on both the 10x Chromium (10x) and BD Rhapsody (BD) platforms. However, this does not account for potential differences in recovery rate between the two platforms. If the recovery rates were not the same, then sequencing each library to a similar depth of 150 million reads could result in variation in sequencing depth per cell.

How do the two platforms compare in their ability to capture quiescent center cells? Given their low abundance, even small differences in recovery efficiency between 10x Chromium and BD Rhapsody could lead to significant discrepancies in representation. It would be helpful if the authors could comment on whether such rare populations were differentially captured or detected across the platforms.

An estimated 20,000 cells... → typo: should be 20,000 cells

Referee #4:

Review: Performance evaluation of Arabidopsis scRNA-seq sample processing Strategies
The authors work to optimise the workflow of single cell sequencing methodologies, from the method of cell isolation through to the integration and analyses of the cell populations at the bioinformatic level. This paper is of interest to people working with this single cell transcriptomics in plant materials - a relatively small but growing community. I think the paper offers some valuable

insights that can help guide people through this challenging and expensive experimental approach. I think that the authors set the scene with an appropriate level of background information and clearly explain the reasoning for putting together this body of work. I do however think that this manuscript lacks robust conclusions and contains a high level of speculative and unclear interpretations. I am also concerned by the lack of statistical analyses reported throughout. While I make comments relating to specific parts of the manuscript below, these can be read as general comments as I observed the same issues throughout.
1) I think it was valuable to assess the degree of cell size heterogeneity maintained across cell capture methods compared to unsorted samples. It seems clear that Levitation is better for capturing the heterogeneity that exists in the root tissue. It would be good to see replication here, however.
2) The authors state the high importance of cell viability and then opt to use a method with a 20% lower cell viability count. I can see arguments for prioritizing cell size heterogeneity over viability, but the authors should explain their rationale here. For me viability seems like the lesser evil with the possibility to remove low quality cells at the QC level bioinformatically.
3) I find Figure 1 panel B confusing. I made sense of it, but the keys used are unnecessary. Maybe consider a proportion/stacked bar chart per technology for example.
4) I find the comparisons made useful in many cases but many of the conclusions are too vague or speculative and offer no guidance for future researchers.
5) The venn diagram in Figure 2 shows the overlap of genes identified in each platform and replicate. The authors state that " BD Rhapsody exclusively detecting 791 genes that were not captured by 10X Chromium, while 10X Chromium uniquely detected 823 genes not found in BD Rhapsody samples." The "791" however is just for one replicate of BD and I cannot see where the"823" comes from for 10x. I can only see 823 for the second BD replicate.

Overall, while I think this study could in the future be of help to people planning these difficult experiments, the power of this study has not yet been realised.

Reply to Reviewer Comments: Reviewer #1

This manuscript presents a comparative analysis of single-cell transcriptomic approaches and technologies in plants. The study is both novel and timely, addressing a critical gap in the benchmarking of single-cell -omics methods in plant biology. Unlike human and animal systems, plant biology presents unique technical and biological challenges. This includes spatially constrained tissue structures, broad cell size variability, genome ploidy due to endoreduplication, and the presence of rigid cell walls that complicate cell isolation. The authors compare three protoplast isolation methods and two scRNAseq platforms, as well as evaluated the occurrence of doublets by computational algorithms using the Arabidopsis root system, the best studied system for scRNAseq. The approach is well structured and analyzed. However, there are aspects of the methodology, reasoning, and interpretation that require further clarification or development. In particular, some conclusions may be sensitive to methodological parameters, which are not fully considered. With these refinements, the manuscript would offer substantial value to researchers aiming to implement or benchmark single-cell transcriptomics in plants.

=> We thank the reviewer for highlighting the potential and timeliness of our manuscript and appreciate the suggestions to further improve our work as elaborated on below.

Major remarks:

1. The observed differences in cell size for the various protoplast enrichment methods are very interesting. The manuscript suggests that large cells are underrepresented in flow cytometry samples due to its limitations, whereas magnetic levitation better preserves size heterogeneity (line 122). However, it is equally plausible that these larger cells are filtered out during flow cytometry because they are non-viable, clumped, or incompletely digested. We recommend that the authors investigate the size and morphology of viable versus non-viable cells in the magnetic levitation-enriched samples, ideally using their existing Revvity raw images. If cells that are not viable are consistently larger than non-viable ones, it would explain why these are not rescued during flow cytometry. If "larger cells" are also not round, they could also represent poorly digested cell clumps. Ideally, the comparison should be followed by identifying cell populations in scRNAseq that are missing from flow cytometry samples vs magnetic levitation. Additionally, these additional single cell datasets would allow the authors to truly show that cell viability as assessed by staining is directly correlated in the sequencing data. But we also understand that this latter demand might be beyond scope of the work.

=> We thank the referee for this thoughtful and constructive comment. We agree that analyzing the relationship between cell size and viability provides important context for interpreting the differences observed between the enrichment methods. To address this, we repeated the experiments and extended our morphological comparison to all experimental conditions (no purification, magnetic levitation, flow cytometry and image-based flow cytometry) to evaluate whether viable and non-viable cells differ in cell diameter or shape (included as **Fig EV 1**). Viable (Calcein-positive) and non-viable (PI-positive) cells showed similar cell diameter (**Fig EV1A-B**) and roundness (**Fig EV1C**) in the unsorted samples and after magnetic levitation, arguing against a systematic enrichment of large, irregular aggregates among non-viable cells. After image-based flow cytometry sorting, alive cells exhibited a lower cell diameter than dead cells, while dead cells displayed reduced roundness compared to alive cells. Roundness did not differ significantly between alive and dead cells in

the unsorted samples, indicating that these irregular shapes and the slight increase in cell diameter in non-viable cells are likely induced by the sorting procedure rather than reflecting pre-existing clumps. No correlation was detected between cell diameter and roundness in alive or dead cells in any condition (**Fig EV1D**), further arguing against large, irregular aggregates prior to flow cytometry as the main cause of reduced recovery.

Together, these analyses indicate that magnetic levitation preserves cell morphology and that larger cells are not consistently enriched among non-viable populations. The under-representation of large cells in flow-cytometry samples therefore appears unlikely to be explained solely by differences in viability and is more plausibly related to technical constraints of the sorting systems in efficiently capturing larger cells. We have adapted the details and conclusions from this analysis in the revised manuscript (line 109-128): "*After cell sorting viability increased to 94% and 87% for image-based and regular flow cytometry, respectively, compared to 51% viability in the non-purified sample (**Fig 1D**). Viability after magnetic levitation reached 72%. To estimate the effect of flow cytometry versus magnetic levitation-based cell separation on preserving the original cell size heterogeneity among root cells, we quantified protoplast size (defined as cell diameter) distributions after applying different enrichment strategies (**Fig 1E**). Magnetic levitation outperformed both flow cytometers in maintaining the original size heterogeneity of viable protoplasts (**Table EV2**): viable Arabidopsis root cells purified by magnetic separation displayed a broad and representative range of cell sizes (mean cell diameter=12.6 µm; cells larger than 20 µm accounted for 7% of all analyzed cells), comparable to the unsorted sample (mean cell diameter=14 µm; cells larger than 20 µm accounted for 12% of all analyzed cells), whereas both flow cytometers yielded a narrower size range biased toward smaller cells (mean cell diameter= 10.3/11.6; cells larger than 20 µm accounted for only 2% of the population). In unsorted samples and after magnetic levitation, viable and dead cells showed similar cell diameter distribution and roundness (**Fig EV1A–C**), with no correlation between these parameters (**Fig EV1D**). After image-based flow cytometry sorting, alive cells were smaller than dead cells, and dead cells showed reduced roundness (**Fig EV1A–C**). Because no differences were observed in the unpurified samples, and diameter and roundness were uncorrelated in all conditions (**Fig EV1D**), these changes likely result from shearing forces during sorting rather than pre-existing large or irregular aggregates.*"

We also agree with the referee that transcriptomic profiling of cells across size ranges would represent the ultimate validation of whether large cells are compatible with the single cell platforms used in this study. However, both the 10X Chromium and BD Rhapsody systems recommend a maximum input cell size of ~40 µm, and in our enriched samples, larger cells often exceed this threshold. This introduces a significant risk of chip clogging, particularly for the 10X Chromium platform, making such experiments technically challenging and unreliable in our setup. To circumvent these limitations, alternative technologies such as the iCell8 or Shasta (Takara) platforms—which allow user-guided cell selection and thus provide a direct link between cellular phenotype and transcriptome—would be better suited to address this specific question. Since these platforms were not employed in the present study, and in line with the referee's own remark, we consider such experiments to be beyond the current scope.

2. In line 151 the authors state "BD Rhapsody exclusively detecting 791 genes that were not captured by 10X Chromium, while 10X Chromium uniquely detected 823 genes not found in BD Rhapsody samples [..] likely reflect fundamental technical distinctions between BD Rhapsody's and 10X Chromium's approach and protocol". However, there appear to be some inconsistencies/errors in the numbers provided between the text and figures. In figure 2B,

these numbers are different (845 and 98, respectively). In any case, the figure shows that there are as many genes uniquely detected in individual samples as between platforms. It is as likely that this is an artifact of random sampling as of a platform specific bias. Importantly, do these correspond primarily to lowly detected genes + GO terms? The analysis would also benefit from an actual statistical analysis as well (some sort of Hypergeometric Test might be appropriate).

=> We thank the referee for carefully noting the inconsistency between the numbers reported in the text and **Fig 2B**. We repeated the analysis and identified an error in the previously reported numbers. As suggested, we further characterized the genes uniquely detected by each platform (now provided in **Table EV4**). However, "lowly expressed" can be defined either in terms of absolute counts or fraction of cells expressing a gene, and these definitions are not standardized in single-cell RNA-seq. We therefore calculated the proportion of cells in which the technology-specific gene was detected (**Table EV4**). The majority of technology-specific genes were found in less than 0.05% of all cells (~25 cells; see figure below).

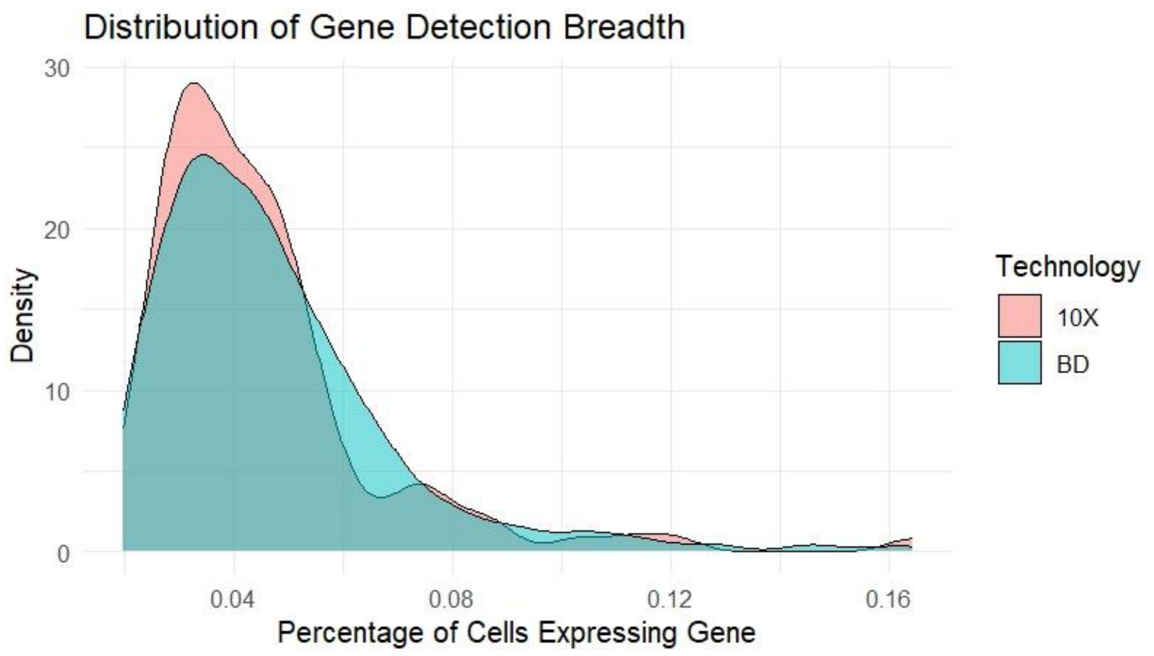

We found that 30% of the 10X-specific genes and 37% of the BD-specific genes were expressed in more than 25 cells, indicating that a substantial subset of platform-specific genes exhibits relatively broad expression rather than being confined to a few rare cells. To investigate the biological nature of these genes, we performed GO term enrichment analysis separately for the 10X-specific and BD-specific gene sets. For 10X-specific genes, no GO category reached statistical significance after multiple testing correction. BD-specific genes showed significant enrichment for "RNA modification" ($p = 0.03$), including several Pentatricopeptide repeat (PPR) proteins, and for "protein binding" ($p = 1\times10^{-4}$), encompassing, for example, *SCARECROW-LIKE 28*, *MYB101*, and *WUSCHEL*.

We also followed the referee's suggestion to formally assess whether the number of platform-specific genes exceeds what would be expected by random sampling. Because a classical hypergeometric test evaluates the significance of the overlap between sets rather than the size of non-overlapping fractions, we instead used a permutation-based strategy. We randomly assigned two samples as 10X and two samples as BD and, for each permutation, calculated how many genes would appear "platform-specific" under this random grouping. The observed numbers of 10X-specific and BD-specific genes did not exceed this null

distribution (all empirical p-values > 0.05), indicating that the extent of apparent platform-specific detection is not greater than expected by chance. These results are now included in the revised manuscript (line 159-176): "*For 10X Chromium, the two replicates shared 105 uniquely detected genes, with an additional 174 and 123 uniquely detected genes in replicates 1 and 2, respectively (**Fig 2B**; **Table EV4**). For BD Rhapsody, the replicates shared 671 uniquely detected genes, with an additional 284 and 495 genes uniquely detected in replicates 1 and 2, respectively. 30% of 10X-specific and 37% of BD-specific genes were expressed in more than 25 cells, indicating that many platform-specific genes are not restricted to a few rare cells but show relatively broad expression. No GO category reached statistical significance for the 10X-specific genes, whereas BD-specific genes showed significant enrichment for 'RNA modification' (p = 0.03), including several Pentatricopeptide repeat (PPR) proteins, and for 'protein binding' ($p = 1\times10^{-4}$), including, for example, SCARECROW-LIKE 28, MYB101, and WUSCHEL. The overall number of platform-specific genes was, however, compatible with random sampling variation rather than a strong, systematic platform bias in gene detection. These platform-specific gene sets are therefore best interpreted as reflecting subtle technical distinctions between BD Rhapsody and 10X Chromium (e.g. differences in cell/RNA capture, library preparation, and sensitivity to low-expression genes), as well as inherent variability in transcript recovery across cell types. Given the limited number of biological replicates (two per platform), we did not attempt formal between-replicate statistical testing.*"

3. Correlation analyses are used throughout the manuscript, yet critical methodological details are missing:

a. Figure 2I: The manuscript states that Pearson correlation was used to assess variability in the top 3,000 variable genes, but it is unclear how variability was defined (across cells? across samples? between platforms?). Additionally, the correlation procedure, treatment of low-expression genes, and cell-level data handling are not described. This information should be included in the Methods section.

=> We thank the referee for pointing out the need for additional methodological detail regarding our correlation analysis. In the revised manuscript, we have expanded the description of the computational workflow both in the Figure legend and the Methods sections. Specifically, we now clarify in the legend of **Fig 2** that "*The top 3,000 variable genes were defined based on their expression variance across all cells from the four samples.*" Furthermore, we specify (line 560-563) that "*Principal component analysis (PCA) was performed using the top 3000 highly variable genes. After processing each sample separately, the four objects were merged and SCTransform was run again on the merged object, leading to a list of 3000 HVGs across all cells from the four samples.*" This additional detail ensures transparency and reproducibility of the correlation analyses presented in **Fig 2I**.

b. Figures 3D-E: The authors report lower correlation of marker gene expression between platforms than between replicates. However, this may reflect differences in cluster composition rather than true platform-specific bias. Many clusters appear dominated by one platform. Moreover, the analysis does not account for cluster size. We suggest performing the correlation analyses within individual clusters, particularly those evenly represented across platforms, to robustly assess "platform-specific biases on gene expression and biological interpretation". Also, Figure 3D lacks axis units and should be amended.

=> In the revised version of the manuscript, we have modified the representation of the analysis originally shown in **Fig 3D** to make its interpretation clearer. In the initial submission, **Fig 3D** summarized correlations of average expression values of the top 5 marker genes per cluster across platforms and replicates, but this was not fully evident from the previous visualization. To directly address the concern that differences in correlation might reflect cluster composition or cluster size rather than true platform-specific biases, we now perform the correlation analyses within individual clusters using pseudo-bulk expression profiles of the top 5 cluster-specific marker genes. Specifically, for each cluster we aggregate counts across cells to obtain a pseudo-bulk profile per sample and then correlate these profiles across replicates and platforms. This approach controls for unequal cell numbers and avoids distortion from cluster size differences, because correlations are computed on averaged expression values rather than individual cells. This analysis shows that correlations between biological replicates within the same platform are high and consistent for almost all clusters, with the exception of clusters 0, 21 (BD replicates only), and 27. In contrast, differences between platforms are more pronounced and are particularly evident for clusters 2, 8, 19, and 26. Importantly, none of these four clusters displays a strong platform-biased cell composition, indicating that the reduced correlation between platforms cannot be explained solely by differences in cluster composition. These findings are now described in the revised Results section (lines 236–247): "*To further investigate the impact of platform-specific biases on gene expression and biological interpretation, we correlated gene expression patterns of the top 5 cluster-specific marker genes across replicates and platforms (**Fig 3D, Table EV5**). Gene expression of top marker genes remained highly correlated within replicates of both 10X Chromium and BD Rhapsody (with the exception of clusters 0, 21 for BD, and 27), whereas substantially lower correlations were observed when comparing the two platforms, particularly for clusters 2, 8, 19, and 26. As none of these clusters displayed a pronounced platform-biased cell composition, these platform-specific differences at the gene expression level cannot be attributed solely to differences in cell number or platform-biased composition and likely reflect residual platform-dependent technical effects in cell and RNA capture. In line with this, we identified clusters in which the percentage of cells expressing the top five marker genes differed substantially between platforms (**Fig 3E**) [..]*"

Finally, we have updated **Fig 3D** in the revised manuscript to include explicit axis labels and units (Pearson correlation coefficient) and simplified the layout to more clearly convey the comparison between within-platform replicates and between-platform pairs.

c. A final point that we feel that the authors should consider but for which we feel uneducated to give them valuable feedback is the validity of doing correlation analyses in batch-corrected samples. Batch correction algorithms, particularly those involving latent variable models or non-linear transformations, may distort the covariance structure of the data, potentially compromising the validity of downstream correlation metrics. As such, we caution that correlation-based interpretations derived from batch-corrected data may be confounded by artifacts introduced during the normalization and integration process, and recommend that the authors clarify how this issue was addressed or justified in their analysis.

=> We thank the referee for raising this important point regarding the validity of correlation analyses on batch-corrected data. We fully agree that certain batch correction approaches—particularly those relying on latent variable models or strong non-linear transformations—can alter the covariance structure of the data and may therefore confound correlation-based analyses. To address this, we have clarified in the legend for **Fig 3D** that "*The normalized but non-batch-corrected count matrix was used to aggregate counts across cells to obtain a pseudo-bulk profile per sample, which was then correlated across replicates and platforms.*".

Using the normalized but non-batch-corrected counts ensures that the covariance structure was preserved. Batch correction was applied only for visualization and clustering purposes (e.g., UMAP representations). This strategy is consistent with common recommendations to reserve batch correction for integration/visualization, while performing correlation and co-expression analyses on normalized but otherwise uncorrected expression values (Micheletti et al., 2024, *Bioinformatics*; doi: 10.1093/bioinformatics/btae531)).

4. Related to the above, the authors should test the robustness of their findings across different processing workflows. In particular, we encourage the authors to test and compare the outcome of utilizing different batch correction algorithms (e.g., Harmony vs Combat, Seurat's CCA, DESC, LIGER or MNN), and doublet finding algorithms (e.g. DoubletFinder vs scDblFinder, Solo, DoubletDecon or Scrublet).

=> We thank the referee for this suggestion. We performed additional analyses to test the robustness of our findings across different processing workflows. Specifically, we compared several batch correction strategies -no batch correction, Harmony, Seurat's CCA, and MNN (**Fig EV2, Appendix Figure S3**)- and multiple doublet detection methods -scDblFinder, DoubletDecon and DoubletFinder (**Fig EV5**). For batch correction, we examined cluster structure and sample/technology composition under each method (**Fig EV2, Appendix Figure S3**). Harmony produced the mildest integration, preserving some technology-specific clusters, whereas CCA and MNN yielded stronger mixing and near-complete overlap of clusters across technologies (**Fig EV2**), consistent with prior benchmarks of integration strength. Despite these differences in integration strength, the major cell populations as well as the platform-specific gene expression biases described in the main text (**Fig 3E–F**) remained stable across methods (**Fig EV2, Appendix Figure S3**). These results are shown in **Fig EV2** and **Appendix Figure S3**, and we now state in the revised manuscript (line 250-253): "*These observations were robust to the choice of batch correction (no batch correction, Harmony, CCA, MNN;* ***Fig EV2****), which yielded similar cluster structures and preserved the observed platform-specific differences in cluster-specific marker genes (****Appendix Figure S3****).*"

For doublet detection, we now applied four computational tools with distinct underlying strategies: DoubletFinder, scDblFinder, DoubletDecon, and Souporcell, reflecting current best-practice recommendations to compare multiple algorithms. Across these methods, 254 droplets were consistently flagged as doublets, while each tool also produced method-specific calls (**Fig EV5A**). DoubletFinder, which is used in the main analysis, uniquely labeled 3,796 doublets, scDblFinder 362, DoubletDecon 6,759, and Souporcell 1,823. This degree of discordance is in line with previous benchmarks reporting substantial variability in doublet calls across tools. To quantify this variability, we benchmarked the doublet-detection tools using our genotype annotation as an internal reference (4,596 genotype-confirmed true doublets). DoubletFinder correctly identified 1,368 doublets but missed 3,228 heterodimeric doublets and flagged 6,201 genotype-negative cells as doublets (precision 0.18, recall 0.30, F1 0.22), meaning that it recovers about 30% of true doublets but at the cost of incorrectly labeling roughly 4–5 singlets for every true doublet. DoubletDecon correctly identified 1,893 doublets, missed 2,703 heterodimeric doublets, and labeled 8,518 genotype-negative cells as doublets (precision 0.18, recall 0.41, F1 0.25), indicating higher sensitivity but a similarly high false-positive rate. In contrast, scDblFinder correctly identified 750 doublets, missed 3,846 heterodimeric doublets, and called 765 genotype-negative cells as doublets (precision 0.50, recall 0.16, F1 0.25). Thus, when scDblFinder predicts a doublet, there is about a 50% chance that it is a true doublet, but its low recall means it fails to detect more than 80% of the genotype-confirmed doublets.

We further assessed the impact of doublet removal on downstream analyses. We removed either (i) only "consensus" doublets (cells called as doublets by at least two methods) or (ii) the union of predicted doublets across all tools plus all genotype-confirmed doublets. The consensus strategy removed 3,080 cells, whereas the union strategy removed 16,009 cells (32% of all cells; **Fig EV5B**). Importantly, neither strategy substantially altered the overall cluster structure or the platform-specific cell cluster representation (**Table EV7**). For instance, after union doublet removal, platform contributions to most clusters remained within ±10% of the original proportions; only clusters 4 and 24 showed an increase in the overall contribution of 10x-derived cells to the cluster (by 13% and 33%, respectively), and clusters 17 and 26 showed an increase in the overall contribution of BD-derived cells to the cluster (by 14% and 10%). Union doublet removal eliminated 55% of the cells in cluster 15 (previously identified as doublet-enriched based on our genotype-based doublet identification), whereas consensus-based removal eliminated only 2.5% of cells in this cluster, underscoring that individual prediction tools alone are not very accurate at identifying plant doublets.

Together, these analyses show that different doublet-detection algorithms make markedly different trade-offs between sensitivity (recall) and specificity (precision) and even aggressive doublet removal (union strategy) leaves the key platform-specific patterns in cluster composition and marker gene expression largely unchanged, and that our main conclusions are robust across doublet identifiers. We have included these results in the revised manuscript (line 334-350): "*To put these results in context, we also applied three additional doublet-identification tools with distinct underlying strategies: scDblFinder (Germain et al, 2022), DoubletDecon (DePasquale et al, 2019), and Souporcell (Heaton et al., 2020). Across all four methods, 254 droplets were consistently flagged as doublets, while each tool produced substantial numbers of method-specific calls (**Fig EV5, Table EV7**). When benchmarked against the same SNP-based ground truth, scDblFinder and DoubletDecon achieved F1 scores of 0.25, reflecting different trade-offs between precision and recall but similarly modest overall accuracy. We then assessed the impact of doublet removal on downstream analyses by removing either (i) only 'consensus' doublets (cells flagged by at least two methods) or (ii) the union of predicted doublets from all tools together with all genotype-confirmed doublets. The consensus strategy removed 3,080 cells, whereas the union strategy removed 16,009 cells (32% of all cells; **Fig EV5A-B**), yet neither approach substantially altered the overall cluster structure or the platform-specific cluster representation (**Table EV7**). Given the uniformly low F1 scores across all methods and the limited impact of even aggressive doublet removal on downstream results, these analyses indicate that computational doublet identification for plant scRNA-seq data lacks precision and remains challenging.*"

5. In Figure 4A, cell types are defined as positions and labels. This does not correspond to the text in the legend. Please revise and amend.

=> We have provided a more precise Figure Text for **Fig 4A** in the resubmitted manuscript: "*UMAP showing different cell identities. Cell types demonstrating a notable bias in representation between the 10X Chromium and BD Rhapsody platforms are highlighted: cortex cells (magenta) are over-represented in 10X Chromium, whereas Epidermis and Lateral Root Cap cells (dark green) and cells classified as 'Initials' (light green) are over-represented in BD Rhapsody replicates [...]*".

6. The Data Analysis section lacks sufficient detail. Key steps such as normalization methods, gene filtering strategies, dimensionality reduction parameters, and statistical thresholds are under-described. For example, the use of FindMarkers (line 457), souporcell (line 470), and normalization (line 447) are not explained in sufficient detail to ensure reproducibility. Arbitrary cutoffs are presented without justification (e.g., lines 444-445, 479). The criteria for defining "low" and "high" confidence calls (e.g., line 490) are also unclear. We recommend expanding the Methods section or, ideally, making the analysis code available via a public repository (e.g., GitHub).

=> We thank the referee for highlighting the need for greater detail in the Method section. We agree that transparent reporting of computational steps is essential for reproducibility. In the revised manuscript, we have substantially expanded the description of our computational pipeline. Specifically, we now provide detailed information on sequencing-depth subsampling, how platform-specific genes were determined, how data integration was performed, how the gene content was classified as high or low and what the high and low confidence calls for DoubletFinder entail.

Minor comments that we encourage the authors to consider addressing:

=> All suggested minor comments regarding figure details and formatting have been addressed in the resubmitted manuscript.

1. The authors should make a clearer explanation on why they have focused specifically on those specific platforms and methods, while other methods are not considered (E.g., emulsion methods like PIP-Seq or combinatorial indexing like SPLiT-seq for scRNAseq; and density gradient for protoplast enrichment).

=> We clarified our reasoning for focusing this study on only two single-cell platforms in the Discussion section (see line 362-368: "*We limited the technology benchmark to two cell sorters, a magnetic levitation assay, and two scRNA-seq platforms: droplet-based (10X Chromium) and microwell-based (BD Rhapsody). While other methods, such as emulsion-based approaches (e.g., PIP-Seq), combinatorial indexing (e.g., SPLiT-seq), or alternative protoplast enrichment strategies (e.g., density gradients; Jo et al., 2025) exist, our goal was to systematically compare platforms that are most commonly employed in plant single cell studies under standard processing conditions.*" We hope this addresses the reviewer's remark sufficiently.

2. Figure 3C is presented but most elements in it are not explained.

=> We added more explanation to the Result section (see line 222-226): "*Clusters with roughly equal contributions from both platforms (40–50% per platform; e.g., clusters 1, 2, 4, 7, 16, 20, and 23) were considered unbiased (**Fig 3C**). Most clusters showed a slight imbalance, with one platform contributing only 25–40% of the cells. By contrast, clusters 0, 15, 24, and 27 exhibited a marked bias, with one platform contributing less than 25% of the cells.*" and included a more detailed explanation in the text of **Fig 3C**: "*Clusters with a technology bias were defined as those where one technology contributes less than 25% of cells. Clusters with roughly equal contributions from both technologies (40–50% per technology; e.g., Clusters 1, 2, 4, 7, 16, 20, 23) were considered unbiased.*"

3. A logarithmic scale for Figures 2E, F and G might be more informative to visualize actual differences.

=> We agree with the suggestion and changed the Figure panels accordingly.

4. Some gene names in figure 3E are truncated and need to me amended.

=> We have aligned the sizes of the gene names in the resubmitted manuscript.

5. Because Seurat overlays dots, which makes it difficult to tell sample overlay at high densities. It would be great if either transparency or size of the dots were adjusted for the PCA (fig 2J) and UMAPs (3A&B, 4A, 5A), so all the dots are able to visualized when overlapping.

=> We agree that overplotting in Seurat visualizations can make it difficult to assess sample overlap at high cell densities. In response, we have reduced the dot size in the PCA (**Fig 2J**) and UMAPs (**Fig 3A-B**, **Fig 4A**, and **Fig 5A**) to improve visualization of overlapping cells. We also explored other options, such as adjusting color schemes and transparency; however, these approaches did not sufficiently resolve the issue and could potentially impair point recognition in printed (non-digital) versions of the figures. We hope the revised plots now provide a clearer representation of cell distributions.

6. A UMAP of platform representation (like Figure 3A) on the batch corrected UMAP (figure 3B) would help visualize how well batch correction has worked on integrating cells across platforms.

=> We have added this UMAP to **Fig 3A**.

7. Consider changing composition plots to bar plots (e.g., Figure 2A and C, Figure 5G). The current ones are not as helpful for visualization and comparison across different platforms/types or across different s.

=> We have modified the figure panels mentioned (**Fig 2A, Fig 2 C, Fig 5G**) accordingly. However, for other composition plots we chose to retain the original format due to the scale and complexity of the data presented. Converting these into bar plots would have substantially reduced clarity and made comparisons more difficult to interpret. We believe the current representation provides a more comprehensive and interpretable visualization of the data.

8. Lines 222-224: The method used for the analysis described in these lines is not specified in the Methods section. Please provide this information.

=> We apologize for not including the information on the GO term analysis. We have corrected our mistake and included it in the Methods section of the resubmitted manuscript.

9. The discussion could be strengthened by exploring how these findings might inform meta-analyses of existing plant scRNA-seq datasets, particularly in relation to cross-study integration and benchmarking.

=> We want to thank the referee for this suggestion and expanded the Discussion to include considerations on drawing conclusions from cross-study integration and benchmarking (see line 449-460): "*Together, these results provide critical insights for cross-study integration of plant single-cell datasets. While integration methods aim to harmonize data from diverse sources for comparative analysis (Luecken et al., 2021; Zhong et al., 2025), our results highlight that challenges extend beyond bioinformatic correction (He et al., 2024) and originate already at the level of plant-specific sample generation and processing. First, precise documentation of cell isolation and enrichment protocols (e.g., Grones et al., 2024) is essential to evaluate the impact of cell size heterogeneity and cell type capture efficiency*

*across studies. Second, platform-specific biases and technical variation persist even after batch correction, affecting both cell type representation and doublet composition. These biases must be explicitly considered when comparing cell type proportions, biological pathways, or stress-related signatures across studies. To support future efforts, we summarize key considerations for protocol design, data interpretation, and large-scale integration projects in plant single-cell biology in **Table 1**."*

Reply to Reviewer Comments: Reviewer #2

In this paper, Eekhout et al., systematically compare protoplast enrichment methods (conventional and image-based flow cytometry, magnetic cell sorting) together with two scRNA-seq platforms (10x Genomics Chromium and BD Rhapsody) using Arabidopsis roots. They report that image-based flow cytometry enables precise cell selection, while magnetic sorting better preserves cell size heterogeneity. Both scRNA-seq platforms captured root cell diversity with reproducible gene expression, but platform-specific biases in cell type composition were observed. Furthermore, SNP analysis revealed that a substantial fraction of doublets may be misclassified, providing important guidance for optimizing scRNA-seq workflows in plants. This manuscript is technically interesting and important for guiding plant single-cell research, and it has the potential to attract a broad readership. However, I have several concerns and comments listed below.

=> We thank the reviewer for the supporting comments on the importance and timeliness of our manuscript; as well as the insightful comments which helped to further improve our manuscript.

Major concerns and comments:

1a. One key limitation of the comparison between the 10x Genomics and BD Rhapsody platforms (Figure 2A-H) is the absence of statistical analysis across the biological replicates. Without such analysis, it is difficult to fully support the conclusions such as that BD achieves higher cell retention. Technical variability-for example, differences in protoplast handling during pipetting-can introduce substantial variation even within the same platform. This issue is evident in the authors' own data, where BD replicate-2 disproportionately contributes to cluster 24 in Figure 3C compared to BD replicate-1. To strengthen the conclusions, appropriate statistical tests should be performed on the biological replicates.

=> We agree that statistical comparisons across biological replicates would, in principle, provide a stronger basis for comparing platform performance. However, in the present study, only two biological replicates per platform were generated, which is insufficient for robust formal statistical testing of differences between platforms or of cluster representation across replicates. After consulting with expert statisticians, we concluded that standard inferential approaches (e.g. mixed-effects models or permutation-based tests) would not provide reliable P-values or confidence intervals with this limited number of replicates, and could give a misleading impression of precision. Because of this constraint, we have refrained from performing formal statistical tests on replicate-level metrics such as cell retention or cluster proportions, and we have revised the text to describe these differences as qualitative trends rather than statistically supported effects. Instead, to assess reproducibility and support the robustness of our conclusions as far as possible, we have:

- repeated experiments and included additional biological replicates where feasible for cell viability and size heterogeneity (**Fig 1**);
- performed correlation and permutation analyses to examine the consistency of platform-specific gene detection and sensitivity across replicates and sequencing depths (**Fig 2I-3D, Appendix Figure S1C**);
- systematically compared multiple batch-correction and doublet-detection workflows and confirmed that our main conclusions are stable with respect to these processing choices (**Fig EV2, Appendix Figure S3, Fig EV5**).

1b. In addition, when comparing different cell cleaning methods, the authors should clarify how the "three technical replicates" described in the Methods (line 427) were defined.

=> We thank the referee for this suggestion. We have repeated the experiment and added the new data to Figure 1. In the revised manuscript, we have also clarified that the results were obtained from biologically independent experiments, rather than technical replicates (see line 514-515): "*The data shown in Figure 1 summarize two biologically independent experiments.*"

2. Second, it would be helpful to check the GO enrichment or determine what kinds of genes are represented among the unique genes captured by either the BD or 10x platform. For example, do the 823 unique genes found by 10x Chromium belong to categories such as pseudogenes, ribosomal genes, or certain non-coding RNA genes that are excluded by BD when generating the reference genome?

=> We had to correct the numbers of genes provided in this Figure. We did not observe an enrichment of pseudogenes, ribosomal RNA, or specific non-coding RNA classes, but calculated the proportion of cells in which the technology-specific gene was detected in order to estimate their abundance (Table EV4). The majority of technology-specific genes were found in less than 0.05% of all cells (~25 cells; see figure below).

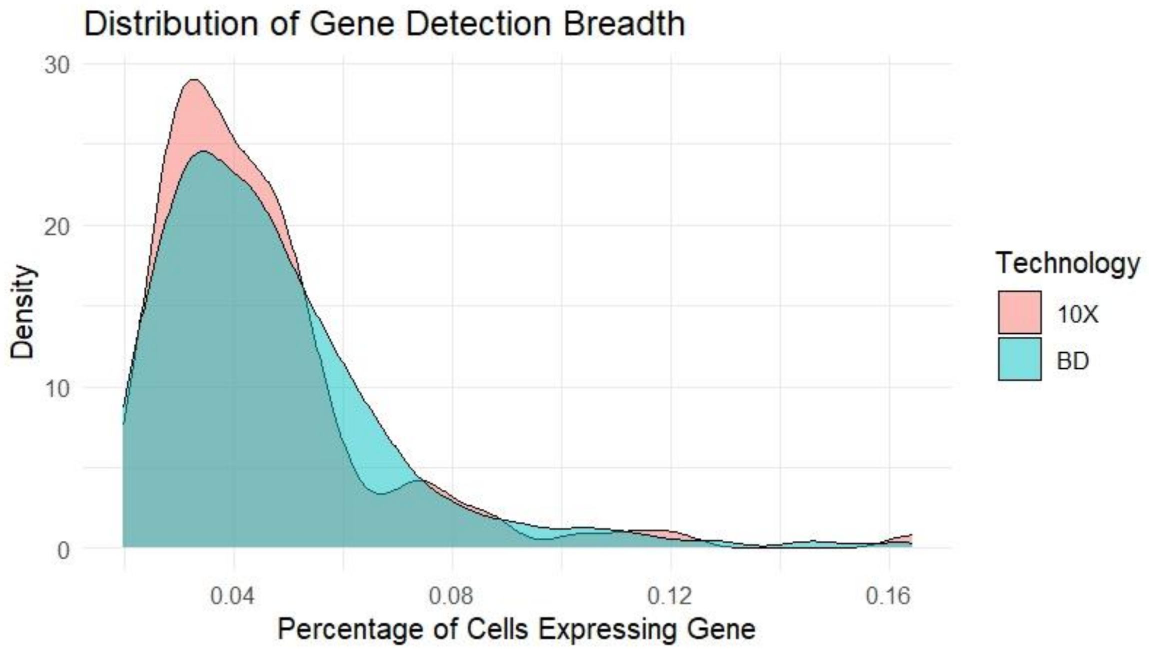

We found that 30% of the 10X-specific genes and 37% of the BD-specific genes were expressed in more than 25 cells, indicating that a substantial subset of platform-specific genes exhibits relatively broad expression rather than being confined to a few rare cells. We also performed an additional GO term enrichment analysis. While no GO category reached statistical significance after multiple testing correction for the 10X-specific genes, BD-specific genes showed significant enrichment for "RNA modification" ($p = 0.03$), including several Pentatricopeptide repeat (PPR) proteins, and for "protein binding" ($p = 1\times10^{-4}$), encompassing, for example, *SCARECROW-LIKE 28*, *MYB101*, and *WUSCHEL*. These results are now included in the revised manuscript (line 159-176): "*For 10X Chromium, the two replicates shared 105 uniquely detected genes, with an additional 174 and 123 uniquely detected genes in replicates 1 and 2, respectively (**Table EV4**). For BD Rhapsody, the replicates shared 671 uniquely detected genes, with an additional 284 and 495 genes*

uniquely detected in replicates 1 and 2, respectively. 30% of 10x-specific and 37% of BD-specific genes were expressed in more than 25 cells, indicating that many platform-specific genes are not restricted to a few rare cells but show relatively broad expression. No GO category reached statistical significance for the 10x-specific genes, whereas BD-specific genes showed significant enrichment for 'RNA modification' (p = 0.03), including several Pentatricopeptide repeat (PPR) proteins, and for 'protein binding' (p = 1×10^{-4}), including, for example, SCARECROW-LIKE 28, MYB101, and WUSCHEL. The overall number of platform-specific genes was, however, compatible with random sampling variation rather than a strong, systematic platform bias in gene detection. These platform-specific gene sets are therefore best interpreted as reflecting subtle technical distinctions between BD Rhapsody and 10X Chromium (e.g. differences in cell/RNA capture, library preparation, and sensitivity to low-expression genes), as well as inherent variability in transcript recovery across cell types. Given the limited number of biological replicates (two per platform), we did not attempt formal between-replicate statistical testing."

3. Third, in line 215-221, the authors propose that the BD Rhapsody system may preferentially capture cells in specific biological states-such as those exhibiting high translational activity or stress responses-thereby contributing to increased cellular heterogeneity within clusters. However, this interpretation is not supported by the design or functionality of the BD Rhapsody platform. During cell capture, the system does not assess or select cells based on transcriptional or translational activity; rather, it captures intact, viable cells without regard to their underlying molecular profiles. Therefore, the suggestion that BD Rhapsody selectively enriches for certain biological states is mechanistically implausible. This portion of the discussion should be revised to avoid overstating the implications of the observed gene set enrichment.

=> We thank the referee for pointing out this oversight and agree that our original phrasing may have overstated the implications. We acknowledge that the BD Rhapsody platform does not selectively capture cells based on transcriptional or translational activity. Unlike platforms that allow user-directed selection (e.g., iCell8 or Takara Shasta), BD Rhapsody captures intact, viable cells without bias toward specific molecular states. Accordingly, we have revised the passage in the manuscript (line 257-261) to read: "*These patterns could reflect differences in cellular stress or viability during sample processing and/or a comparatively better performance of BD Rhapsody in capturing and lysing cells in specific biological states. However, because neither platform permits selective processing and unambiguous tracing of individual cells, we cannot definitively discriminate between these possibilities.*"; hereby removing any suggestion of active selection by the platform.

4. Fourth, the observation that BD Rhapsody yielded a higher proportion of epidermis, initials, and lateral root cap cells, while 10x Chromium enriched for cortex and endodermis cells, is very interesting and suggests a platform-dependent bias in cell type recovery. One possible explanation is a difference in cell size that affects capture efficiency between the microwell-based BD Rhapsody system and the droplet-based 10x Chromium platform. To test this hypothesis, the authors could use cell type-specific promoter reporter lines to visualize and quantify cell sizes across these populations. This would provide direct evidence as to whether size differences contribute to the observed capture bias and help clarify whether physical properties of cells influence platform-specific representation.

=> To test whether cell size contributes to platform-dependent differences in cell type recovery, we performed magnetic-levitation enrichment on cell type–specific reporter lines (p*SCR*::GFP for endodermis, p*SMB*::NLS-GFP for lateral root cap, p*CO2*::CO2-GFP for cortex, and p*WER*::WER-GFP for epidermis) and quantified cell morphology using our Opera imaging setup (Appendix Figure S4, Table EV6). Mean cell diameters were similar for endodermis (15.6 µm), epidermis (15.3 µm), and lateral root cap cells (15.2 µm), and slightly larger for cortex cells (16.4 µm). Lateral root cap and cortex cells both exhibited a broader size range compared with endodermis and epidermis. Cortex cells are enriched in 10X Chromium, whereas lateral root cap cells are enriched in BD Rhapsody, indicating that platform-specific cell type biases cannot be solely explained by differences in average cell size or size heterogeneity. This analysis supports the view that additional factors beyond cell size, such as platform-specific capture mechanics or cell lysis efficiency, likely contribute to the observed differences in cell type representation. We included these additional analyses and interpretations in the revised manuscript (line 270-274): "*Morphology analysis of cells from epidermis, endodermis, cortex, and lateral root cap reporter lines showed that both lateral root cap and cortex cells exhibit a broader size range than endodermis and epidermis (**Appendix Figure S4; Table EV6**), indicating that cell size limitations cannot be the sole explanation for platform-dependent differences in capture efficiency.*"

Minor comments:

1. Line 319. The authors state that flow cytometry substantially enhanced cell viability. I disagree with this interpretation, as the main function of flow cytometry is to remove debris and dead cells rather than directly improving viability. Please rephrase this statement accordingly.

=> We fully agree and have thus followed the referee´s advice and adapted the statement in the current manuscript into (line 383-385): "*While flow cytometry helped in achieving a higher sample purity, magnetic levitation increased the proportion of viable cells and outperformed flow cytometry in maintaining the original size heterogeneity of viable protoplasts **(Fig** 1).*"

2. In Figure 3B, it would be helpful to include a UMAP of cells grouped by different replicates of 10x and BD after batch correction to demonstrate that the Harmony algorithm effectively removed the batch effect.

=> We thank the referee for the suggestion. To demonstrate that the Harmony algorithm effectively removed batch effects, we have included a UMAP of cells colored by replicate for both 10X Chromium and BD Rhapsody after batch correction. This is now presented in Figure 3A as well.

3. An estimated 20,000 cells from the same protoplast sample were simultaneously loaded as input on both the 10x Chromium (10x) and BD Rhapsody (BD) platforms. However, this does not account for potential differences in recovery rate between the two platforms. If the recovery rates were not the same, then sequencing each library to a similar depth of 150 million reads could result in variation in sequencing depth per cell.

=> We thank the referee for raising this important point. The estimated recovery rates for 10X Chromium and BD Rhapsody are 40-65% and 65-75%, respectively. Given the similarity in recovery rates, we can reasonably assume comparable capture efficiencies and thus similar

sequencing depth per cell when sequencing each library to ~150 million reads. To further validate this assumption, we performed a drop-out analysis in which reads were subsampled and key metrics—such as genes per cell and UMIs per cell—were correlated across platforms. This analysis is now included as Appendix Figure S1, and the conclusions are summarized in the revised manuscript (line 183-193): "*These differences might suggest greater transcript capture sensitivity for 10X Chromium but can also be partly explained by the higher sequencing depth per cell, as fewer cells were captured while both samples were sequenced at 150M reads. To assess robustness to sequencing depth, we subsampled each dataset to 50M, 100M, and 150M reads per sample and repeated the correlation analyses (**Appendix Figure S1**). Genes per cell and UMIs per cell remained consistent across depths for both platforms (**Appendix Figure S1A-B**), supporting comparable capture efficiency at matched sequencing depth. The fraction of genes detected per technology remained highly correlated across depths (**Appendix Figure S1C**; 50M: Pearson 0.877, Spearman 0.941; 100M: Pearson 0.892, Spearman 0.944; 150M: Pearson 0.899, Spearman 0.946), indicating that the increased transcript capture sensitivity for 10X is not solely driven by unequal sequencing depth or cell numbers.*" We hope this additional analysis resolves this issue.

4. How do the two platforms compare in their ability to capture quiescent center cells? Given their low abundance, even small differences in recovery efficiency between 10X Chromium and BD Rhapsody could lead to significant discrepancies in representation. It would be helpful if the authors could comment on whether such rare populations were differentially captured or detected across the platforms.

=> We thank the referee for raising this point. In our experiments, quiescent center (QC) cells were not captured among the four replicates, likely due to a combination of factors, such as duration of enzymatic tissue digestion, their extremely low abundance, and the absence of a precise marker gene set for unambiguous identification. Detecting such rare populations typically requires optimized duration of the tissue digestion and a pre-enrichment strategies such as FACS would be more appropriate for targeted recovery. Nevertheless, we do detect marker genes associated with other low-abundance root cell types on both platforms (see Figure below). For example, the columella marker *DRO3* (*AT1G17400*) and the xylem pole pericycle marker *AT4G30450* are expressed in both datasets, as are the transcription factors *WIP2* (*AT3G57670*) and *WIP3* (*AT1G08290*), which participate in root cell fate regulation. For example, *WIP2* is detected in 14 (0.44%) 10X cells versus 37 (0.14%) BD cells, *WIP3* in 82 (2.6%) versus 76 (0.28%), *DRO3* in 17 (0.54%) versus 23 (0.08%), and *AT4G30450* in 181 (5.7%) versus 92 (0.34%) cells for 10X and BD, respectively. These values illustrate that both platforms capture markers of low-abundance or spatially restricted

populations, albeit with platform-specific differences in detection frequency.

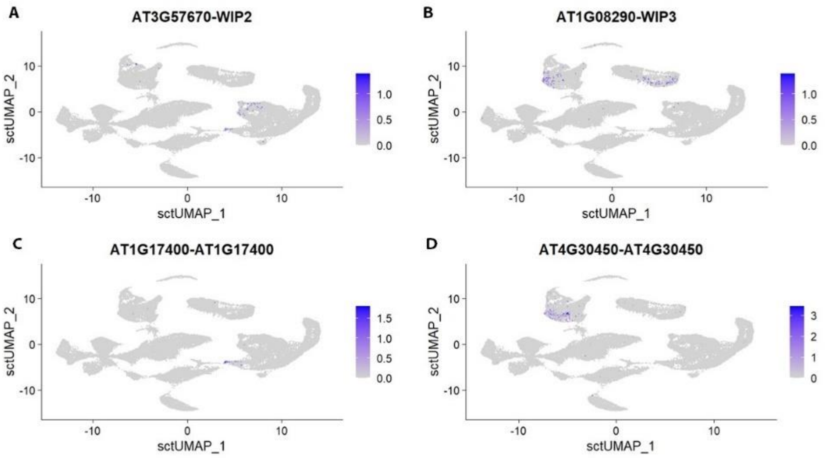

The revised manuscript now contains a notion to this aspect (line 412-415): "*Both platforms captured all major cell types described in Arabidopsis roots, except for quiescent center (QC) cells, which are often underrepresented in scRNA-seq studies due to several factors, including their natural rarity, suboptimal enzymatic digestion conditions, and difficulties in unambiguous identification based on marker genes alone.*"

5. An estimated 20,000 cells... → typo: should be 20,000 cells

=> We modified the sentence in the resubmitted manuscript.

Reply to Reviewer Comments: Reviewer #4

Review: Performance evaluation of Arabidopsis scRNA-seq sample processing Strategies
The authors work to optimise the workflow of single cell sequencing methodologies, from the method of cell isolation through to the integration and analyses of the cell populations at the bioinformatic level. This paper is of interest to people working with this single cell transcriptomics in plant materials - a relatively small but growing community. I think the paper offers some valuable insights that can help guide people through this challenging and expensive experimental approach. I think that the authors set the scene with an appropriate level of background information and clearly explain the reasoning for putting together this body of work. I do however think that this manuscript lacks robust conclusions and contains a high level of speculative and unclear interpretations. I am also concerned by the lack of statistical analyses reported throughout. While I make comments relating to specific parts of the manuscript below, these can be read as general comments as I observed the same issues throughout.

=> We thank the referee for these general observations regarding the clarity and robustness of our conclusions. In the revised manuscript, we have strengthened our conclusions by clarifying interpretations, adding additional biological replicates where feasible (Figure 1), and included correlation and permutation tests to strengthen our conclusions on the effect of sequencing depth on capture sensitivity (Appendix Figure S1C), platform-specific gene capture and replicate reproducibility (Figure 2I), and benchmarked three tools for data integration and doublet identification (Figure EV2, Appendix Figure S3, Figure EV5). We note, however, that additional statistical comparisons across single-cell datasets are not possible in our study because only two replicates per technology were generated, which limits formal statistical testing across platforms. Nonetheless, we utilized correlation-based reproducibility assessments and included additional analyses with multiple computational tools to ensure that the reported data and conclusion are robust. We believe that these revisions substantially improve the rigor and clarity of the manuscript, addressing the referee's concerns about robustness and statistical support.

1) I think it was valuable to assess the degree of cell size heterogeneity maintained across cell capture methods compared to unsorted samples. It seems clear that Levitation is better for capturing the heterogeneity that exists in the root tissue. It would be good to see replication here, however.

=> We thank the referee for highlighting the importance of assessing cell size heterogeneity across capture methods. In response, we have added an additional independent replicate of the levitation and flow cytometry experiments. These replicates confirm that magnetic levitation consistently preserves the full spectrum of cell sizes in root tissue, whereas flow cytometry tends to underrepresent larger cells. These additional experiments strengthen our conclusions regarding the advantage of levitation for maintaining cell size heterogeneity and have been included in Figure 1 and the revised Results section (line 109-128): "*After cell sorting viability increased to 94% and 87% for image-based and regular flow cytometry, respectively, compared to 51% viability in the non-purified sample (**Fig 1D**). Viability after magnetic levitation reached 72%. To estimate the effect of flow cytometry versus magnetic levitation-based cell separation on preserving the original cell size heterogeneity among root cells, we quantified protoplast size (defined as cell diameter) distributions after applying different enrichment strategies (**Fig 1E**). Magnetic levitation outperformed both flow cytometers in*

*maintaining the original size heterogeneity of viable protoplasts (**Table EV2**): viable Arabidopsis root cells purified by magnetic separation displayed a broad and representative range of cell sizes (mean cell diameter=12.6 µm; cells larger than 20 µm accounted for 7% of all analyzed cells), comparable to the unsorted sample (mean cell diameter=14 µm; cells larger than 20 µm accounted for 12% of all analyzed cells), whereas both flow cytometers yielded a narrower size range biased toward smaller cells (mean cell diameter= 10.3/11.6; cells larger than 20 µm accounted for only 2% of the population). In unsorted samples and after magnetic levitation, viable and dead cells showed similar cell diameter distribution and roundness (**Fig EV1A–C**), with no correlation between these parameters (**Fig EV1D**). After image-based flow cytometry sorting, alive cells were smaller than dead cells, and dead cells showed reduced roundness (**Fig EV1A–C**). Because no differences were observed in the unpurified samples, and diameter and roundness were uncorrelated in all conditions (**Fig EV1D**), these changes likely result from shearing forces during sorting rather than pre-existing large or irregular aggregates."*

2) The authors state the high importance of cell viability and then opt to use a method with a 20% lower cell viability count. I can see arguments for prioritizing cell size heterogeneity over viability, but the authors should explain their rationale here. For me viability seems like the lesser evil with the possibility to remove low quality cells at the QC level bioinformatically.

=> We thank the referee for raising this important point. While we acknowledge that cell viability is critical, in our study we prioritized sample purity and reduction of debris over absolute viability. The rationale is twofold: 1.) Minimizing debris reduces the risk of chip clogging during single-cell capture (particularly for the 10X Chromium platform), which can otherwise lead to substantial sample loss. 2.) Improved signal-to-noise ratio: samples with lower debris content provide clearer transcriptomic signals, making downstream analyses more reliable. Low-quality or non-viable cells can still be removed at the bioinformatics QC stage, allowing us to retain the advantages of higher purity without compromising data quality. This rationale has now been clarified in the revised manuscript (lines 139–141): "*We prioritized sample purity over absolute cell viability, as reduced debris lowers the risk of chip clogging and improves signal-to-noise ratio, while low-quality cells can subsequently be removed during bioinformatic quality control filtering*"

3) I find Figure 1 panel B confusing. I made sense of it, but the keys used are unnecessary. Maybe consider a proportion/stacked bar chart per technology for example.

=> We thank the referee for the suggestion regarding Figure 1B. Figure 1B is intended to show a snapshot of the levitation process, including the individual channels (Calcein, PI, Bright Field), and we believe the current presentation is the most informative for this purpose. We assume that the referee's comment may have been intended for Figure 1D. Following this suggestion, we have revised Figure 1D to use a proportion/stacked bar chart per technology, which improves clarity and better conveys the distribution of cell sizes across platforms.

4) I find the comparisons made useful in many cases but many of the conclusions are too vague or speculative and offer no guidance for future researchers.

=> We thank the referee for this observation and agree that providing actionable guidance for future researchers is an important goal of this study. We now explicitly highlight implications

for experimental design—including considerations for platform selection, sample preparation, and expected trade-offs such as cell yield versus size heterogeneity—which are summarized in Table 1 and referenced in both the Results and Discussion. At the same time, we deliberately refrain from making broad generalizations about the performance of these technologies across all plant species and tissues, because specific biological questions, tissue properties, and input quality can strongly influence platform performance and sensitivity, and must therefore be carefully considered in the experimental setup and data analysis.

5) The venn diagram in Figure 2 shows the overlap of genes identified in each platform and replicate. The authors state that " BD Rhapsody exclusively detecting 791 genes that were not captured by 10X Chromium, while 10X Chromium uniquely detected 823 genes not found in BD Rhapsody samples." The "791" however is just for one replicate of BD and I cannot see where the"823" comes from for 10x. I can only see 823 for the second BD replicate.

=> The referee is correct that the originally reported values were inconsistent between the text and the figure. We repeated the gene count and identified a mistake in our previous figure. We have thus now corrected the figure in the revised manuscript and ensured that the numbers now correspond exactly to the gene counts displayed in the Venn diagram (see line 159-176): "*For 10X Chromium, the two replicates shared 105 uniquely detected genes, with an additional 174 and 123 uniquely detected genes in replicates 1 and 2, respectively (**Fig 2B**; **Table EV4**). For BD Rhapsody, the replicates shared 671 uniquely detected genes, with an additional 284 and 495 genes uniquely detected in replicates 1 and 2, respectively. 30% of 10X-specific and 37% of BD-specific genes were expressed in more than 25 cells, indicating that many platform-specific genes are not restricted to a few rare cells but show relatively broad expression. No GO category reached statistical significance for the 10X-specific genes, whereas BD-specific genes showed significant enrichment for 'RNA modification' (p = 0.03), including several Pentatricopeptide repeat (PPR) proteins, and for 'protein binding' ($p = 1\times10^{-4}$), including, for example, SCARECROW-LIKE 28, MYB101, and WUSCHEL. The overall number of platform-specific genes was, however, compatible with random sampling variation rather than a strong, systematic platform bias in gene detection. These platform-specific gene sets are therefore best interpreted as reflecting subtle technical distinctions between BD Rhapsody and 10X Chromium (e.g. differences in cell/RNA capture, library preparation, and sensitivity to low-expression genes), as well as inherent variability in transcript recovery across cell types. Given the limited number of biological replicates (two per platform), we did not attempt formal between-replicate statistical testing.*"

Overall, while I think this study could in the future be of help to people planning these difficult experiments, the power of this study has not yet been realised.

=> We thank the referee for this thoughtful overall assessment. We agree that the potential impact of this study lies in providing guidance for researchers planning technically challenging single-cell experiments, and we appreciate the opportunity to further strengthen the manuscript. Given the complete absence of benchmark studies in the plant field, we do believe we are providing an important (but admittedly not perfect) contribution to the field. Still, in response to the referee's comments, we have repeated several experiments and expanded our analyses to more directly address issues of cell size, viability, gene detection, platform comparison and cluster and doublet detection. We have also clarified methodological details, improved figure presentation, and added critical discussion on the scope and limitations of our work. While we acknowledge that additional lines of investigation (e.g.,

transcriptomic profiling of large-cell subsets or the use of alternative single-cell platforms) would further expand the study, we believe that the revisions substantially enhance both the rigor and the utility of the manuscript. We thus feel that, in its current form, the study now provides a solid and informative resource for the community as we had originally intended.

Dear Dr. Grones,

We have now received re-review reports from all three referees, which I have included below. As you will see, you have addressed their concerns satisfactorily; however, I would like you to consider the suggestions of Reviewer #2 regarding the strength of the language with which you claim platform-specific biases. Before I can finally accept the manuscript, there are some remaining editorial points which need to be addressed. In this regard would you please:

- remove figures from the main manuscript, but leave the figure legends and the EV figure legends at the end,
- ensure names are identical in the submission system and on the main manuscript (Caroline Grones/Freya Persyn (ms) vs Caroline Grones Seyfferth/Persyn Freya (system),
- remove the author credit section from the text,
- upload EV tables as Dataset EV1, EV2 etc.; also adjust the nomenclature in the file name, caption and manuscript callouts,
- include a title page for the appendix saying 'Appendix for Performance evaluation of Arabidopsis scRNA-seq sample processing strategies' and a Table of Contents with each item listed (figures and tables) with its page number,
- relabel the 'Structured Methods' section as 'Methods',
- use the following section order: Title page - Abstract & Keywords - Introduction - Results - Discussion - Methods - Data Availability - Acknowledgments - Disclosure and Competing Interests Statement - References - Figure Legends - (Main Tables with legends if applicable) - Expanded View Figure Legends,
- remove captions for EV tables (to be Datasets) from the manuscript; they should only remain in the file itself (on a separate sheet),
- define box plots in terms of minima, maxima, centre, bounds of box and whiskers, and percentile in the legends of figures EV1 A, C; EV3 B, and
- define the nature of 'n' in the legends of figures 2G, H; 4C, D; EV3 B.

We include a synopsis of the paper (see http://emboj.embopress.org/). Please provide me with a general summary image, a two sentence statement and 3-5 bullet points that capture the key findings of the paper.

I am looking forward to receiving your revised manuscript.

EMBO Press is an editorially independent publishing platform for the development of EMBO scientific publications.

Best wishes,

William

William Teale, PhD
Editor
The EMBO Journal
w.teale@embojournal.org

Instructions for preparing your revised manuscript:

Read our guidance for manuscript revisions and related editorial policies: https://link.springer.com/journal/44318/submission-guidelines#cms-Revised-submissions

Please also check that the title and abstract of the manuscript are brief, yet explicit, even to non-specialists.

When assembling figures, please refer to our figure preparation guideline in order to ensure proper formatting and readability in print as well as on screen:
https://media.springernature.com/original/springer-cms/rest/v1/content/27825798/data/v1

IMPORTANT: When you send the revision we will require
- a point-by-point response to the referees' comments, with a detailed description of the changes made (as a word file).
- a word file of the manuscript text.
- individual production quality figure files (one file per figure)
- a complete author checklist
- Expanded View files (replacing Supplementary Information)
- a Reagents and Tools Table as part of the Methods section

Please remember: Digital image enhancement is acceptable practice, as long as it accurately represents the original data and conforms to community standards. If a figure has been subjected to significant electronic manipulation, this must be noted in the figure legend or in the 'Methods' section. The editors reserve the right to request original versions of figures and the original images that were used to assemble the figure.

We realize that it is difficult to revise to a specific deadline. In the interest of protecting the conceptual advance provided by the work, we recommend a revision within 3 months (17th Jun 2026). Please discuss the revision progress ahead of this time with the editor if you require more time to complete the revisions. Use the link below to submit your revision:

https://emboj.msubmit.net/cgi-bin/main.plex

Referee #1:

We thank the authors for their thorough revision. All our initial major concerns have been addressed.

We appreciate the extended efforts put on additional analyses on cell size and viability, and the deeper correlation analyses to show the robustness across batch correction and doublet detection workflows. The expanded Methods section also helps improve transparency and reproducibility.

We only suggest ensuring that the legend of Figure 1A clearly explains all schematic elements for standalone readability.

Overall, we consider that the manuscript is now suitable for publication in its current form.

Referee #2:

In this revision, the authors have addressed most of my concerns. However, I remain unconvinced that the current data provide sufficient statistical support for the conclusion that they "observed platform-specific biases in cell type composition," as stated in the Abstract (lines 31-32).

The revised Results section provides additional evidence that the two platforms produce different clustering patterns and different apparent proportions of annotated cell types. Nevertheless, I do not believe that the current study design justifies the stronger claim of "platform-specific biases in cell type representation/composition" as a firm conclusion. With only two biological replicates per platform, and with at least some cluster differences appearing replicate-specific (e.g. cluster 24), these observations remain primarily descriptive and cannot yet be cleanly distinguished from sample-processing effects or replicate-level variation. I therefore recommend that the authors soften the language throughout the manuscript, including in the Abstract, by referring instead to "platform-associated" or "apparent" differences in cell type representation/composition, unless additional replicate-level evidence can be provided.

Referee #4:

The authors have adressed all of my concerns

Reply to Reviewer Comments: Reviewer #1

Referee #1:

We thank the authors for their thorough revision. All our initial major concerns have been addressed.

We appreciate the extended efforts put on additional analyses on cell size and viability, and the deeper correlation analyses to show the robustness across batch correction and doublet detection workflows. The expanded Methods section also helps improve transparency and reproducibility.

We only suggest ensuring that the legend of Figure 1A clearly explains all schematic elements for standalone readability.

Overall, we consider that the manuscript is now suitable for publication in its current form.

=> We thank the reviewer for the positive evaluation and supportive comments. We are pleased that the additional analyses and methodological clarifications addressed the initial concerns.

As suggested, we have revised the legend of Figure 1A to ensure that all schematic elements are clearly explained and that the figure can be understood independently (Line 851-865): "*Gating strategy used to enrich alive cells with image-based flow cytometry (BD FACSDiscover S8). The left panel ("All cells") shows all events in a dot plot of side scatter (SSC, y-axis) versus LightLoss(Violet) (Lightloss(Violet)-A, x-axis). Lightloss is an image-derived parameter that measures the reduction in incident laser light caused by scattering and absorption as the cell passes the laser beam at the interrogation point (Schraivogel et al. 2022). It serves as a proxy to forward scatter but provides improved definition on the boundary of the cell. The polygon gate selects intact cells based on size and granularity, excluding smaller debris. The middle panel ("Single, round cells") displays events from this gate plotted by image-derived eccentricity (y-axis) versus image-derived cell size (x-axis); the P1 gate enriches round, single cells by excluding doublets and aggregates, which show higher eccentricity values (greater than 0.6). Within the P1 population, alive single cells (light blue) are identified by high Calcein AM fluorescence intensity and confirmed by corresponding cell images (panel "Single cells (alive)"), while dead single cells (purple) are identified by high Propidium Iodide (PI) fluorescence intensity and likewise confirmed by images (panel "Single cells (dead)").*"

We appreciate the reviewer's time and constructive feedback throughout the revision process.

Reply to Reviewer Comments: Reviewer #2

Referee #2:

In this revision, the authors have addressed most of my concerns. However, I remain unconvinced that the current data provide sufficient statistical support for the conclusion that they "observed platform-specific biases in cell type composition," as stated in the Abstract (lines 31-32).

The revised Results section provides additional evidence that the two platforms produce different clustering patterns and different apparent proportions of annotated cell types. Nevertheless, I do not believe that the current study design justifies the stronger claim of "platform-specific biases in cell type representation/composition" as a firm conclusion. With only two biological replicates per platform, and with at least some cluster differences appearing replicate-specific (e.g. cluster 24), these observations remain primarily descriptive and cannot yet be cleanly distinguished from sample-processing effects or replicate-level variation. I therefore recommend that the authors soften the language throughout the manuscript, including in the Abstract, by referring instead to "platform-associated" or "apparent" differences in cell type representation/composition, unless additional replicate-level evidence can be provided.

=> We thank the reviewer for this constructive and thoughtful comment. We agree that, given the limited number of biological replicates the conclusions should be framed more cautiously.

Accordingly, we have revised the manuscript throughout, replacing all instances of “platform-specific” with “platform-associated” and adding “apparent” where appropriate to better reflect the descriptive nature of these observations. These changes are highlighted in the newly submitted version of the manuscript.

We appreciate the reviewer’s time and constructive feedback provided throughout the revision process.

Reply to Reviewer Comments: Reviewer #4

Referee #4:

The authors have adressed all of my concerns.

=> We thank the reviewer for the positive evaluation and for confirming that all concerns have been addressed. We appreciate the time and constructive feedback provided throughout the revision process.

2nd Revision - Editorial Decision 22nd Apr 2026

Dear Carolin,

I am pleased to inform you that your manuscript has been accepted for publication in the EMBO Journal.

Congratulations to you and all involved!

Your manuscript will be processed for publication by EMBO Press. It will be copy edited and you will receive page proofs prior to publication. Please note that you will be contacted by Springer Nature Author Services to complete licensing and payment information.

You may qualify for financial assistance for your publication charges - either via a Springer Nature fully open access agreement or an EMBO initiative. Check your eligibility: https://link.springer.com/journal/44318/how-to-publish-with-us

Should you be planning a Press Release on your article, please get in contact with embo_production@springernature.com as early as possible in order to coordinate publication and release dates.

If you have any questions, please do not hesitate to contact the Editorial Office. Thank you for your contribution to The EMBO Journal.

Best wishes,

William,

William Teale, PhD
Editor
The EMBO Journal
w.teale@embojournal.org

*****
Please note that it is The EMBO Journal policy for the transcript of the editorial process (containing referee reports and your response letters) to be published as an online supplement to each paper. If you should prefer removal of any referee-only figures included in the point-by-point response(s), e.g. because they may still be used for future publication or because they have been reproduced from published work by others, please do let us know immediately via response email.
More information is available here: https://link.springer.com/partners/embo-press/editorial-policies#Peer%20review

** Click here to be directed to your login page: https://emboj.msubmit.net

EMBO Press Author Checklist

Corresponding Author Name: Bert De Rybel, Carolin Grones
Journal Submitted to: The EMBO Journal
Manuscript Number: EMBOJ-2025-121847

USEFUL LINKS FOR COMPLETING THIS FORM
The EMBO Journal - Author Guidelines
EMBO Reports - Author Guidelines
Molecular Systems Biology - Author Guidelines
EMBO Molecular Medicine - Author Guidelines

Reporting Checklist for Life Science Articles (updated January
This checklist is adapted from Materials Design Analysis Reporting (MDAR) Checklist for Authors. MDAR establishes a minimum set of requirements in transparent reporting in the life sciences (see Statement of Task: 10.31222/osf.io/9sm4x). Please follow the journal's guidelines in preparing your
Please note that a copy of this checklist will be published alongside your article.

Abridged guidelines for figures
1. Data
The data shown in figures should satisfy the following conditions:
- the data were obtained and processed according to the field's best practice and are presented to reflect the results of the experiments in an accurate and unbiased manner.
- ideally, figure panels should include only measurements that are directly comparable to each other and obtained with the same assay.
- plots include clearly labeled error bars for independent experiments and sample sizes. Unless justified, error bars should not be shown for technical
- if n<5, the individual data points from each experiment should be plotted. Any statistical test employed should be justified.
- Source Data should be included to report the data underlying figures according to the guidelines set out in the authorship guidelines on Data

2. Captions
Each figure caption should contain the following information, for each panel where they are relevant:
- a specification of the experimental system investigated (eg cell line, species name).
- the assay(s) and method(s) used to carry out the reported observations and measurements.
- an explicit mention of the biological and chemical entity(ies) that are being measured.
- an explicit mention of the biological and chemical entity(ies) that are altered/varied/perturbed in a controlled manner.
- the exact sample size (n) for each experimental group/condition, given as a number, not a range;
- a description of the sample collection allowing the reader to understand whether the samples represent technical or biological replicates (including how many animals, litters, cultures, etc.).
- a statement of how many times the experiment shown was independently replicated in the laboratory.
- definitions of statistical methods and measures:
  - common tests, such as t-test (please specify whether paired vs. unpaired), simple χ2 tests, Wilcoxon and Mann-Whitney tests, can be unambiguously identified by name only, but more complex techniques should be described in the methods section;
  - are tests one-sided or two-sided?
  - are there adjustments for multiple comparisons?
  - exact statistical test results, e.g., P values = x but not P values < x;
  - definition of 'center values' as median or average;
  - definition of error bars as s.d. or s.e.m.

Please complete ALL of the questions below.
Select "Not Applicable" only when the requested information is not relevant for your study.

Materials

Newly Created Materials	Information included in the manuscript?	In which section is the information available? (Reagents and Tools Table, Materials and Methods, Figures, Data Availability Section)
New materials and reagents need to be available; do any restrictions apply?	Not Applicable	

Antibodies	Information included in the manuscript?	In which section is the information available? (Reagents and Tools Table, Materials and Methods, Figures, Data Availability Section)
For **antibodies** provide the following information:<br>- Commercial antibodies: RRID (if possible) or supplier name, catalogue number and or/clone number<br>- Non-commercial: RRID or citation	Not Applicable	

DNA and RNA sequences	Information included in the manuscript?	In which section is the information available? (Reagents and Tools Table, Materials and Methods, Figures, Data Availability Section)
**Short novel DNA or RNA including primers, probes**: provide the sequences.	Not Applicable	

Cell materials	Information included in the manuscript?	In which section is the information available? (Reagents and Tools Table, Materials and Methods, Figures, Data Availability Section)
**Cell lines:** Provide species information, strain. Provide accession number in repository **OR** supplier name, catalog number, clone number, and/**OR** RRID.	Not Applicable	
**Primary cultures:** Provide species, strain, sex of origin, genetic modification status.	Not Applicable	
Report if the cell lines were recently **authenticated** (e.g., by STR profiling) and tested for mycoplasma contamination.	Not Applicable	

Experimental animals	Information included in the manuscript?	In which section is the information available? (Reagents and Tools Table, Materials and Methods, Figures, Data Availability Section)
**Laboratory animals or Model organisms:** Provide species, strain, sex, age, genetic modification status. Provide accession number in repository **OR** supplier name, catalog number, clone number, **OR** RRID.	Not Applicable	
**Animal observed in or captured from the field:** Provide species, sex, and age where possible.	Not Applicable	
Please detail **housing and husbandry conditions**.	Not Applicable	

Plants and microbes	Information included in the manuscript?	In which section is the information available? (Reagents and Tools Table, Materials and Methods, Figures, Data Availability Section)
**Plants:** provide species and strain, ecotype and cultivar where relevant, unique accession number if available, and source (including location for collected wild specimens).	Yes	Materials and Methods
**Microbes:** provide species and strain, unique accession number if available, and source.	Not Applicable	

Human research participants	Information included in the manuscript?	In which section is the information available? (Reagents and Tools Table, Materials and Methods, Figures, Data Availability Section)
If collected and within the bounds of privacy constraints report on age, sex and gender or ethnicity for all study participants.	Not Applicable	

Core facilities	Information included in the manuscript?	In which section is the information available? (Reagents and Tools Table, Materials and Methods, Figures, Data Availability Section)
If your work benefited from core facilities, was their service mentioned in the acknowledgments section?	Yes	Acknowledgments

Design

Study protocol	Information included in the manuscript?	In which section is the information available? (Reagents and Tools Table, Materials and Methods, Figures, Data Availability Section)
If study protocol has been **pre-registered, provide DOI in the manuscript**. For clinical trials, provide the trial registration number **OR** cite DOI.	Not Applicable	
Report the **clinical trial registration number** (at ClinicalTrials.gov or equivalent), where applicable.	Not Applicable	

Laboratory protocol	Information included in the manuscript?	In which section is the information available? (Reagents and Tools Table, Materials and Methods, Figures, Data Availability Section)
Provide DOI OR other citation details if **external detailed step-by-step protocols** are available.	Not Applicable	

Experimental study design and statistics	Information included in the manuscript?	In which section is the information available? (Reagents and Tools Table, Materials and Methods, Figures, Data Availability Section)
Include a statement about **sample size** estimate even if no statistical methods were used.	Yes	Materials and Methods, Figures
Were any steps taken to minimize the effects of subjective bias when allocating animals/samples to treatment (e.g. **randomization procedure**)? If yes, have they been described?	Not Applicable	
Include a statement about **blinding** even if no blinding was done.	Yes	Materials and Methods
Describe **inclusion/exclusion criteria** if samples or animals were excluded from the analysis. Were the criteria pre-established?<br><br>If sample or data points were omitted from analysis, report if this was due to attrition or intentional exclusion and provide justification.	Not Applicable	
For every figure, are **statistical tests** justified as appropriate? Do the data meet the assumptions of the tests (e.g., normal distribution)? Describe any methods used to assess it. Is there an estimate of variation within each group of data? Is the variance similar between the groups that are being statistically compared?	Not Applicable	

Sample definition and in-laboratory replication	Information included in the manuscript?	In which section is the information available? (Reagents and Tools Table, Materials and Methods, Figures, Data Availability Section)
In the figure legends: state number of times the experiment was **replicated** in laboratory.	Yes	Figures; Materials and Methods
In the figure legends: define whether data describe **technical or biological replicates**.	Yes	Figures; Materials and Methods

Ethics

Ethics	Information included in the manuscript?	In which section is the information available? (Reagents and Tools Table, Materials and Methods, Figures, Data Availability Section)
Studies involving **human participants**: State details of **authority granting ethics approval** (IRB or equivalent committee(s), provide reference number for approval.	Not Applicable	
Studies involving **human participants**: Include a statement confirming that **informed consent** was obtained from all subjects and that the experiments conformed to the principles set out in the WMA Declaration of Helsinki and the Department of Health and Human Services Belmont Report.	Not Applicable	
Studies involving **human participants:** For publication of **patient photos**, include a statement confirming that consent to publish was obtained.	Not Applicable	
Studies involving experimental **animals**: State details of **authority granting ethics approval** (IRB or equivalent committee(s), provide reference number for approval. Include a statement of compliance with ethical regulations.	Not Applicable	
Studies involving **specimen and field samples:** State if relevant **permits** obtained, provide details of authority approving study; if none were required, explain why.	Not Applicable	

Dual Use Research of Concern (DURC)	Information included in the manuscript?	In which section is the information available? (Reagents and Tools Table, Materials and Methods, Figures, Data Availability Section)
Could your study fall under dual use research restrictions? Please check biosecurity documents and list of **select agents and toxins** (CDC): https://www.selectagents.gov/sat/list.htm	Not Applicable	
If you used a select agent, is the security level of the lab appropriate and reported in the manuscript?	Not Applicable	
If a study is subject to dual use research of concern regulations, is the name of the **authority granting approval and reference number** for the regulatory approval provided in the manuscript?	Not Applicable	

Reporting

The MDAR framework recommends adoption of discipline-specific guidelines, established and endorsed through community initiatives. Journals have their own policy about requiring specific guidelines and recommendations to complement MDAR.

Adherence to community standards	Information included in the manuscript?	In which section is the information available? (Reagents and Tools Table, Materials and Methods, Figures, Data Availability Section)
State if relevant guidelines or checklists (e.g., **ICMJE, MIBBI, ARRIVE, PRISMA**) have been followed or provided.	Not Applicable	
For **tumor marker prognostic studies,** we recommend that you follow the **REMARK** reporting guidelines (see link list at top right). See author guidelines, under 'Reporting Guidelines'. Please confirm you have followed these guidelines.	Not Applicable	
For **phase II and III randomized controlled trials**, please refer to the **CONSORT** flow diagram (see link list at top right) and submit the CONSORT checklist (see link list at top right) with your submission. See author guidelines, under 'Reporting Guidelines'. Please confirm you have submitted this list.	Not Applicable	

Data Availability

Data availability	Information included in the manuscript?	In which section is the information available? (Reagents and Tools Table, Materials and Methods, Figures, Data Availability Section)
Have **primary datasets** been deposited according to the journal's guidelines (see 'Data Deposition' section) and the respective accession numbers provided in the Data Availability Section?	Yes	Data Availability Section
Were **human clinical and genomic datasets** deposited in a public access-controlled repository in accordance to ethical obligations to the patients and to the applicable consent agreement?	Not Applicable	
Are **computational models** that are central and integral to a study available without restrictions in a machine-readable form? Were the relevant accession numbers or links provided?	Not Applicable	
If publicly available data were reused, provide the respective **data citations in the reference list**.	Not Applicable	
